# The millipede tribe Brachyiulini in the Caucasus (Diplopoda, Julida, Julidae)

**DOI:** 10.3897/zookeys.1058.68628

**Published:** 2021-08-30

**Authors:** Boyan Vagalinski, Sergei I. Golovatch

**Affiliations:** 1 Institute of Biodiversity and Ecosystem Research at the Bulgarian Academy of Sciences, 2 Yurii Gagarin Street, 1113, Sofia, Bulgaria Institute of Biodiversity and Ecosystem Research at the Bulgarian Academy of Sciences Sofia Bulgaria; 2 Institute for Problems of Ecology and Evolution, Russian Academy of Sciences, Leninsky pr. 33, Moscow 119071, Russia Institute for Problems of Ecology and Evolution, Russian Academy of Sciences Moscow Russia

**Keywords:** Armenia, Azerbaijan, Georgia, key, new genus, new records, new species, Russia

## Abstract

The diplopod tribe Brachyiulini is represented in the fauna of the Caucasus by eight genera and 32 species, of which one genus and 14 species are described as new: *Colchiobrachyiulusmontanus* Vagalinski, **sp. nov.**, *Iraniulustricornis* Vagalinski, **sp. nov.**, *Omobrachyiulusarmatus* Vagalinski, **sp. nov.**, *O.fasciatus* Vagalinski, **sp. nov.**, *O.faxifer* Vagalinski, **sp. nov.**, *O.kvavadzei* Vagalinski, **sp. nov.**, *O.lazanyiae* Vagalinski, **sp. nov.**, *O.ponticus* Vagalinski, **sp. nov.**, *O.pristis* Vagalinski, **sp. nov.**, *O.trochiloides* Vagalinski, **sp. nov.**, *O.unugulis* Vagalinski, **sp. nov.**, *O.zuevi* Vagalinski, **sp. nov.**, *Svaniulusryvkini* Vagalinski, **gen. nov.**, **sp. nov.**, and *S.waltheri* Vagalinski, **gen. nov.**, **sp. nov.**Colchiobrachyiulus Lohmander, 1936, a former subgenus of Megaphyllum, is here elevated to a full genus, and the genus Grusiniulus Lohmander, 1936 is downgraded to a subgenus of the genus Cyphobrachyiulus Verhoeff, 1900, both **stat. nov.**, with their previously described species, *Colchiobrachyiulusdioscoriadis* (Lignau, 1915) and *Cyphobrachyiulusredikorzevi* (Lohmander, 1936), respectively, listed as **comb. nov.***Omobrachyiulusbrachyurus* (Attems, 1899) is formally established as a junior subjective synonym of *O.caucasicus* (Karsch, 1881), **syn. nov.**, and *Omobrachyiulusimplicitusritsensis* (Golovatch, 1981) is formally synonymised with the typical *Omobrachyiulusimplicitus* (Lohmander, 1936), **syn. nov.***Omobrachyiulussevangensis* (Lohmander, 1932), originally described in the genus *Megaphyllum*, is here transferred to the former genus, **comb. nov.** The diagnoses and descriptions of some genera and subgenera are refined and complemented. A key is given to all genera and species of Brachyiulini that occur in the Caucasus, and their distributions are mapped. Several species are recorded as new to the faunas of Armenia, Azerbaijan, Georgia, or Russia. The distribution patterns of the Caucasian Brachyiulini and their biogeographic implications are discussed.

## Introduction

The Caucasus is a vast border region in Eurasia lying between southeastern Europe and western Asia, bordered on the south by Iran, on the southwest by Turkey, on the west by the Black Sea, on the east by the Caspian Sea, and on the north by Russia. Two main parts are distinguished: the Caucasus Major, or the Greater Caucasus, represented by the Main Caucasus Ridge in the north, and the Caucasus Minor, or the Lesser Caucasus, in the south. The Caucasus includes the southern parts of European Russia (Rostov-on-Don Region (with its southernmost parts), Krasnodar and Stavropol provinces, Adygea, Karachay-Cherkess, Kabarda-Balkar, North Ossetia-Alania, Ingush, Chechen, and Dagestan republics), as well as three states in Transcaucasia: Georgia (together with the autonomous republics Abkhazia and Ajara = Ajaria), Armenia, and Azerbaijan. The immediately adjacent parts of Turkey and northwestern Iran are often considered by biologists as belonging to the Caucasus as well (e.g., Abdurakhmanov 2017).

According to the most recent estimates, the millipede fauna of the Caucasus is known to comprise > 160 species, > 50 genera, 14 families, and eight orders. Endemism at the species level is overwhelming, amounting to > 85%, while as many as 25 millipede genera are endemic or subendemic to the Caucasus. All families and orders they belong to, however, are widely distributed at least across the Euro-Mediterranean realm (Antić and Reip 2020).

Two review papers summarising our knowledge of the millipede fauna of the Caucasus, and both discussing biogeographic issues as well, one by Muralewicz (1911) and the other by Lohmander (1936), are vastly outdated and have since been rectified, modernised, and heavily updated. Thus, the Caucasian Polyxenida (Short 2015; Short et al. 2020), Glomerida (Golovatch 1989a, b, 1990a, 1999; Golovatch and Turbanov 2017, 2018), Colobognatha (Golovatch et al. 2015), Polydesmida (Evsyukov et al. 2016; Golovatch et al. 2016), and Chordeumatida (Antić and Makarov 2016; Antić et al. 2018), as well as the families Blaniulidae (Enghoff 1984, 1990; Golovatch and Enghoff 1990) and Nemasomatidae (Enghoff 1985), both in Julida, have been thoroughly revised. As regards the Julidae, the only other family of Julida remaining in the Caucasus, it is definitely one of the most diverse, common, and widespread across the region. The following tribes have already been reviewed and treated taxonomically in the scope of the entire Caucasian fauna: Cylindroiulini (Read 1992; Evsyukov and Golovatch 2013; Zuev 2014), Pachyiulini (Evsyukov 2016; Evsyukov et al. 2021), Julini (Evsyukov et al. 2018), Leptoiulini (Evsyukov et al. 2020), and Leucogeorgiini (Antić and Reip 2020).

The Euro-Mediterranean and mostly epigean tribe Brachyiulini is the target of the present contribution. With currently > 100 species described (Vagalinski and Lazányi 2018), it is one of the largest julid tribes, being outnumbered only by the Leptoiulini and the Cylindroiulini. Brachyiulinines dominate the millipede faunas of the Balkans and Anatolia, especially the Aegean region which is shared between the two peninsulas. Until now, only 18 species of Brachyiulini have been known from the Caucasus, originally described and/or recorded in the region by Karsch (1881), Timotheew (1897), Lignau (1903, 1915), Verhoeff (1921), Lohmander (1928, 1932, 1936), Golovatch (1981), and Zuev (2014). The new data presented in this paper are based upon abundant, hitherto unpublished material originating from all Caucasian countries, and collected between 1932 and 2016.

## Materials and methods

All examined material is preserved in 70% ethanol, with particular body parts of some of the type specimens mounted on permanent microscopic slides with Euparal medium. Gonopods of all species represented by more than one male were prepared for scanning electron microscopy (SEM).

The diagnosis of the tribe Brachyiulini and the diagnoses of the genera and subgenera treated here are updated versions of those found in Lazányi and Vagalinski (2013), and in Vagalinski and Lazányi (2018). The descriptions mostly follow the pattern and terminology used by Vagalinski and Lazányi (2018), and by Vagalinski and Golovatch (2019). Developmental stadia are determined using the eye-row method, i.e., by counting the number of vertical rows of ommatidia and adding 1 (see Enghoff et al. 1993). The symbols ‘>’, ‘<’, and ‘=’ concerning antennomeres refer to their relative lengths.

Identifications and general observations were made under a МБС-10 stereo microscope. Colour pictures of type specimens were prepared by focal stacking of multilayer photographs taken with the aid of a Carl Zeiss Discovery V8 stereo microscope with a Nikon Coolpix S3700 camera mounted on one of the eyepieces. Black-and-white micrographs of various body parts were taken with a ProgRes C7 camera connected to a Zeiss Axio Imager 2 compound microscope. Part of these images were represented as line drawings, after copying with the aid of tracing paper placed on a laptop screen. SEM micrographs were taken with a JEOL JSM-5510 or a JEOL JSM-6510LV scanning electron microscope after mounting on SEM stubs or cover glasses and sputter-coating with gold. Blank maps were generated in ArcGIS and the species distributions were subsequently mapped using Photoshop CC 2019. All image processing was made using Photoshop CC 2019.

All included species are presented with their short synonymy lists (the original combination and the subsequent mentions of any other combination existing in the literature, these concerning only the Caucasian fauna) and known distribution records, except for some more common ones whose distributions are summarised.

Apart from the Caucasus proper (i.e., the two main mountain ranges, the Greater and the Lesser Caucasus), the current study also encompasses the immediately adjacent regions of Ciscaucasia (including its lower parts) and Colchis, which belong to the same biogeographic province. The Hyrcan biogeographic province within both the Republic of Azerbaijan and Iran and the eastern parts of the Pontic Mountains in Turkey, despite often considered as part of the Caucasus sensu lato (see Introduction), are here treated as separate regions and thus not included in the distribution maps. However, unpublished material of the only brachyiulinine to occur in Hyrcania, *Iraniulusfagorum* (Attems, 1951), is also included in the paper, to compare it with a newly described Caucasian congener, *I.tricornis* sp. nov.

### Repositories of the examined material

**AE** Private collection of Aleksandr Evsyukov, Rostov-on-Don, Russia;

**IBER** Institute of Biodiversity and Ecosystem Research, Sofia, Bulgaria;

**HNHM**Hungarian Natural History Museum, Budapest, Hungary;

**NHMD**Natural History Museum of Denmark, Copenhagen, Denmark (formerly ZMUC, Zoological Museum, University of Copenhagen);

**NMNHS**National Museum of Natural History, Sofia, Bulgaria;

**SMNG**Staatliches Museum für Naturkunde, Görlitz, Germany;

**ZIN**Zoological Institute, Russian Academy of Sciences, St. Petersburg, Russia;

**ZMB**Müseum für Naturkunde, Berlin, Germany;

**ZMUM** Zoological Museum, State University of Moscow, Moscow, Russia.

### Local endemic chorotypes

**CAUC** broad endemic to the Caucasus region, distributed on both sides of the Greater Caucasus watershed, and both east and west of Mount Kazbek;

**CECA** central Caucasian endemic (the valley of Kura River and the southern and northern slopes of the Greater and Lesser Caucasus, respectively, east of Likhi (= Surami) Mountain Range);

**COLC** endemic to Colchis (between the southern and northern foothills of the Greater and Lesser Caucasus, respectively, west of Likhi Mountain Range);

**GRCA** endemic to the Greater Caucasus (both east and west of Mount Kazbek);

**LECA** endemic to the Lesser Caucasus;

**NWGC** endemic to the northwestern parts of the Greater Caucasus (north of the Caucasus Major watershed and west of Mount Kazbek);

**SWGC** endemic of the southwestern parts of the Greater Caucasus (south of the Caucasus Major watershed and west of Mount Kazbek);

**WCIS** endemic to western Ciscaucasia (approximately west of the line between the Stavropol Highland and the Pyatigorsk Mountains);

**WECA** broad endemic to the western parts of the Caucasus region (west of Mount Kazbek, on both sides of the Greater Caucasus watershed including parts of Ciscaucasia, Cochis, or Lesser Caucasus).

### Other abbreviations

**AR** Autonomous Republic

**CBO** central body of opisthomere

**SIG** Sergei I. Golovatch

### Symbols used in the species descriptions

**L** body length measured at the ozopore level

**H** vertical diameter at mid-body

**S** developmental stadium

**n*_Schub_*** number of striae over a distance equal to the length of the metazona, counted just below the ozopore level in a mid-body ring (after Schubart (1934), slightly modified).

**T** telson

All abbreviations of gonopodal and vulval structures are explained in the figure captions. More detailed explanations of the main gonopodal and vulval features in the Brachyiulini can be found in Vagalinski and Lazányi (2018).

## Taxonomic part

### Genus Brachyiulus

Taxon classificationAnimaliaJulidaJulidae

Berlese, 1884

D0C47877-96A2-5E97-8B66-0A179C0ECD58

#### Updated diagnosis.

A genus of Brachyiulini differing from contribal genera by the following combination of characters: promeres positioned completely anteriorly in relation to opisthomeres; promere very short, on average half as long as opisthomere; opisthomere possessing a clearly discernible basoposterior process, the latter sometimes tightly contiguous with CBO, a usually well-pronounced lateral process (very small in some species), which is strongly mesolaterally flattened, and an anterior process; some species with a mesoanterior process.

### Brachyiulus jawlowskii

Taxon classificationAnimaliaJulidaJulidae

Lohmander, 1928

A9374A20-CE68-5962-B8CE-FEA4765A2607

Brachyiulus jawlowskii Lohmander, 1928: 536–538, fig. 8.
Brachyiulus jawlowskii : Zuev 2014: 351.

#### Diagnosis.

Differs from its only congener known from the Caucasus, *B.lusitanus* Verhoeff, 1898, mainly by the opisthomere possessing a smooth rather than striated lateral process, and a slightly rather than strongly bent anterior process.

#### Material examined.

2 ♂♂, 8 ♀♀ (ZMUM), **Georgia**: AR Abkhazia, Pitsunda, Bzyb River Valley, meadow with a few *Buxus* trees, in litter, 8.IV.1983, SIG leg.

#### Previous records from the Caucasus.

**Russia**: southern Rostov-on-Don Region and Stavropol (Evsyukov and Golovatch 2013; Zuev 2014).

#### General distribution.

From eastern Poland in the west to southwestern Siberia, Russia and western Kazakhstan in the east (Nefediev et al. 2014, Kime and Enghoff 2017); subendemic to the Russian [= East European] Plain (Wytwer et al. 2009; Zuev 2014).

#### Remarks.

The species is new to the fauna of AR Abkhazia. It has already been known from the Caucasus region, reported by Zuev (2014) from a farm in the city of Stavropol, while the current record marks the southernmost locality of its known range. The occurrences of this species both at Stavropol and in Abkhazia seem to be synanthropic.

### Brachyiulus lusitanus

Taxon classificationAnimaliaJulidaJulidae

Verhoeff, 1898

7824503F-8F60-5F0A-BECD-12713484DCD0

Brachyiulus pusillus , lusitanus (sic!) Verhoeff, 1898: 153–154, fig. 28.
Brachyiulus lusitanus : Bababekova 1996: 90; Kokhia and Golovatch 2018: 40; 2020: 204.
Brachyiulus lusitanus calcivagus : Lohmander 1936: 170, 179; Samedov et al. 1972: 1245, 1246; Rakhmanov 1972: 116.

#### Diagnosis.

Differs from its only congener known from the Caucasus, *B.jawlowskii*, mainly by the opisthomere possessing a striated rather than smooth lateral process, and a strongly rather than slightly bent anterior process.

#### Material examined

**(ZMUM). Georgia**: 7 ♂♂, 1 ♀, AR Abkhazia, region of Sukhumi, Nizhnyaya Yashtukha, tobacco plantation, 26.V–19.VI.1981, A. Markosyan leg.; 1 ♂, Tbilisi, canyon of Vere River, 18.X.1980, M. Kokhia leg.; 2 ♂♂, 2 ♀♀, eastern Georgia, Akhmeta District, E of Kasristskali, salty swamp, reed thickets, 7.V.1983, V. Yanushev leg.

#### General distribution.

Subcosmopolitan (Kime and Enghoff 2017, Vagalinski and Lazányi 2018).

#### Remarks.

This ubiquitous, largely anthropochoric species was first listed by Lohmander (1936) and then repeated by Kokhia and Golovatch (2018) from central Georgia, Caucasus, but without any exact locality. Rakhmanov (1972) recorded this species from near Lankaran (= Lenkoran) in Hyrcania, southeastern Azerbaijan.

### Genus Byzantorhopalum

Taxon classificationAnimaliaJulidaJulidae

Verhoeff, 1930

936E2909-C85B-5EDA-9A52-51B536470565

#### Updated diagnosis.

A genus of Brachyiulini differing from contribal genera by the following combination of characters: promeres positioned completely anteriorly in relation to opisthomeres; opisthomere with a mostly vestigial or lobe-like basoposterior process, sometimes bearing one or two apical outgrowths; and a well-developed lateral process; anterior process absent.

### Byzantorhopalum (Byzantorhopalum) rossicum

Taxon classificationAnimaliaJulidaJulidae

(Timotheew, 1897)

0EA6D72F-9F28-53A2-94CD-29A19FE88E39

Fig. 1

Iulus rossicus Timotheew, 1897: 284–291, figs 21–31.
Chromatoiulus (Donbrachyiulus) rossicus : Lohmander 1936: 109–112.
Chromatoiulus (Donbrachyiulus) rossicus rossicus : Lokšina and Golovatch 1979: 385. Megaphyllumrossicumrossicum: Golovatch 1984: 103–105, 111, 125–127, 130, fig. 5. Megaphyllumprocerum: Golovatch 1990b: 364.
Megaphyllum rossicum : Golovatch 1990b: 362, 364; Loginova 1993: 151–153; Golovatch and Matyukhin 2011: 116; Zuev 2014: 352, map 4.
Byzantorhopalum (Byzantorhopalum) rossicum : Vagalinski and Lazányi 2018: 27–28, figs 11, 12, 17, 26.

#### Material examined.

**Azerbaijan**: 1 ♂ (SMNG), İsmayıllı District, S of Zərgəran, 40.7310°N, 48.3680°E, 880 m a.s.l., slope with *Corylus*, *Clematis* and some *Prunus* trees, stone heaps overgrown by moss, mainly in thick litter and under stones, 30.III.2015, D. Antić and H. Reip leg.; 1 ♀ (SMNG), İsmayıllı District, near Qurbanəfəndi, 1.3 road km towards Xanəgah, 40.8614°N, 48.1132°E, 640 m a.s.l., slope with *Fagus* and a single *Populus* tree, in litter, same date and collectors; 1 ♂, 3 ♀♀, 2 juv. (ZMUM), Altıağaç National Park, 1050–1100 m, mixed broadleaved forest, litter, 20 and 26.IV.1987, SIG and K. Eskov leg.; many ♂♂, ♀♀, juv. (ZMUM), SW of Quba, 750 m, *Fagus*, *Quercus*, *Carpinus* etc. forest, litter and under bark, 23.IV.1987, SIG and K. Eskov leg.; 1 ♂, 2 ♀♀ (ZMUM), Shemakha District, Pirkuli, 1700 m, *Quercus*, *Taxus*, 22.XI.1962, T. Perel leg. **Georgia**: 1 ♂, 2 ♀♀ (SMNG), Mtskheta-Mtianeti, valley of Andaki River, 2.2 km upstream of its confluence with Argun River, 42.5006°N, 45.2156°E, 1430 m a.s.l., 08.VIII.2014, F. Walther leg.; 1 ♂, 1 ♀ (SMNG), Mtskheta-Mtianeti, 1 km downstream of Mutso, 42.5006°N, 45.2156°E, 1530 m a.s.l., 9.VIII.2014, F. Walther leg.; 1 ♂, 1 ♀ (SMNG), NW of Stepantsminda, 42°40'1"N, 44°36'49"E, 2093 m a.s.l., *Juniperus*, 02.VII.2019, K. Voigtländer leg.; 2 ♂♂, 3 ♀♀ (ZMUM), Stepantsminda, 8.VI.1974, M. Kokhia leg. **Russia**: 1 ♂ (SMNG 33575), Republic of Adygea, Novoprokhladnoye, 20.V.2004, K. Voigtländer leg.; 2 ♂♂, 1 ♀ (ZMUM), Stavropol Province, Kursavka, *Acacia* and *Rosa* hedge alongside a road, sifted litter, 30.V.1982, SIG leg.; 2 ♀♀ (SMNG), Stavropol, Tamanskaya Dacha Forest, near Komsomolskiy Reservoir, 45.0481°N, 41.9567°E, 530 m a.s.l., deciduous forest (*Quercus*, *Carpinus*, *Acer*), 22.VIII.2012, F. Walther leg.; many ♂♂, ♀♀ (ZMUM), Stavropol Province, Novoaleksandrovsk, east end, X.2013, B. Korotyaev leg.; 4 ♂♂, 1 ♀ (ZMUM), Kabardino-Balkar Republic, Chegemsky District, upper course of Chegem River, meadow, under stones, 1500 m, 12.VII.1986, SIG leg.; 3 ♂♂, 2 ♀♀ (SMNG), Republic of North Ossetia – Alania, Alagirsky District, Borzikau, near Fiagdon River, 1200 m a.s.l., 42°50'37"N, 44°18'44"E, under stones, 11.V.2016, A.S. Sazhiev leg.; 3 ♂♂, 3 ♀♀ (ZMUM), Republic of North Ossetia-Alania, North Ossetian State Nature Reserve, Tsey Valley, 23.VII.1984, S. Alekseev leg.; many ♂♂, ♀♀, juv. (ZMUM), Chechen Republic, Kharachoy, SE of Vedeno, 950 m, *Fagus*, *Carpinus* etc. forest, in litter, under bark and stones, 17.VII.1986, SIG leg.; many ♂♂, ♀♀, juv. (ZMUM), Chechen Republic, Argun Valley, 5 km N of Shatoy, *Corylus*, *Fagus*, *Carpinus* etc. forest, 750 m, in litter, under stones and bark, 18.VII.1986, SIG leg.; 4 ♂♂, 7 ♀♀ (ZMUM), 8 ♂♂, 10 ♀♀ (ZMUM), Republic of Dagestan, Kazbekovskiy District, Dylym, *Fagus* and *Carpinus* forest, 13.VII.1985, K. Khajialiyev leg.; 8 ♂♂, 10 ♀♀ (ZMUM), Republic of Dagestan, Almak, *Carpinus* forest, 15.VII.1985, K. Khajialiyev leg.; 6 ♂♂, 5 ♀♀ (ZMUM), Republic of Dagestan, Sergokalinskiy District, Murguk, *Quercus* forest, western slope, 4.III.1985, K. Khajialiyev leg.; 10 ♂♂, 10 ♀♀, Republic of Dagestan, Kayakentskiy District, *Quercus* forest, 27.VII.1985, K. Khajialiyev leg.; 4 ♂♂, 7 ♀♀, Republic of Dagestan, Botlikh Distrikt, Kharami Pass, 2150 m, subalpine meadow, under stones, 16.VII.1986, SIG leg.; 1 ♂, 1 ♀ (HNHM), Republic of Dagestan, Kurush, 3000 m a.s.l., 8.VII.1989, Z. Korsós leg.

#### Descriptive notes.

**Figure 1.**
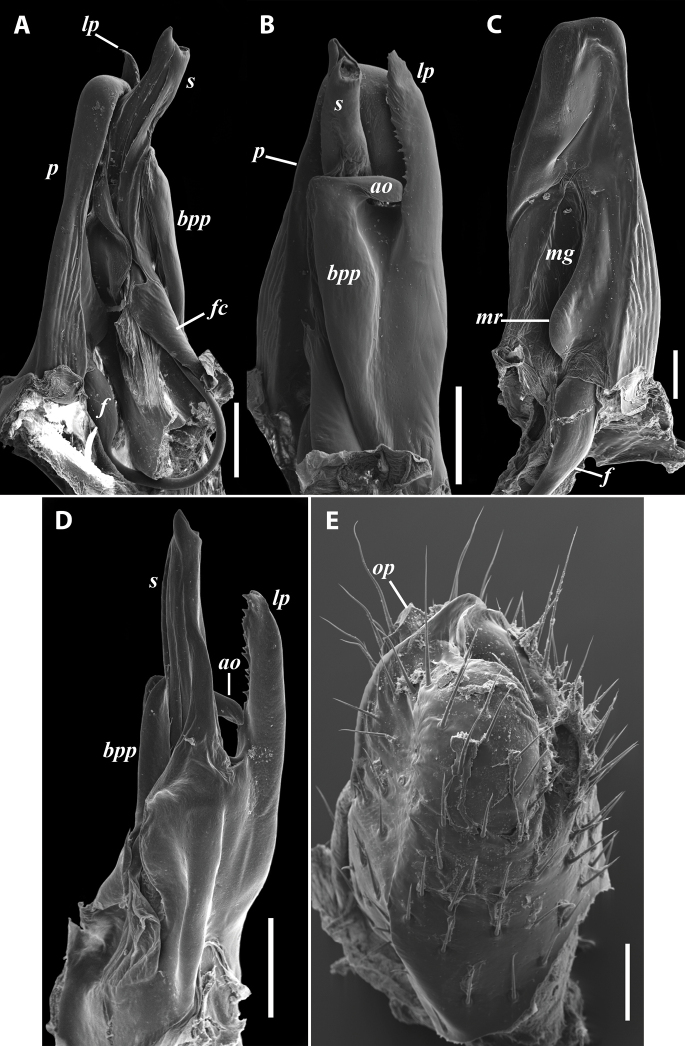
*Byzantorhopalumrossicum* (Timotheew, 1897), ♂ from S of Zərgəran (**A–D**) and ♀ from near Qurbanəfəndi (**E**), both in Azerbaijan (SMNG) **A** left gonopods, mesal view **B** same, caudal view **C** right promere, caudal view **D** right opisthomere, latero-oral view **E** right vulva, latero-caudal, somewhat apical view. Scale bars: 0.2 mm (**A, B, D**), 0.1 mm (**C, E**). Abbreviations: ***bpp*** basoposterior process, ***f*** flagellum, ***fc*** flagellum channel, ***lp*** lateral process, ***mg*** median groove, ***mr*** median ridge, ***op*** operculum, ***p***: promere, ***s*** solenomere. Pictures by courtesy of Karin Voigtländer, Hans Reip and Dragan Antić.

***Gonopods*:** Promere (Fig. 1C and p in Fig. 1A, B) somewhat shorter than solenomere, with a well-developed median ridge and a deep and narrow median groove; flagellum of usual thickness; opisthomere (Fig. 1A, B, D) with a large lateral process with serrated mesal margin, and a basoposterior process with an apical outgrowth turned laterad; solenomere long (significantly exceeding promere), tubular, with a frontoapical acumination and a groove behind it; neither set- nor spiniform filaments at flagellum channel. ***Vulva*** (Fig. 1E) densely setose throughout, with operculum subequal to bursa.

#### Previous records from the Caucasus.

**Russia**: Environs of Kislovodsk (Timotheew 1897), many places across the northern Caucasus (Lohmander 1936), Stavropol Province (Zuev 2014).

#### General distribution.

Also known from eastern Ukraine, Crimea, and the Kursk, Orel, Voronezh, Belgorod, Rostov-on-Don, Samara, Volgograd, and Saratov regions in central and southern European Russia (Lokšina 1969; Prisnyi 2001).

The subspecies *B.r.strandschanum* (Verhoeff, 1937) occurs in southeastern Bulgaria and northeastern Greece (Lazányi et al. 2012; Kime and Enghoff 2017). Despite its markedly disjunct distribution in relation to that of the nominate subspecies, until now, no reliable morphological characters to distinguish between the two taxa have been proposed (see also Vagalinski and Lazányi 2018). It thus seems that the usage of molecular data is the only way to define the nomenclatural faith of *B.r.strandschanum*.

#### Remarks.

The species is new to the faunas of Georgia and Azerbaijan.

### Genus Colchiobrachyiulus

Taxon classificationAnimaliaJulidaJulidae

Lohmander, 1936 stat. nov.

D6B023DA-DDAC-5F6B-9FFD-E384D67F0509

#### Updated diagnosis.

A genus of Brachyiulini differing from contribal genera by the unique arrangement of the gonopod parts, including the promeres being positioned completely laterally rather than anteriorly in relation to the opisthomeres, and the solenomere being protected by specific grooves/channels in the anterior process of the opisthomere and in the distomesal process of the promere; as well as by the following combination of other characters: opisthomere with a lobe-like basoposterior process being mostly fused to CBO, ending with a freely protruding, branched, papillose, apical outgrowth, a well-developed anterior process, partially enveloping the solenomere, and a very slender, tapering solenomere, without any distinct apical structures.

#### General description.

- Small to medium-sized (L (males) = 15–37 mm) Brachyiulini.
- Ommatidia present.
- Ozopores right on or tightly behind pro-metazonal suture at least on more anterior body rings.
- Epiproct well-developed, from moderately to relatively (not conspicuously) long.
- Male hypoproct rounded trapezoidal to semi-circular, ventrally with two distal paramedian setae.
- Male mandibular stipites considerably expanded, broadly rounded, without a distinct anterior/anteroventral corner.
- Male walking legs ventrally with two well-developed adhesive pads, one each on postfemur and tibia.
- Penis short and stout, with very short apical lobes and small terminal lamellae.
- Gonopods:

- In situ considerably protruding from gonopodal sinus, directed caudoventrad.
- Promere higher than opisthomere, elongate, bearing a slender distomesal process with a narrow channel connected with a deep distomesal groove on caudal sur face of promere, both designed to envelop the solenomere; median ridge and median groove rather poorly developed; flagellum thin, micro-dentate apically.
- Opisthomere rather elongate; basoposterior process weakly pronounced, mostly fused to CBO, ending in a finely branched, papillose, freely protruding, apical outgrowth; anterior process long and flattened, partly enveloping the solenomere; an apicoposterior or lateral process, and a mesomeroidal lobe absent; solenomere fine and slender, with a simple tubular structure; with long spiniform filaments along flagellum channel.

- Vulva:

- Subcylindrical, mostly symmetrical.
- Bursa with a distinct, more or less obtuse, postero-apical margin.
- Opening placed apically on bursa.
- Operculum subequal in height to bursa.
- Receptaculum seminis: central tube narrow; posterior tube long and very narrow, somewhat folded; posterior ampulla small to medium-sized.

#### Comment.

Vagalinski and Lazányi (2018), in their revision of the tribe Brachyiulini, retained *Colchiobrachyiulus* as a subgenus of *Megaphyllum* due to its apparent correspondence to the diagnosis of the latter genus. However, the examination of the present material of both *C.dioscoriadis* (Lignau, 1915), comb. nov. and *C.montanus* sp. nov. shows that the two species can hardly be regarded as close relatives of *Megaphyllum* s. str., and the unique arrangement of the gonopodal apparatus alone is sufficient to warrant *Colchiobrachyiulus* the status of a full genus. Besides this, certain morphological characters of *Colchiobrachyiulus* suggest proximity to *Omobrachyiulus* Lohmander, 1936: a lobe-like, basoposterior process of the opisthomere ending in a shield-like, apical outgrowth, branched or dentate at margin, a fine and slender solenomere, and the presence of long, erect and spiniform filaments along the flagellum channel are seen in many species of *Omobrachyiulus*. Nevertheless, the absence of an opisthomeral mesomeroidal lobe in *Colchiobrachyiulus* seems to be a sufficiently significant difference from *Omobrachyiulus*.

#### Remark.

Both the distomesal process of the promere and the anterior process of the opisthomere in *Colchiobrachyiulus* seem to be specially designed for providing protection to the solenomere, enveloping the latter from the lateral and caudo-lateral sides, respectively. It is also possible that one or both of these structures take part in copulation, facilitating the penetration of the solenomere into the vulval opening.

### Colchiobrachyiulus dioscoriadis

Taxon classificationAnimaliaJulidaJulidae

(Lignau, 1915) comb. nov.

A7C20728-BA76-5834-8D80-2957B1DF343E

Figs 2 , 3

Brachyiulus dioscoriadis Lignau, 1915: 382–387, text figs 12–20, tab. IV: figs 4, 5.
Chromatoiulus (Colchiobrachyiulus) dioscoriadis : Lohmander 1936: 113–114.
Chromatoiulus dioscoriadis : Kobakhidze 1964: 191; 1965: 393.
Megaphyllum dioscoriade (sic!): Talikadze 1984: 143.
Megaphyllum (Colchiobrachyiulus) dioscoriadis : Vagalinski and Lazányi 2018: 90–91, fig. 182.
Megaphyllum dioscoriadis : Chumachenko 2016: 409; Kokhia and Golovatch 2018: 40; 2020: 206.

#### Material examined

**(ZMUM). Georgia**: AR Abkhazia: 2 ♂♂, 1 juv., Gulripsh near Sukhumi, 24.X.1953, E. Borutzky leg.; 2 ♂♂, 2 ♀♀, Ochamchira District, Jgerda, Kodorskiy Mountain Range, in litter and under bark, 21.IX.1985, I.A. Ushakov leg.; 2 ♂♂, 3 ♀♀, Pitsunda-Myussera Nature Reserve, Myussera part, 20–130 m, mixed deciduous forest (*Castanea*, *Alnus*, etc.), in litter, under bark and stones, 8–10.IV.1983, SIG leg.; 1 ♂, 1 ♀, Pskhu-Gumistinskiy Nature Reserve, Gumistinskiy part, cordon Nizhniy Tsumur, 25.IX.1985, I.A. Ushakov leg.; 3 ♂♂, 1 ♀, 1 juv., Sukhum District, Tsebelda, 300 m a.s.l., *Carpinus*, *Acer* and *Buxus* scrub, in litter, 19.VIII.1986, SIG leg.; 6 ♂♂, 11 ♀♀, 3 juv., Sukhum District, Lake Amtkelis (Azanta) ca. 16 km N of Tsebelda, 550 m, *Alnus* forest, litter and under bark, 19.VIII.1986, SIG leg.

#### Diagnosis.

A species of *Colchiobrachiulus* differing from its single known congener, *C.montanus* sp. nov., by the larger size (both sexes > 30 mm in length and 2 mm in height, vs. males < 20 mm in length and 1.3 mm in height, and females < 25 mm in length and 2 mm in height in *C.montanus* sp. nov.), the longer epiproct, and by certain details of gonopodal structure, more specifically, by the broader apex of the promere and its distomesal process significantly outreaching the apex, vs. the same being subequal to the apex in *C.montanus* sp. nov.; by the apical outgrowth of the opisthomere’s basoposterior process being relatively broader, with shorter branches at margin; and by the solenomere being bi- rather than unipartite in its distal section.

#### Descriptive notes.

**Figure 2.**
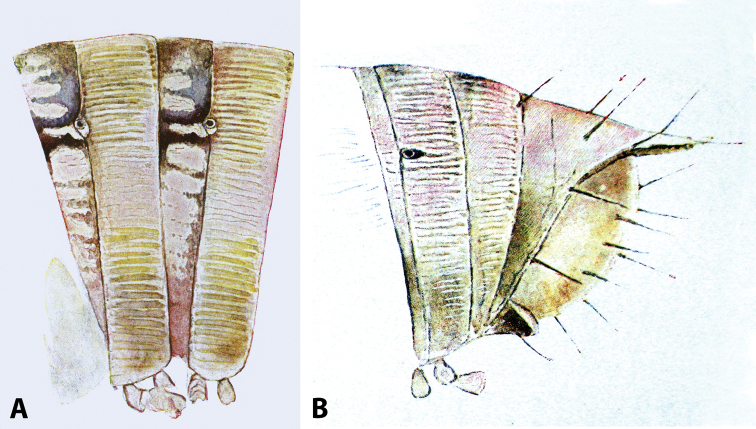
*Colchiobrachyiulusdioscoriadis* (Lignau, 1915), comb. nov. **A** mid-body rings **B** caudal end, lateral views. Not to scale. After Lignau (1915).

***Colouration*** (Fig. 2) mostly brown with reddish tinges; prozonae darker above ozopore level, contrasting lighter below it; metazonae more regularly brown-beige.

***Epiproct*** (in Fig. 2B) rather long, straight or slightly bent dorsad.

**Figure 3.**
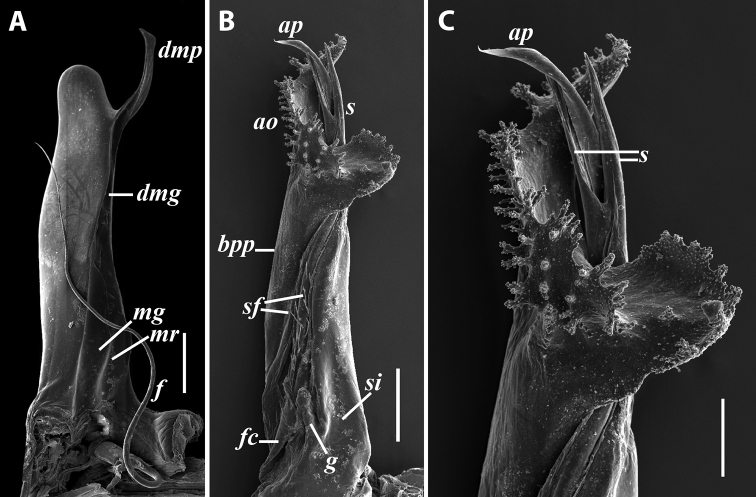
*Colchiobrachyiulusdioscoriadis* (Lignau, 1915), gonopods of ♂ from near Lake Amtkelis, Georgia (ZMUM) **A** right promere, caudal view **B** right opisthomere, mesal view **C** apical part of same aspect. Scale bars: 0.2 mm (**A, B**), 0.1 mm (**C**). Abbreviations: ***ap*** anterior process, ***ao*** apical outgrowth, ***bpp*** basoposterior process, ***dmg*** distomesal groove, ***dmp*** distomesal process, ***f*** flagellum, ***fc*** flagellum channel, ***g*** (supposed) gonocoxal gland, ***mg*** median groove, ***mr*** median ridge, ***s*** solenomere, ***sf*** spiniform filaments, ***si*** anteromesal sinus.

***Gonopods*** (Fig. 3): Promere (Fig. 3A) slender, slightly tapering towards a broadly rounded apex; mesal margin distally bearing a long and fine process significantly exceeding the main promeral apex; caudal surface with a weakly pronounced median ridge, a shallow and rather narrow median groove, and a deep and narrow distomesal groove; flagellum slightly longer than height of promere, apically micro-dentate. Opisthomere (Fig. 3C, E–H) with a basoposterior process in the shape of a broad lobe running parallel to ending in a somewhat fan-like, apical outgrowth bearing numerous minute, papillose branches; anterior process long and slender, twisted at nearly 90° around solenomere, reaching somewhat higher than the latter; mesal side with a small lobe (presumably gonocoxal gland), and a shallow anteromesal sinus frontal to the gland; two sparse rows of not too long spiniform filaments at basomedial section of flagellum channel; solenomere slender, distally divided in two pointed apices of similar size.

#### Previous records from the Caucasus.

**Georgia**: AR Abkhazia: New Athos Monastery [S of Armianskoe Ushchelie]; at Besla [Basla] River [by Sukhumi]; Kamani Monastery [ca. 3 km N of Sukhumi]; Tsebelda; Lata; by the influx of Chkhalta River in Kodori River; village “Aschary” [Azhara]; on the right bank of Chkhalta River; near the influx of Gvandra River in Sekeni [must be Kodori] River (original localities); Cave Mikhailovskaya near Sukhumi (Lohmander 1936); Tsebelda; Klych River valley, near waterfalls (Kobakhidze 1965). [?!] **Russia**: Karachay-Cherkess Republic, influx of Marukha and “Adynge” rivers at 1400 m a.s.l. (original locality).

#### General distribution.

COLC-SWGC.

#### Remarks.

The original record from the valley of Marukha River in the Karachay-Cherkess Republic, Russia almost surely concerns *Colchiobrachyiulusmontanus* sp. nov. All remaining records of *C.dioscoriadis* comb. nov. come from the Colchis Lowland and the southwestern foothills of the Greater Caucasus, and it is fairly unlikely that the species appears sympatric with its single known congener on the other side of the main Caucasus Major watershed.

Some of the examined females had large chunks of tar-like brownish substance behind leg pair 2, tightly stuck to the distal parts of the vulvae and covering the opening. These most likely represent ‘copulatory plugs’ (see, e.g., Shear 2010; Enghoff 2018) or, more precisely, in this case ‘copulatory caps’.

### Colchiobrachyiulus montanus

Taxon classificationAnimaliaJulidaJulidae

Vagalinski sp. nov.

0A043F07-F4C1-5996-B58F-4FC060CC244B

http://zoobank.org/09837D81-B854-4E35-912F-3FED58E08400

Figs 4 , 5

#### Material examined

**(all from Russia, Karachay-Cherkess Republic, Teberda Biosphere Nature Reserve). *Holotype***: ♂ (ZMUM) (unbroken), Mount Mussa-Achitara in Dombai, 2700–2800 m, alpine meadow, under stones, 29.VII.1986, SIG leg. ***Paratypes***: 1 ♂ (ZMUM) (head to ring 6 and rest of body, gonopods prepared for SEM), 2 ♀♀ (ZMUM) (one unbroken, the other with head to ring 3 and rest of body, left vulva dissected), same collecting data as for holotype; 1 ♂ (ZMUM) (head to ring 2 (head damaged, gnathochilarium separated), rings 3 to 6, and rest of body (half-broken in the middle, gonopods dissected)), 2 ♀♀ (ZMUM) (one unbroken, the other broken into two pieces), Alibek Canyon near Dombai, 2000–2100 m, sparse *Betula* stand, litter, under stones, 25.VIII.1986, SIG leg.; 1 ♂ (NMNHS) (head to ring 3, ring 4 to ring 6, pleurotergum 7, and rest of body, penis and gonopods dissected), 1 subad. ♂ (NMNHS) (in 2 pieces), Arkhyz part, VI–VII.1988, A.P. Zolotarev leg.

#### Diagnosis.

A species of *Colchiobrachiulus* differing from its single known congener, *C.dioscoriadis* comb. nov., by its smaller size (males < 20 mm in length and 1.3 mm in height, mature females < 25 mm in length and 2 mm in height, vs. > 30 mm in length and 2 mm in height for both sexes in *C.dioscoriadis* comb. nov.), by a shorter epiproct, and by gonopodal details, more specifically by the more narrow apex of the promere and its distomesal process being subequal to the apex, vs. that same process significantly outreaching the apex in *C.dioscoriadis* comb. nov.; by the apical outgrowth of the opisthomere’s basoposterior process being relatively more narrow, with longer branches at margin; and by the solenomere being uni- rather than bipartite in its distal section.

#### Name.

Emphasising the high-mountainous occurrence of this species. Adjective.

#### Description.

***Measurements*:** holotype ♂ in S XI, 43+1+T, L = 16 mm, H = 1.2 mm; paratype ♂♂ in S X, 42–45+1+T, H = 1.15–1.2 mm; paratype ♀♀ in S X–XI, 42–45+1+T, L = 18–22 mm, H = 1.5–1.85 mm.

**Figure 4.**
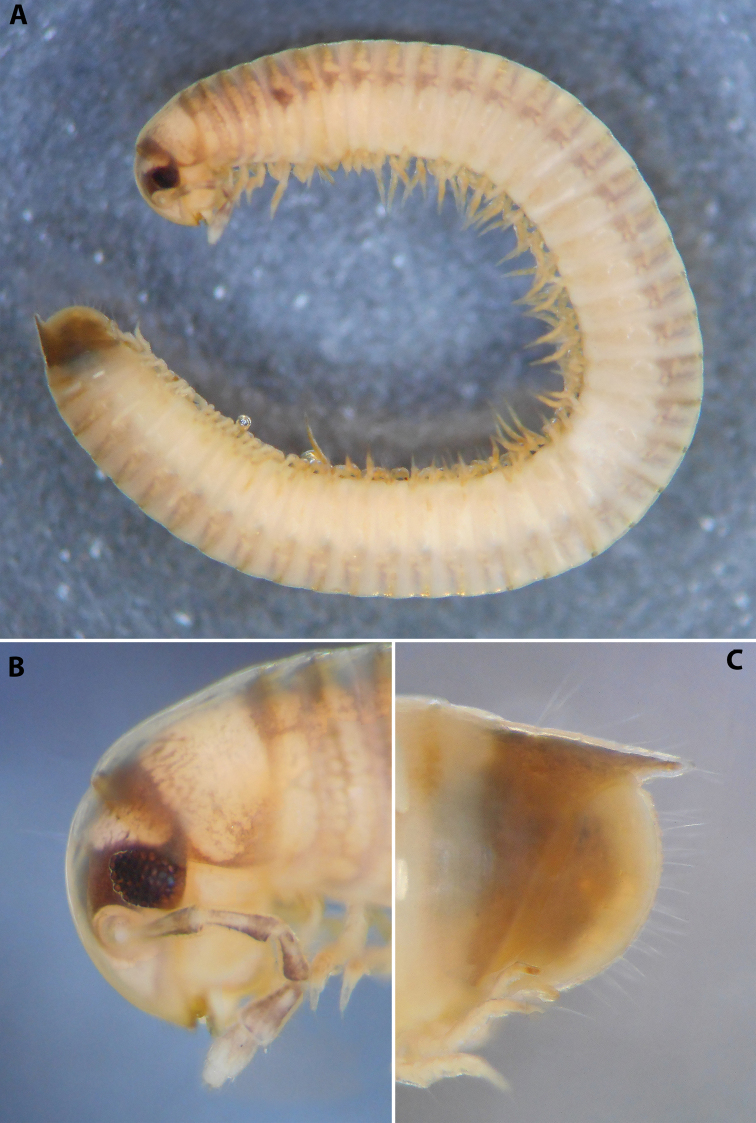
*Colchiobrachyiulusmontanus* sp. nov., ♂ holotype **A** habitus **B** head and body rings 1–3 **C** telson, lateral views. Not to scale.

***Colouration*** (after > 30 years of preservation in alcohol) (Fig. 4): Apparently heavily faded, mostly brown; head and collum (Fig. 4B) with a usual colour pattern; antennae brownish; prozonae dark brown above ozopore level, each with several uneven light spots dorsally, the largest one right above ozopore; metazonae lighter brown-beige; dorsum with a blackish axial line; legs brown; pre-anal ring dark brown, contrasting to the lighter previous ring, paraprocts proximally dark brown, distally lighter.

***External structures*:** Eye patches in adults consisting of 35–40 ommatidia arranged in easily countable vertical rows. Vertigial, supralabral, and labral setae: two, four, and 18–22, respectively. Antennae 1.4–1.5 × as long as head in males and 1.1–1.2 × in females; antennomere 2 > 3 > 4 ~ 5 > 6. Promentum of gnathochilarium separating lamellae linguales in their proximal 2/5 or so, each of the latter with three or four setae in a longitudinal row. Collum mostly smooth, with just two or three shallow grooves at posterolateral corners.

Body rings slightly vaulted. Prozonae with very short and shallow, mostly parallel, longitudinal striae. Metazonae not very deeply, but densely striate, n*_Schub_* = 10 or 11; setae apparently mostly abraded, ca. 1/2–2/3 of metazonal length. Ozopores relatively large, placed right in pro-metazonal suture in more anterior body rings, and ca. 1× their diameter in more posterior ones; sutures not sinuous in front of ozopores. Tarsus of mid-body legs ca. 1.2 × as long as tibia and 3 × as long as apical claw.

***Telson*** (Fig. 4C): Epiproct moderately long in males, almost reaching the level of the longest paraproctal setae, considerably shorter and stouter in females, ending with a short and blunt hyaline tip turned slightly dorsad. Hypoproct roundly trapezoid, barely protruding behind rear contour of paraprocts in males, broadly rounded in females, completely concealed under paraprocts in females. Paraprocts densely covered with relatively long setae; without distinct rows of shorter setae along caudal margins.

**Figure 5.**
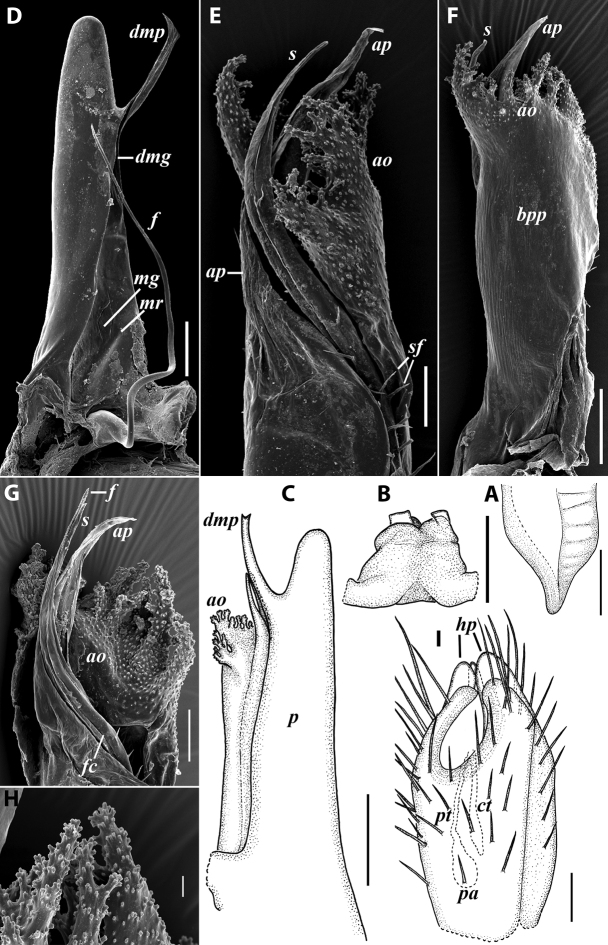
*Colchiobrachyiulusmontanus* sp. nov., paratypes (ZMUM) **A–C** ♂ from near Arkhyz, Russia **A** left flange of pleurotergum 7, ventro-lateral view **B** penis, caudal view **C** right gonopods, oral view **D–H** ♂ from Mount Mussa-Achitara, Russia **D** right promere, caudal view **E** distal part of left opisthomere, mesal-oral view **F** right opisthomere, caudal view **G** apical part of left opisthomere, with the flagellum remained in its channel, oral view **H** branches of apical outgrowth of basoposterior process **I** ♀ from Mount Mussa-Achitara, Russia left vulva, caudal view. Scale bars: 0.2 mm (**A–C, I**), 0.1 mm (**D, F**), 0.05 mm (**E, G**), 0.01 mm (**H**). Abbreviations: ***ap*** anterior process, ***ao*** apical outgrowth, ***bpp*** basoposterior process, ***ct*** central tube, ***dmg*** distomesal groove, ***dmp*** distomesal process, ***f*** flagellum, ***fc*** flagellum channel, ***hp*** hyaline protrusions, ***mg*** median groove, ***mr*** median ridge, ***p*** promere, ***pa*** posterior ampulla, ***pt*** posterior tube, ***s*** solenomere, ***sf*** spiniform filaments.

***Male sexual characters*:** Mandibular stipites considerably expanded, protruding mostly ventrad, forming no distinct corner. Leg pair 1 compact, rounded, parallel hooks. Walking legs with crested adhesive pads, both tibial and postfemoral ones gradually reduced posteriad, but still visible until caudalmost pairs. Pleurotergum 7 ventrally forming elongated, narrowly rounded lobes (Fig. 5A) originating from the zone around pro-metazonal suture, protruding mostly ventro-mesad behind gonopods. Penis (Fig. 5B) short and stout, with broad and rounded apical lobes ending up in short and blunt terminal lamellae directed distad.

***Gonopods*** (5C, D–H): Promere (Fig. 5D, p in Fig. 5C) considerably higher than opisthomere, slender, gradually tapering towards a narrowly rounded apex; mesal margin distally bearing a long and fine process with a pointed tip turned mesad; caudal surface with a short and very weakly pronounced median ridge, a broad and shallow median groove, and a deep and narrow distomesal groove, the latter in situ enveloping the lateral side of solenomeral apex, ending up in an even narrower channel running through distomesal process; flagellum thin, slightly longer than height of promere, with a micro-dentate apical part. Opisthomere (Fig. 5C, E–H) relatively slender; basoposterior process shaped as a broad and flattened lobe running parallel to CBO, mostly fused to it, ending in a multibranched, coral-like, papillose, freely protruding, apical outgrowth; anterior process well-developed, long and tapering, twisted at > 90° around solenomere, partly enveloping the lateral and posterior sides of the latter, both being subequal in height; mesal side with a small lobe (presumably gonocoxal gland), and a rather shallow anteromesal sinus frontal to the gland; two sparse rows of very long, erect, spiniform filaments running along basomedial section of flagellum channel; solenomere very fine and slender, unipartite, sigmoid, without specialised structures apically.

***Female sexual characters*:** Leg pairs 1 (significantly) and 2 (slightly) shorter and thicker than following legs. Vulva (Fig. 5I) rather elongated, subcylindrical, mostly symmetrical (lateral valve somewhat broader than mesal one), with an obtuse postero-apical margin; operculum as high as bursa; both bursa and operculum apically with rather large hyaline protrusions; setation moderately dense throughout. Receptaculum seminis composed of a relatively narrow and moderately long, digitiform, central tube, and a very thin posterior tube, the latter somewhat bent on its way to a medium-sized, ovoid, posterior ampulla.

#### General distribution.

NWGC.

#### Remarks.

Apart from body size and gonopodal structural details, *C.montanus* sp. nov. differs from *C.dioscoriadis* comb. nov. also by the shape of the vulval receptaculum (cf. fig. 182 in Vagalinski and Lazányi 2018).

Two of the four paratype females have chunks of tar-like substance attached to the vulvae, like those observed in *C.dioscoriadis* comb. nov.

### Genus Cyphobrachyiulus

Taxon classificationAnimaliaJulidaJulidae

Verhoeff, 1900

86FDE090-0480-5553-98D6-EACDC9B04667

#### Updated diagnosis.

A genus of Brachyiulini differing from contribal genera by the following combination of characters: promeres positioned completely anteriorly in relation to opisthomeres; opisthomere lacking a basoposterior process or this represented by only a small remnant, also lacking an anterior process.

### Subgenus Diaxylus

Taxon classificationAnimaliaJulidaJulidae

 Attems, 1940

1F37B7AA-F1A6-5B10-8F5E-439F59111710

#### Updated diagnosis.

A subgenus of the genus *Cyphobrachyiulus* differing from other subgenera by the following combination of characters: opisthomere possessing an apicoposterior process and lacking a lateral process, or the latter represented by a weakly developed lobe; promere ending in a broad margin, rather than in a distinct apex, protruding in a conspicuous mesal tip; vulval operculum subequal to bursa; male coxae 2 not enlarged.

### Cyphobrachyiulus (Diaxylus) litoreus

Taxon classificationAnimaliaJulidaJulidae

(Lignau, 1903)

09EF9908-8F85-526C-8CD8-8056B0E2E079

Julus litoreus Lignau, 1903: 137–138, figs 55–58.
Chromatoiulus (Chromatoiulus) litoreus : Attems 1940: 306.
Megaphyllum litoreum : Talikadze 1984: 143.
Cyphobrachyiulus (Diaxylus) litoreus : Vagalinski and Lazányi 2018: 58.

#### Material examined.

1 ♂ (ZMUM), **Georgia**: Kutaisi District, 8 km E of Orpiri, environs of Cave Tsutskhvati, deciduous forest, rock, litter, 24.X.1981, SIG leg.

#### Previous records from the Caucasus.

**Russia**: Adler (type locality).

#### General distribution.

WECA?

#### Remarks.

The above represents the first record of this species since its original description, suggesting a broader distribution within the western parts of the Greater Caucasus. Nevertheless, considering the high collecting activity that has taken place over the years in this particular area of the Caucasus, *C.litoreus* seems to be a rare species. The species is new to the fauna of Georgia.

### Subgenus Grusiniulus

Taxon classificationAnimaliaJulidaJulidae

Lohmander, 1936 stat. nov.

7D5B2B66-B40D-5804-B005-64B5F5C73DBC

#### Updated diagnosis.

A subgenus of the genus *Cyphobrachyiulus* differing from other subgenera by the following combination of characters: promere significantly shorter than opisthomere; opisthomere possessing an apicoposterior process and lacking a lateral process or lobe; male coxae 2 not enlarged.

#### Comment.

Apart from the unusually short promere, the gonopods and vulva in *Grusiniulus* match the diagnosis of the genus *Cyphobrachyiulus*. Consequently, the former taxon is downgraded to a subgenus of the latter genus. Examination of the gonopods of the sole species, Cyphobrachyiulus (Grusiniulus) redikorzevi (Lohmander, 1936), shows that the opisthomeral ‘horizontally protruding protecting branch directed mesocaudad’ [translated from Lohmander (1936)] is not part of the solenomere, as interpreted by Vagalinski and Lazányi (2018), but is homologous to the apicoposterior process present in some species of *Cyphobrachyiulus*. Grusiniulus is particularly similar to the subgenus Diaxylus in having a short thumb-like apicoposterior process of the opisthomere that protrudes perpendicularly to CBO, and a subquadrangular promere (i.e. with a broad rather than tapering apex, and with mostly parallel mesal and lateral margins). However, the very short promere in C. (G.) redikorzevi seems to provide sufficiently solid grounds for keeping the name *Grusiniulus* in use.

### Cyphobrachyiulus (Grusiniulus) redikorzevi

Taxon classificationAnimaliaJulidaJulidae

(Lohmander, 1936) comb. nov.

93A67E58-7197-5DCF-A6D1-E0F9137738EF

Fig. 6

Grusiniulus redikorzevi Lohmander, 1936: 148–152, figs 126–130.
Grusiniulus redikorzevi : Kobakhidze, 1964: 191; 1965: 394; Vagalinski and Lazányi 2018: 78; Kokhia and Golovatch 2018: 40; 2020: 205.

#### Material examined.

1 ♂, 2 ♀♀ (ZIN), **Armenia**: Lake Sevan, Elenovka [Sevan], 16.VI.1927, A. Schelkovnikov leg.

#### Descriptive notes.

20–25 labral setae. Promentum of gnathochilarium rather large, separating both lamellae linguales nearly halfway, each with four setae in a longitudinal row. Collum remarkably long, with five or six well discernible striae at posterolateral corner. Paraprocts very densely setose, without rows of shorter setae at caudal margins. Male leg pair 1 mostly parallel, slightly converging hooks, somewhat more slender compared to the usual brachyiulinine pattern. Male walking legs each with a crested adhesive pad on tibia and postfemur, tibial one very large, strongly protruding, covering the proximal half of tarsus; both pads gradually reduced towards telson, but still visible in last leg pairs; legs 3–8 additionally with a pad rudiment on femur; tarsus and tibia, on midbody legs subequal in length and 3.5–4 × as long as apical claw.

**Figure 6.**
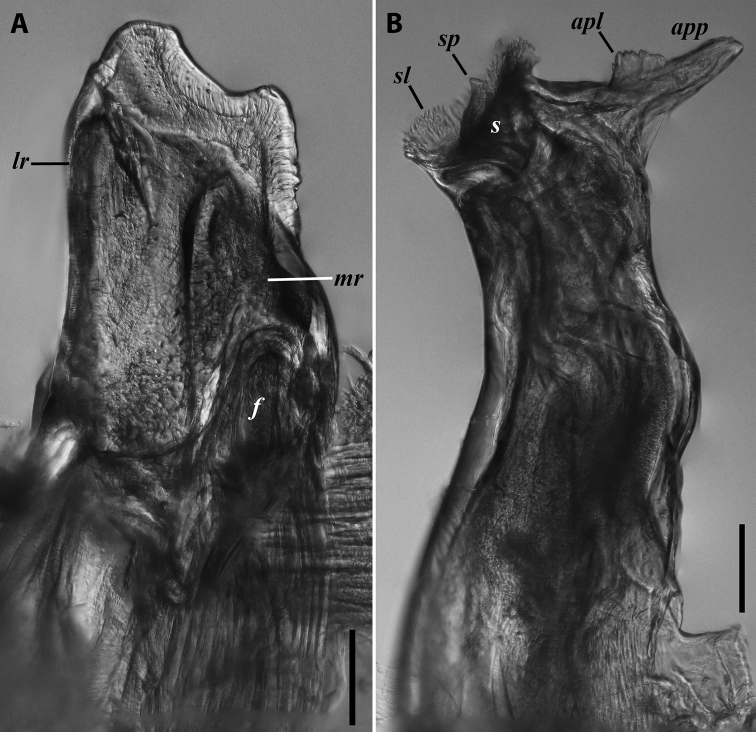
Cyphobrachyiulus (Grusiniulus) redikorzevi (Lohmander, 1936), comb. nov., gonopods of ♂ from Sevan, Armenia (ZMUM) **A** right promere, caudal view **B** right opisthomere, lateral view. Scale bars: 0.1 mm. Abbreviations: ***apl*** lamellae of apicoposterior process, ***app*** apicoposterior process, ***f*** flagellum, ***lr*** lateral ridge, ***mr*** median ridge, ***s*** solenomere, ***sl*** lamella of solenomere, ***sp*** central process of solenomere.

Penis very small, roughly quadrangular, slightly broader than long, with barely discernible apical lobes ending with short, blunt, terminal lamellae directed distad. Gonopods (Fig. 6): Promere (Fig. 6A) ~ 1/2 the height of opisthomere, roughly quadrangular, with parallel side margins and an incised apical margin; caudal surface with a median ridge present as a massive lobe occupying most of promere’s mesal half, basally with a groove for the flagellum to hinge into, and with a lateral ridge somewhat higher and narrower than the median ridge. Opisthomere (Fig. 6B) elongated; basoposterior process vestigial, represented by a faint hump at mid-height; apicoposterior process almost straight, slightly tapering distad, ending bluntly, directed mesocaudad, with short serrate lamellae ventrally at base; solenomere very short and broad, apically forming a deep hollow surrounded by a lamella, the latter protruding higher on lateral side, with a serrate edge; a short, central, linguiform process.

#### Previous records from the Caucasus.

**Georgia**: Akhalkalaki and Borjomi (original localities).

#### General distribution.

CECA-LECA.

#### Remarks.

The species is new to the fauna of Armenia. The examined individuals have sparse and short metazonal setae, and very densely setose paraprocts, while the syntypes, according to the original description, completely lack metazonal setae and show only a sparse pilosity on the paraprocts; the type specimens are also somewhat larger. Apart from these differences, all other characters studied completely match Lohmander’s (1936) observations.

### Genus Iraniulus

Taxon classificationAnimaliaJulidaJulidae

Attems, 1951

2AEB39A4-316F-5719-9DAF-E2802D43FC35

#### Updated diagnosis.

A genus of the tribe Brachyiulini differing from contribal genera by the unique, complex structure of the solenomere which consists of a basomesal process terminating the flagellum and seminal channels, a basocaudal process, and a branched apical part; as well as by the following combination of other characters: promeres positioned completely anteriorly in relation to opisthomeres; opisthomere with a lamellar basoposterior process ending with a visor-like apical outgrowth protruding mostly caudad, and an anterior process; a lateral process absent or poorly developed, in the shape of a rather weakly pronounced lobe.

#### General description.

- Small to medium-sized Brachyiulini (L (males) = 11–32 mm).
- Ommatidia present.
- Ozopores right on or tightly behind pro-metazonal suture at least on more anterior body rings.
- Epiproct well-developed, markedly long.
- Male hypoproct broadly rounded, without setae.
- Male mandibular stipites considerably expanded, broadly rounded, anterior/anteroventral corner either indistinct or broadly rounded.
- Male walking legs ventrally with two well-developed adhesive pads, one each on postfemur and tibia.
- Penis short and stout, with indiscernible or very short apical lobes, as well as small and rounded terminal lamellae.
- Gonopods:

- In situ protruding from gonopodal sinus only with their apical parts, directed completely ventrad.
- Promere as high as opisthomere, with a broad base, more or less narrowing distad and bearing a slender, tapering, apicomesal process.
- Opisthomere from rather stout to slender; a basoposterior process mostly vestigial, i.e., present as a weakly pronounced vertical ridge running parallel to CBO, ending with a simple, lamellar, apical outgrowth protruding nearly perpendicular to CBO; anterior process fine and tapering; a lateral process absent or represented by a small lobe; a mesomeroidal lobe absent; solenomere complex, consisting of a tapering basomesal process and several other small processes and lobes apically.

- Vulva:

- Subcylindrical, mostly symmetrical.
- Bursa with a distinct, slightly obtuse to subrectangular, postero-apical margin.
- Opening placed right on top of bursa.
- Operculum shorter than, to subequal to, bursa.
- Receptaculum seminis: central tube narrow, digitiform; posterior tube very narrow, more or less folded; posterior ampulla small to medium-sized.

#### Remarks.

Vagalinski and Lazányi (2018) referred to the distocaudal projection of the opisthomere in *I.fagorum* (Attems, 1951), the single known species of the genus at that time, as an apicoposterior process, because of its position and orientation being just like those of the latter process in other Brachyiulini, e.g., in the genera *Enghophyllum* Lazányi & Vagalinski, 2013 or species of *Cyphobrachyiulus* and *Graecoiulus* Vagalinski & Lazányi, 2018. However, SEM micrographs of the gonopods of both *I.fagorum* and *I.tricornis* sp. nov. reveal that what have seemed like an apicoposterior process does not originate immediately from the CBO, but is a continuation of the basoposterior process or its apical outgrowth, as denoted in species of the genera *Omobrachyiulus* and *Byzantorhopalum*. Also, in the aforementioned paper, the process observed disto-anteromesally on the opisthomere in *I.fagorum* (Attems, 1951) was called a meso-anterior process, suggesting homology with the similarly positioned process present in another two brachyiulinine genera, *Brachyiulus*Berlese 1884 and *Graecoiulus*. Again, from the SEM pictures it becomes clear that the flagellum and seminal channels terminate in that process rather than at the very top of the solenomere. Thus, that process in *Iraniulus* must be considered as part of the solenomere, unlike the condition observed in *Brachyiulus* and *Graecoiulus*, in both of which (as in all remaining Brachyiulini) the two channels end up at the solenomere apex.

### Iraniulus fagorum

Taxon classificationAnimaliaJulidaJulidae

(Attems, 1951)

33C3DA18-F2BE-52A9-A8D9-D5125BFEEA8F

Figs 7 , 8

Chromatoiulus (Iraniulus) fagorum Attems, 1951: 421–422, figs 39–41.
Chromatoiulus fagorum : Bababekova 1969: 22; 1996: 90; Rakhmanov 1972: 116; Samedov et al. 1972: 1245; Rasulova and Rakhmanov 1973: 519.
Megaphyllum loeffleri : Golovatch 1983 (lapsus calami): 166.
Megaphyllum fagorum : Enghoff and Moravvej 2005: 66.
Iraniulus fagorum : Vagalinski and Lazányi 2018: 78–80, figs 156–160.

#### Material examined.

**Azerbaijan**: 1 ♀ (SMNG), Lerik District, Hyrcan Nature Reserve, road Lǝnkǝran-Lerik at km 322, 38.7638°N, 48.5819°E, 400 m a.s.l., small valley, forest of *Parrotia* with some *Quercus*, in thick litter, 26.III.2015, D. Antić and H. Reip leg.; 2 ♂♂, 4 ♀♀, 8 juv. (ZMUM), Lankaran, Istisu W of Astara, forest, 29.VII.1974, SIG leg.; 6 ♂♂, 5 ♀♀, 2 juv. (ZMUM), Istisu, ca. 8 km SW of Masallı, *Quercus*, *Acer*, *Carpinus* etc., 80–140 m, in litter, under bark and stones, 19–20.X.1983, SIG leg.; 2 ♂♂ (ZMUM), Yardımlı, Avash, 1200–1500 m, 14–17.VI.1996, SIG leg.; 1 ♂, 2 ♀♀ (ZMUM), Astara, Istisu, W of Astara, 100 m, 2– 6.VI.1996, W. Schawaller leg.; many ♂♂, ♀♀, juv. (ZMUM), ca. 6 km WSW of Astara, *Quercus*, *Acer*, *Carpinus*, etc., 10–30 m, in litter and under bark, 18.III.1983, SIG leg.; 1 ♂, 1 ♀, 2 juv. (ZMUM), Lankaran, Alekseevka (Avrora), 24.VII.1974, SIG leg.

#### Diagnosis.

A species of *Iraniulus* differing from its only known congener, *I.tricornis* sp. nov., by a larger body (males > 25 mm long and higher than 1.7 mm, females > 30 mm long and ca. 2.5 mm high, vs. males < 20 mm long and ca. 1 mm high, females < 25 mm long and ca. 1.5 mm high in *I.tricornis* sp. nov.), by having a proportionately longer, more slender opisthomere, and by certain details of solenomere structure: directed completely distad, with a larger basocaudal process, and with an apical part ending in two short rounded branches of equal size on the mesal side, and one slender sigmoid branch on the lateral side, vs. the same turned somewhat caudad in *I.tricornis* sp. nov., with a proportionately smaller basocaudal process, and with an apical part bearing three sharply pointed branches of equal size.

#### Descriptive notes.

**Figure 7.**
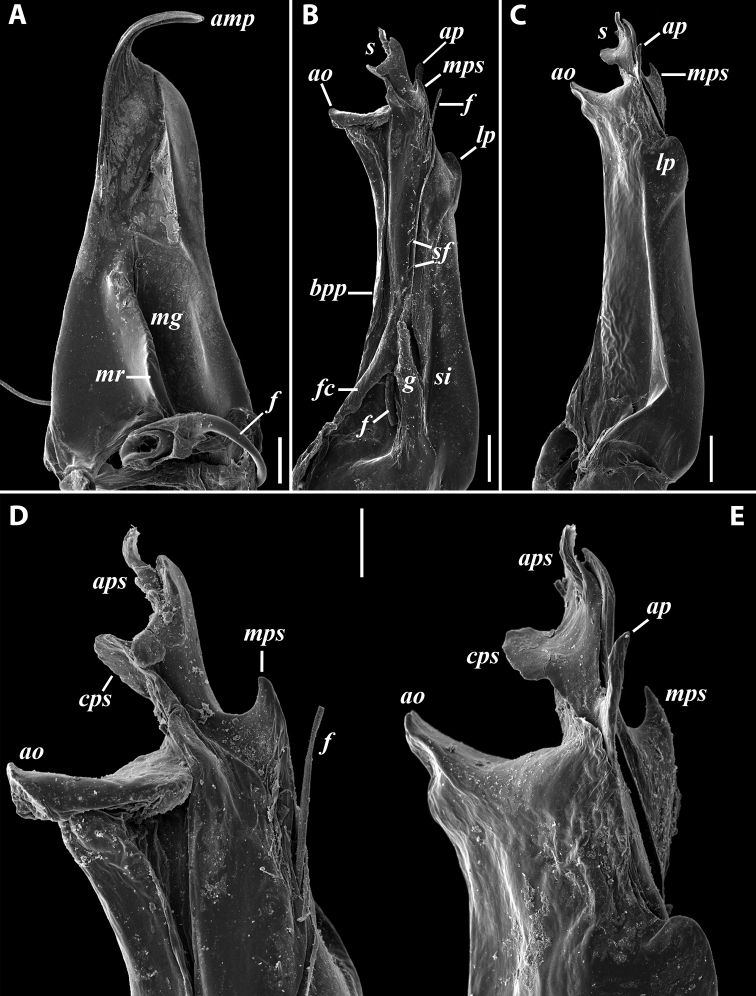
*Iraniulusfagorum* (Attems, 1951), gonopods of ♂ from near Astara, Azerbaijan (ZMUM) **A** left promere, caudal view **B** right opisthomere (with flagellum in its channel), mesal view **C** left opisthomere, oral-lateral view **D** apical part of right opisthomere, mesal view **E** apical part of left opisthomere, oral-lateral view. Scale bars: 0.1 mm (**A–C**), 0.05 mm (**D, E**). Abbreviations: ***amp*** apicomesal process, ***ao*** apical outgrowth of basoposterior process, ***ap*** anterior process, ***aps*** apical part of solenomere, ***bpp*** basoposterior process, ***cps*** basocaudal process of solenomere, ***f*** flagellum, ***fc*** flagellum channel, ***g*** (supposed) gonocoxal gland, ***lp*** lateral lobe, ***mg*** median groove, ***mps*** basomesal process of solenomere, ***mr*** median ridge, ***s*** solenomere, ***sf*** spiniform filaments, ***si*** anteromesal sinus.

**Figure 8.**
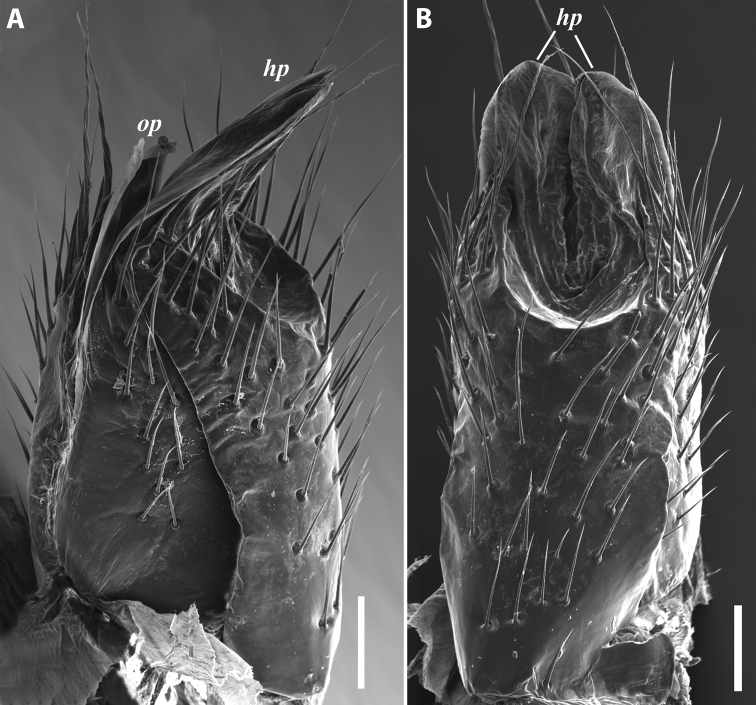
*Iraniulusfagorum* (Attems, 1951), ♀ from E of Lerik, Azerbaijan (SMNG) right vulva **A** caudo-lateral **B** meso-caudal view. Scale bars: 0.1 mm. Abbreviations: ***hp*** hyaline protrusions, ***op*** operculum. Pictures by courtesy of Karin Voigtländer, Hans Reip and Dragan Antić.

***Gonopods*:** Promere (Fig. 7A) as high as opisthomere, broadest at base, significantly narrowing distad; apex narrow; apicomesal process similar to that in *I.tricornis* sp. nov., but longer; flagellum just slightly longer than height of promere. Opisthomere (Fig. 7B–E) relatively slender; anterior process tapering, straight; basoposterior process with its apical outgrowth protruding at nearly 90° to CBO, bent slightly apicad; a small lobe distolaterally; mesal side with a narrow, slender lobe (presumably gonocoxal gland) and a rather narrow anteromesal sinus; with a row of sparse spiniform filaments along flagellum channel; solenomere consisting of a tapering basomesal process, a broadly rounded basocaudal process, and an apical part ending in three minute branches: two rounded mesal ones, and a sigmoid lateral one. ***Vulva*** (Fig. 8) mostly corresponding to the description and drawing given in Vagalinski and Lazányi (2018) except for the opening, which is not large as claimed by these authors, but is in fact minute, hidden between the large bursal hyaline protrusions.

#### General distribution.

Iran, Lahijan (type locality); Azerbaijan, Lenkoran [Lankaran] (Samedov et al. 1972). Apparently, a Hyrcanian endemic.

#### Remarks.

*Iraniulusfagorum* can easily be recognised in the field by both its greyish green colouration and its specific, strong odour (Dragan Antić, pers. comm.). The latter is the result of the large proportion of p-cresol (> 90%) in its defensive secretion. This compound is known to be produced by only a few species of Julidae, while it is typical of the order Callipodida (Bodner et al. 2016). Interestingly, p-cresol may be a major compound in the secretions of another two julids inhabiting the Caucasus, namely, *Pachyiuluskrivolutskyi* Golovatch, 1977 from Colchis and *Syrioiuluscontinentalis* (Attems, 1903) from Hyrcania, both species showing a similar bright yellow-greyish colouration reminiscent of the green tinges in *I.fagorum* (Evsyukov et al. 2021).

### Iraniulus tricornis

Taxon classificationAnimaliaJulidaJulidae

Vagalinski sp. nov.

D646B418-1C06-5F79-8324-116D94B118CF

http://zoobank.org/B752D188-20CE-4030-A735-845D73F3E7CB

Figs 9 , 10 , 11

#### Material examined

**(all from Georgia, Svanetia): *Holotype***: ♂ (unbroken) (ZMUM), Khumpreri River (left affluent of Enguri), near Dizi, 1.5–2 km before the influx, ca. 1000 m a.s.l., leaf litter, 14.IX.1986, A. Ryvkin leg. ***Paratypes***: 6 ♂♂ (ZMUM) (one in 4 pieces, gonopods prepared for SEM, leg pair 2 and penis dissected; another in head and 2 pieces, gonopods in situ, the rest unbroken), 1 ♂ (NMNHS) (in 2 pieces with dissected gonopods), 1 ♂ (NHMD) (unbroken), 1 ♂ (IBER) (in 2 pieces, gonopods dissected, one flagellum prepared for SEM), 3 ♀♀ (ZMUM) (unbroken), 1 ♀ (NMNHS) 1 ♀ (NHMD), 1 ♀ (IBER) (all unbroken), 2 juv. (unbroken), same collecting data as for holotype; 3 ♂♂ (one in head to ring 3, ring 4–6, pleurotergum 7, and rest of body, gonopods and leg pair 2 dissected; the others unbroken), 2 adult ♀♀ (one in head to ring 3 and rest of body, vulvae dissected; the others unbroken), 5 juv. ♀♀ (unbroken) (all in ZMUM), ca. 50 km west of Mestia, west of Dizi, ca. 800 m a.s.l., by a waterfall, in leaf litter, 19.IX.1986, A. Ryvkin leg.; 2 ♂♂ (one in head to ring 6 and rest of body, gonopods dissected; the other unbroken), 3 juv. (unbroken) (all in ZMUM), Mestia, 1500 m, *Betula* and *Rhododendron* on moraine, litter and under stones, 5–16.IX.1986, SIG leg.; 1 ♂ (in head to ring 6 and rest of body), 2 ♀♀, 1 juv. ♂, 2 juv. ♀♀ (all in ZMUM), same locality as for holotype, 9.IX.1986, A. Ryvkin leg.; 6 ♂♂ (one in 3 pieces, gonopods dissected, the others unbroken), 8 ♀♀ (two in 2 pieces, the rest unbroken), 1 juv. ♂, 4 juv. ♀♀ (all in ZMUM), Mestia, 1500 m a.s.l., litter and under stones, 22.X.1979, SIG leg.

#### Diagnosis.

A species of *Iraniulus* differing from its only known congener, *I.fagorum*, by a smaller body: males < 20 mm long and ca. 1 mm high, females < 25 mm long and ca. 1.5 mm high, vs. males > 25 mm long and higher than 1.7 mm, females > 30 mm long and ca. 2.5 mm high in *I.fagorum*), by having a proportionately shorter and stouter opisthomere, and by details of the solenomere: turned somewhat caudad, with a proportionately smaller basocaudal process, and with an apical part bearing three sharply pointed branches of equal size, vs. the same directed completely distad in *I.fagorum*, with a larger basocaudal process, and with an apical part ending in two short, rounded branches of equal size on the mesal side and one slender sigmoid branch on the lateral side.

#### Name.

Meaning three-horned in Latin, referring to the apical part of the solenomere which consists of three pointed branches. Adjective.

#### Description.

***Measurements*:** holotype ♂ in S IX, 43+2+T, L = 16 mm, H = 1.05 mm; paratype ♂♂ in S VII–X, 38–43+2–3+T, L = 11–13 mm, H = 0.85–1.1 mm; paratype ♀♀ in S VII–X, 38–45+1–2+T, H = 1.2–1.6 mm, L = 14–21 mm.

**Figure 9.**
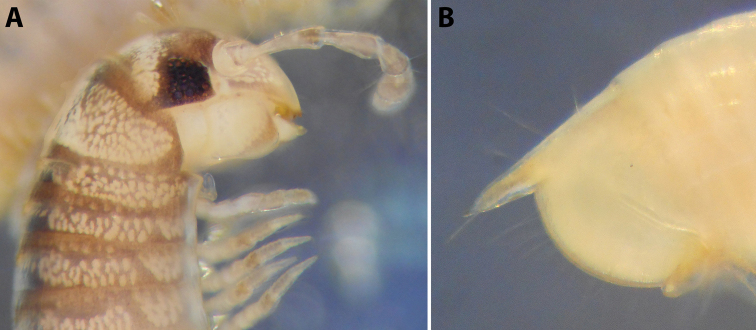
*Iraniulustricornis* sp. nov. (ZMUM) **A** ♂ holotype, head and body rings 1–4, lateral, slightly dorsal view **B** ♂ paratype from near Dizi, Georgia (ZMUM), telson, lateral view. Not to scale.

***Colouration*:** (after > 30 years in alcohol) (Fig. 9): mostly brown-beige; head with the usual colour pattern; antennae light brown, collum brownish, margins dark brown; prozonae dorsally with a broad, dark brown, transverse stripe next to suture, and with irregular dark brown spots just before ozopores; metazonae ochre-brown; dorsum with a continuous, blackish, axial line; legs light brown; telson brown-grey, caudal parts of paraprocts lighter.

***External structures*:** Eye patches in adults consisting of 20–38 ommatidia arranged in hardly traceable vertical rows. Vertigial, supralabral, and labral setae: two, four, and 17–20, respectively. Antennae 1.5–1.6 × as long as head in males and ca. 1.4 × in females; antennomere 2 > 5 ≥ 3 ~ 4 > 6. Gnathochilarium with promentum separating both lamellae linguales in their basal 1/3–2/5, each with three setae in a longitudinal row. Collum mostly smooth, with only 2–3 short striae near posterolateral corners.

Body rings slightly vaulted. Prozonae with very short, shallow, longitudinal striae in posterior parts. Metazonae moderately deeply striated, n*_Schub_* = 7 or 8 in males and 9 or 10 in females; metazonal setae from 2/5 (in more anterior rings) to 1/2 (in more posterior rings) of metazonal length. Ozopores placed right on pro-metazonal suture in first several rings, gradually taking a more posterior position to ~ 1.5 their diameter in caudalmost rings; sutures sinuous in front of ozopores in some rings. Tarsus of mid-body legs slightly shorter than tibia and ca. 3 × as long as apical claw.

***Telson*** (Fig. 9B): Epiproct very long, straight, ending in a fine, tapering, hyaline tip surpassing the longest paraproctal setae in both sexes; with several long setae. Hypoproct broadly rounded in both sexes, somewhat protruding behind rear margin of paraprocts in males, tightly fitting under their ventral side in females; without setae. Paraprocts covered with sparse long setae, but without distinct rows of shorter setae along caudal margins.

**Figure 10.**
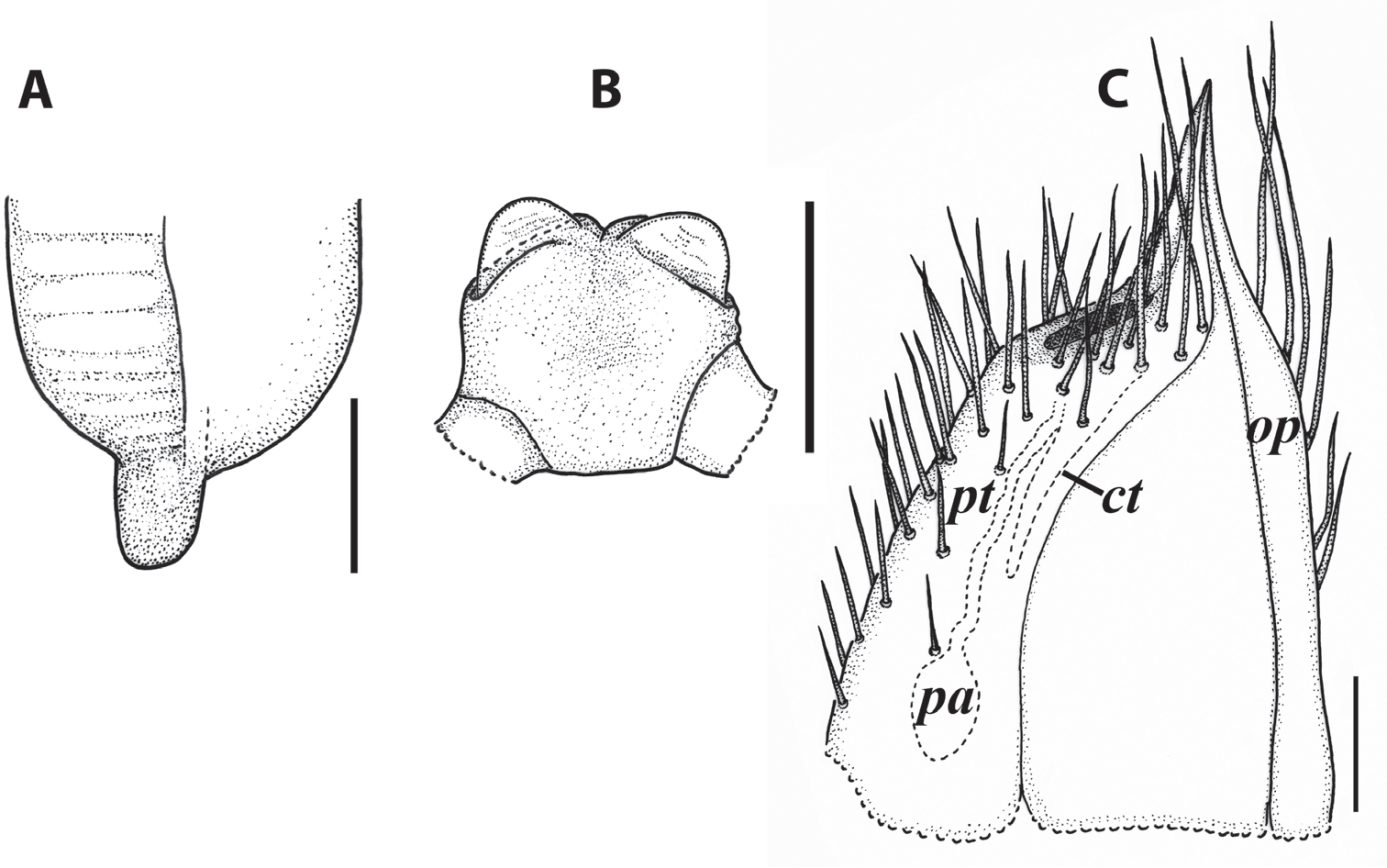
*Iraniulustricornis* sp. nov., ♂ (**A, B**) and ♀ (**C**) paratypes from near Dizi, Georgia (ZMUM) **A** right flange of pleurotergum 7, ventro-lateral view **B** penis, caudal view **C** left vulva, caudo-lateral view. Scale bars: 0.2 mm (**A, B**), 0.1 mm (**C**). Abbreviations: ***ct*** central tube, ***op*** operculum, ***pa*** posterior ampulla, ***pt*** posterior tube.

***Male sexual characters*:** Mandibular stipites (in Fig. 9A) moderately expanded, forming a broadly rounded antero-ventral corner. Leg pair 1 compact and set parallel to hooks turned slightly mesad. Walking legs with crested adhesive pads, both tibial and prefemoral ones reduced towards telson, but still discernible in caudalmost legs; femora without modifications. Pleurotergum 7 ventrally forming relatively small shovel-like lobes (Fig. 10A) originating mostly from metazona, directed largely ventrad. Penis (Fig. 10B) stout, compact, nearly as broad as long, without differentiated apical lobes, but with broad and rounded terminal lamellae directed ventrad.

**Figure 11.**
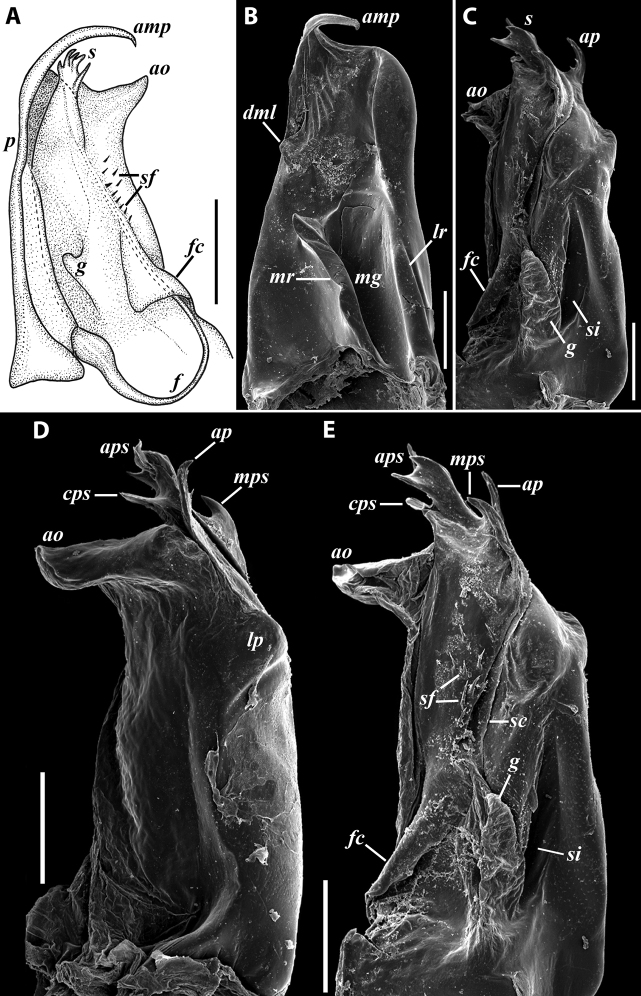
*Iraniulustricornis* sp. nov., gonopods of ♂ paratype from near Dizi (ZMUM) **A** left gonopods, mesal view **B** left promere, caudal view **C** right opisthomere, mesal, slightly oral view **D** left opisthomere, lateral view **E** right opisthomere, mesal view. Scale bars: 0.2 mm (**A**), 0.1 mm (**B–E**). Abbreviations: ***amp*** apicomesal process, ***ao*** apical outgrowth of basoposterior process, ***ap*** anterior process, ***aps*** apical part of solenomere, ***cps*** basocaudal process of solenomere, ***dml*** distomesal process, ***f*** flagellum, ***fc*** flagellum channel, ***g*** (supposed) gonocoxal gland, ***lp*** lateral lobe, ***lr*** lateral ridge, ***mg*** median groove, ***mps*** basomesal process of solenomere, ***mr*** median ridge, ***s*** solenomere, ***sc*** seminal channel, ***sf*** spiniform filaments, ***si*** anteromesal sinus.

***Gonopods*** (Fig. 11): Promere (Fig. 11B, p in Fig. 11A) as high as opisthomere, broadest at base, somewhat narrowed distad; mesal and lateral margins gently sigmoid; apex laterally broadly rounded, mesally bearing a horn-like apicomesal process bent caudolaterad; caudal surface with a very strongly developed median ridge, a somewhat less strongly protruding lateral ridge running all the way to the top, and a relatively deep and broad median groove; a small, rounded, distomesal lobe directed mesad; flagellum somewhat longer than height of promere. Opisthomere (Fig. 11A, C–E) relatively thick and stout; anterior process fine and tapering, bent mesocaudad; apical outgrowth of basoposterior process well-developed, protruding at nearly 90° to CBO; a faint lobe distolaterally; mesal side with a large membranous lobe, probably gonocoxal gland, at the base of seminal channel, and a deep and narrow anteromesal sinus just frontally to the membranous lobe; with a group of spiniform filaments in distal section of flagellum channel; solenomere consisting of a tapering basomesal process bent caudad and terminating both flagellum and seminal channels, a fine basocaudal process directed meso-caudad, and an apical part ending with three more or less equally sized and shaped pointed branches.

***Female sexual characters*:** Leg pairs 1 and 2 somewhat thicker and shorter than following legs. Vulva (Fig. 10C) roughly cylindrical, strongly transversely compressed; bursa with a well-pronounced, slightly obtuse, postero-apical margin; opening small, positioned at the very top of bursa; operculum subequal to bursa, both bursa and operculum apically bearing large hyaline protrusions; setation scattered over vulval surface. Receptaculum seminis consisting of a narrow, finger-shaped, central tube, and a very narrow, long, somewhat folded, posterior tube leading to an ovoid posterior ampulla.

#### General distribution.

SWGC.

### Genus Megaphyllum

Taxon classificationAnimaliaJulidaJulidae

Verhoeff, 1894

3DFDBBAD-A921-51BF-B3E0-22CEC1CC7CE7

#### Updated diagnosis.

A genus of Brachyiulini differing from contribal genera by the following combination of characters: promeres positioned slightly to considerably laterally (up to ca. 45° in species of *Megaphyllums. str.*) rather than completely anteriorly in relation to opisthomeres; promere visibly broader than opisthomere, with a well-developed median groove partly enveloping the opisthomere; opisthomere possessing a well-developed basoposterior process, clearly detached from CBO in at least ~ its distal 1/3, a differently developed, sometimes vestigial anterior process, and lacking lateral process.

### Subgenus Megaphyllum

Taxon classificationAnimaliaJulidaJulidae

Verhoeff, 1894

9C7D4F57-AD6A-5A4D-AA84-26540B575A5C

#### Updated diagnosis.

A subgenus of the genus *Megaphyllum* differing from other subgenera and certain isolated congeners by the following combination of characters: promeres positioned considerably laterally (ca. 45° in most species) rather than completely anteriorly in relation to opisthomeres; promere considerably broader than opisthomere, with a deep median groove tightly enveloping the opisthomere anterolaterally; opisthomere with a small or vestigial anterior process, solenomere composed of two processes, an anterior and a posterior one, the former positioned at the end of the flagellum channel; vulva with an apically or subapically positioned opening, operculum shorter than, or rarely subequal to, bursa; hypoproct trapezoidal or broadly rounded.

### Megaphyllum (Megaphyllum) hercul

Taxon classificationAnimaliaJulidaJulidae

es (Verhoeff, 1901)

C8EA9193-3E9F-516D-9232-0C6E98964E73

Brachyiulus (Chromatoiulus) unilineatus hercules Verhoeff, 1901: 97–98, figs 18–20.
Brachyiulus unilineatus hercules : Lignau 1903: 127, 55.
Chromatoiulus unilineatus hercules : Lang 1959: 1791.
Chromatoiulus unilineatus hercules : Kobakhidze 1965: 394.
Megaphyllum hercules : Lazányi and Vagalinski 2013: 82–84, fig. 15a-g; Kokhia and Golovatch 2018: 40; 2020: 206.

#### Diagnosis.

Differs from its only congener known from the Caucasus, M.(s. str.)spathulatum (Lohmander, 1936), mainly by the colour pattern: body uniformly dark with an orange to dark red mid-dorsal line, vs. body with a light yellow to ochre dorsum divided by a black axial line in *M.spathulatum*; and by the promere having mostly parallel side margins, ending with a flat apex, vs. the same significantly tapering all the way to a narrowly rounded apex in *M.spathulatum*.

#### Records from the Caucasus.

**Russia**: Environs of Novorossiysk (Lignau 1903); **Georgia**: AR Abkhazia, Pitsunda (Issajev 1911).

#### General distribution.

Central and southwestern parts of the Balkan Peninsula, northwestern Caucasus.

#### Remarks.

Apart from the records from the Caucasus region, Lignau (1903) and Issajev (1911) both mentioned that *M.hercules* was rather common across the Crimean Peninsula, and although neither of them documented those reports with gonopod drawings, the very characteristic habitus of this species alone makes misidentification very unlikely: it has a dark grey to blackish body with an orange to dark red axial line, while the other two congeners occurring at the northeastern Black Sea coast, *M.spathulatum* (Lohmander, 1936) and *M.tauricum* (Attems 1907), are both characterised by a black axial line on a light yellow to ochre (*spathulatum*) or brown-grey (*tauricum*) dorsum. Also bearing in mind the disjunct Balkan-Caucasian distribution of *Byzantorhopalumrossicum* (see Vagalinski and Lázanyi 2018), as well as the Crimean endemic *M.tauricum*, a very close sibling of the Balkan *M.rhodopinum* (Verhoeff, 1928), *M.hercules* may be another example of this pattern, the lack of more recent samples from the Caucasus being due to its rarity in the region.

### Megaphyllum (Megaphyllum) spathulatum

Taxon classificationAnimaliaJulidaJulidae

(Lohmander, 1936)

37FB26D6-CA34-5F2E-B479-DF84D78FF828

Chromatoiulus (Chromatoiulus) spathulatus Lohmander, 1936: 104–109, figs 80–84.
Chromatoiulus (Chromatoiulus) spathulatus : Attems 1940: 306.
Chromatoiulus spathulus (sic!): Lang 1959: 1791.
Megaphyllum spathulatum : Golovatch 1990b: 364; 1992: 381; Lazányi and Vagalinski 2013: 87, fig. 18b–d; Kokhia and Golovatch 2018: 41; 2020: 206.

#### Material examined

**(SMNG): Russia**: RU12-86, 1 ♂, 1 ♀, Republic of Adygea, Kamennomostkiy, northern end of Dakhovskaya Canyon near quarry, limestone cliffs, 44.2844°N, 40.1778°E, 430 m a.s.l., 24.VIII.2012, F. Walther leg.; RU12-051, 3 ♂♂, 1 juv., Dakhovskaya, limestone cliffs NW of, 44.2439°N, 40.1758°E, 800 m a.s.l., 13.VIII.2012, F. Walther leg.; 33579, 1 ♂, Republic of Adygea, Mount Shibaba, 21.V.2004, K. Voigtländer leg.

#### Diagnosis.

Differs from its only congener known from the Caucasus, *M.* (*s. str.*) *hercules*, mainly by the colour pattern: body with a light yellow to ochre dorsum divided by a black axial line, vs. body uniformly dark with an orange to dark red mid-dorsal line in *M.hercules*; and by the promere significantly tapering all the way to a narrowly rounded apex, vs. the same having mostly parallel side margins, ending with a flat apex in *M.hercules*.

#### Previous records from the Caucasus.

Western Caucasus (unspecified type locality) (Lohmander 1936).

#### General distribution.

WCIS?

#### Remark.

This species seems to be a narrow local endemic of the northwestern foothills of the Caucasus Major, showing preferences for limestone terrain.

### Genus Omobrachyiulus

Taxon classificationAnimaliaJulidaJulidae

Lohmander, 1936

3FE2E90A-D4DF-5C24-A222-AD83804F2FFB

#### Updated diagnosis.

A genus of Brachyiulini differing from other contribal genera by the following combination of characters: promeres positioned completely anteriorly in relation to opisthomeres; opisthomere with a mesomeroidal lobe on anterior surface, and with a basoposterior process either completely absent (rarely) or present as a vertical lobe or ridge, with only apical part freely protruding; sometimes with a faint lateral lobe, but never with a well-developed lateral process; solenomere well differentiated, mostly slender.

#### Updated general description.

- Small to medium-sized Brachyiulini (L (males) = 10–33 mm).
- Ommatidia present.
- Ozopores set tightly behind pro-metazonal suture at least on more anterior body rings.
- Epiproct present, varying in length.
- Male hypoproct either broad, dentate at margin and trapezoidal or semi-circular/semi-elliptic.
- Male mandibular stipites moderately to considerably expanded, forming a distinct anterior/anteroventral corner.
- Male pleurotergum 7 significantly bulging only in some species.
- Male walking legs ventrally with two well-developed adhesive pads, one each on postfemur and tibia, postfemoral pads rarely lacking in leg pair 2.
- Penis short and stout, usually considerably compressed anterocaudally, with short or indistinct apical lobes.
- Gonopods:

- In situ: promeres slightly to considerably protruding outside gonopodal sinus, directed completely ventrad.
- Promere subequal to or higher than opisthomere, mostly roughly leaf-shaped, caudal side sometimes forming small lobes, but bearing no distinct processes; flagellum usually much longer than height of promere.
- Opisthomere from stout to relatively slender; basoposterior process usually represented by a differently pronounced vertical lobe, apically forming a variously shaped and freely protruding outgrowth; an anterior process usually present, varying in shape and size; a mesomeroidal lobe always present, mostly rounded, varying in size and prominence; solenomere always conspicuously protruding from CBO, usually slender and tubular.

- Vulva:

- Subcylindrical.
- Bursa usually with a distinct, more or less obtuse, postero-apical margin.
- Opening narrow oval or cleft-like, positioned apically.
- Operculum usually somewhat longer than or subequal to, rarely shorter than, bursa.
- Receptaculum seminis: central tube narrow, mostly straight, forming no distinct central ampulla; posterior tube narrow, usually more or less twisted, ending in an ovoid or spherical posterior ampulla.

##### Species groups

Seven species groups can be recognised within the circum-east Mediterranean genus *Omobrachyiulus*: The *caucasicus* group is the most diverse one and includes both Caucasian and Balkan-south Carpathian species; the *hortensis*, *implicitus*, *roseni*, and *sevangensis* groups each comprises three or four Caucasian endemics; *O.mesorientalis* Vagalinski & Golovatch, 2019 from Israel and Lebanon, as well as *O.beroni* (Strasser, 1973) from southern Bulgaria are morphologically the most isolated/disjunct congeners, each representing a group of its own (Vagalinski and Golovatch 2019; Vagalinski et al. in prep., respectively). The former five groups are here characterised by gonopodal and vulval structures which show no clear-cut differences, but rather gradual transitions between the groups. Clear-cut diagnoses of the species groups can therefore not be made. Nevertheless, the recognition of these species assemblages is useful for it allows for certain patterns in the distribution of the Caucasian *Omobrachyiulus* to be drawn (see Maps 2, 3).

##### The *caucasicus* group

**Characterisation.** Both gonopod pairs subequal in height. Promere with a well-developed distal groove. Opisthomere rather slender, with a well-pronounced mesomeroidal lobe, a basoposterior process with a moderately to strongly pronounced proximal part, ending with a variously shaped apical outgrowth, an anterior process in the shape of a small spine or rod of various length, a deep anteromesal sinus, a flagellum channel overgrown with short to moderately long spiniform filaments, and a more or less slender, apically often bipartite solenomere. Vulva with operculum subequal (from slightly lower to slightly higher) to bursa.

**Included species.**

*O.adsharicus* (Lohmander, 1936)

*O.caucasicus* (Karsch, 1881), comb. nov. (= *Brachyiulusbrachyurus* Attems, 1899, the type species of the genus)

*O.curvocaudatus* (Lignau, 1903)

*O.divaricatus* (Lohmander, 1936)

*O.geniculatus* (Lohmander, 1928)

*O.macrourus* (Lohmander, 1928)

*O.platyurus* (Latzel, 1884) (South Carpathians, Romania; Banat, Romania/Serbia)

*O.strasseri* Vagalinski & Lazányi, 2018 (Andros, Greece)

*O.unugulis* Vagalinski, sp. nov.

### Omobrachyiulus adsharicus

Taxon classificationAnimaliaJulidaJulidae

(Lohmander, 1936)

3CE8E8A8-686E-5BE1-AB5C-1625F97C3E0F

Chromatoiulus (Omobrachyiulus) adsharicus Lohmander, 1936: 123–126, figs 99–101.
Chromatoiulus (Omobrachyiulus) adsharicus : Lang 1959: 1791.
Chromatoiulus adsharicus : Kobakhidze 1964: 191; 1965: 393.
Megaphyllum adsharicum : Talikadze 1984: 143.
Omobrachyiulus adsharicus : Vagalinski and Lazányi 2018: 94; Kokhia and Golovatch 2018: 41; 2020: 206.

#### Material examined.

**Georgia**: AR Ajara: 1 ♂ (SMNG), Machakhlispiri, 2.7 km upstream of Acharistskali, 41.5278°N, 41.7211°E, 29.IX.2011, F. Walther leg.; 6 ♂♂, 8 ♀♀, 4 juv. (ZMUM), Keda Municipality, Makhuntseti, *Platanus* grove, in leaf litter, 9.X.1981, SIG leg.

#### Diagnosis.

A species of *Omobrachyiulus* differring from congeners mainly by the solenomere being apically bifurcate into two pointed branches of nearly equal size and shape, in combination with the complete absence of an anterior process on the opisthomere.

#### Previous records from the Caucasus.

**Georgia**, AR Ajara, near mouths of Chorokhi and Acharistskali rivers (type locality).

#### General distribution.

LECA.

### Omobrachyiulus caucasicus

Taxon classificationAnimaliaJulidaJulidae

(Karsch, 1881) comb. nov.

A919AB30-AC4C-57A9-948B-D8175C596AE3

Fig. 12

Julus caucasicus Karsch, 1881: 20.
Brachiulus (sic!) brachyurus Attems, 1899: 326–328, figs 72–75, syn. nov.
Chromatoiulus brachyurus : Rasulova and Rakhmanov 1973: 519.
Chromatoiulus (Omobrachyiulus) brachyurus : Lohmander 1936: 114–120, figs 86, 87, 93–95, 102.
Chromatoiulus (Omobrachyiulus) brachyurus dagestanus Lohmander, 1936: 121–123, figs 96–98; Lang 1959: 1791.
Chromatoiulus brachyurus brachyurus : Kobakhidze 1964: 190; 1965: 393, 396; Samedov et al. 1972: 1245; Rakhmanov 1972: 116; Bababekova 1996: 90.
Chromatoiulus brachyurus dagestanus : Kobakhidze 1964: 191.
Chromatoiulus (Omobrachyiulus) brachyurus brachyurus : Lang 1959: 1791; Lokšina and Golovatch 1979: 385.
Megaphyllum brachyurum brachyurum : Samedov 1991: 195.
Megaphyllum brachyiurum (sic!): Loginova 1993: 151–153.
Megaphyllum caucasicum caucasicum : Kokhia 1999: 232.
Megaphyllum brachyurum : Enghoff and Moravvej 2005: 66; Golovatch and Matyukhin 2011: 116; Zuev 2014: 352, map 4.
Omobrachyiulus brachyurus : Vagalinski and Lazányi 2018: 94–96, figs 184–188; Kokhia and Golovatch 2018: 41; 2020: 206.

#### Material examined.

**Georgia: *Holotype***: juvenile ♀ (by monotypy) (ZMB 748), Borjomi (Karsch 1881). **Armenia**: 1 ♂, 1 ♀, 4 juv. (ZMUM), Byn Kulp(?), 15.VIII.1927, M. Makaryan leg.; 3 ♂♂, 5 ♀♀, 1 juv. (ZMUM), halfway between Alaverdi and Bagratashen, *Carpinus* forest, in litter, 24.V.1987, SIG and K. Eskov leg.; many ♂♂, ♀♀, juv. (ZMUM), Ijevan, deciduous bush, in litter, 16.IV.1983, SIG leg.; 1 ♂, 1 juv. (ZMUM), Odzun, W of Alaverdi, 1500–1550 m, *Quercus*, *Fagus*, *Carpinus* etc. forest, in litter and under stones with ants, 23–24.V.1987, SIG and K. Eskov leg.; 2 ♂♂, 1 ♀, 7 juv. (ZMUM), Stepanavan, 1600–1650, *Quercus*, *Fagus*, *Carpinus* etc. forest, in litter and under bark, 21–22.V.1987, SIG and K. Eskov leg. **Azerbaijan**: 1 ♂, 1 ♀, (SMNG), İsmayıllı District, S of Zərgəran, 40.7310°N, 48.3680°E, 880 m a.s.l., slope with *Corylus*, *Clematis* and some *Prunus* trees, stone heaps overgrown by moss, mainly in thick litter and under stones, 30.III.2015, D. Antić and H. Reip leg.; many ♂♂, ♀♀, juv. (ZMUM), Shamakhi District, Pirkuli, near observatory, 1200–1250 m, *Quercus*, *Acer*, *Taxus* etc. forest, litter, 30.IV.1987, SIG and K. Eskov leg.; 2 ♂♂, 3 ♀♀, 1 juv. (ZMUM), Talysh Mts, Djoni, 1500 m, 28–29.V.1976, V.G. Dolin leg.; 2 ♂♂, 4 ♀♀, 3 juv. (ZMUM), NW of Bash Layisqi, ca. 20 km NNW of Sheki, 1250 m, *Fagus*, *Carpinus*, *Acer* etc. forest, 3.V.1987, in litter, SIG and K. Eskov leg.; 2 ♂♂, 4 ♀♀ (ZMUM), 9 juv., ca. 5 km N of Kutkashen, 1150–1200 m; *Fagus* and *Carpinus* forest, in litter and rotten wood, 2.V.1987, SIG and K. Eskov leg.; ♂, ♀ (ZMUM), Altyagach National Park, 1050–1100 m, mixed broadleaved forest, litter, 20 and 26.IV.1987, SIG and K. Eskov leg.; 1 ♂ (ZMUM), Zagatala (= Zakataly) Nature Reserve, Agkemal, 1800–2100 m, forest, 24– 27.V.1981, SIG and J. Martens leg.; many ♂♂, ♀♀, juv. (ZMUM), Laza, 2200 m, 10.VII.1985, H. Aliyev leg.; 3 ♂♂, 4 ♀♀ (ZMUM), Istisu, ca. 8 km SW of Masally, *Quercus*, *Acer*, *Carpinus*, etc., 80–140 m, litter, under bark and stones, 19–20.X.1983, SIG leg.; many ♂♂, ♀♀, juv. (ZMUM), Azerbaijan, above Agsu, 120 km W of Baku, 900 m, *Quercus*, shrub, 22.V.1981, SIG and J. Martens leg.; **Georgia**: 1 ♂, 1 ♀, Borjomi District, Akhaldaba, 1000 m, Nedzura River Valley, *Picea*, *Carpinus* and *Fagus* forest, in litter, under logs, 12.V.1983, SIG leg.; many ♂♂, ♀♀, juv. (ZMUM), S of Bakuriani Pass, Tskhratskaro, 2100 m, sparse *Betula* forest at timberline, 13.V.1983, SIG leg.; many ♂♂, ♀♀, juv. (ZMUM), Lagodekhi, *Fagus*, *Carpinus* etc. forest, XI.1967, T.S. Perel leg.; many ♂♂, ♀♀, juv. (ZMUM), AR Ajara, 15 km W of Adigeni, *Abies*, *Picea*, *Fagus*, *Acer* forest, 1500–1700 m, litter, logs and under stones, 14–15.V.1983, SIG leg.; many ♂♂, ♀♀, juv. (ZMUM), Mayakovskii District, above Sairme, 1700–2000, 1.V.1985, E. Kvavadze leg.; 3 ♂♂, 5 ♀♀ (ZMUM), Babaneuri Nature Reserve, ENE of Akhmeta, near Babaneuri, 500 m, 4–5.V.1987, SIG and K. Eskov leg.; 2 ♂♂ (ZMUM), Magalakhari Pass, between Akhmeta and Tianeti, *Fagus* and *Carpinus* forest, 1200 m, in litter and under bark, 6.V.1987, SIG and K. Eskov leg.; many ♂♂, ♀♀, juv. (ZMUM), N of Kvareli, 700–750 m, *Fagus*, *Carpinus*, *Quercus* etc. forest, in litter and under bark, 4.V.1987, SIG and K. Eskov; many ♂♂, ♀♀, juv. (ZMUM), Batsaro Nature Reserve, ca. 20 km N of Akhmeta, *Fagus*, *Castanea* etc. forest, 800–850 m, in litter, 5–6.V.1987, SIG and K. Eskov leg.; 1 ♂, 1 ♀ (ZMUM), Mariamjvari Nature Reserve, ENE of Sagarejo, 1150–1250 m, *Fagus*, *Carpinus*, *Acer*, *Pinus* etc. forest, in litter, under bark and stones, 13–14.V.1987, SIG and K. Eskov leg.; 1 ♂, 7 juv. (ZMUM), Saguramo Nature Reserve, NE of Mtskheta, Zedazeni, 1100–1200 m, *Fagus*, *Carpinus*, *Acer* etc. forest, litter and under bark, 20.VII.1987, SIG and K. Eskov leg.; many ♂♂, ♀♀, juv. (ZMUM), Racha, Oni District, N of Utsera, decidous forest near spring, rock, litter, 20.IX.1981, SIG leg.; 2 ♂♂, 2 ♀♀ (ZMUM), 45 km W Mestia, above Nakra, 1700 m, *Abies*, *Picea* etc., forest, in litter and under stones, 3.IX.1986, SIG leg.; many ♂♂, ♀♀, juv. (ZMUM), above Sairme, 1700–2000 m, 1.V.1985, E. Kvavadze leg; many ♂♂, ♀♀, juv. (SMNG), NW of Abano, 42.6115°N, 44.3742°E, 2270 m a.s.l., 06.VII.2019, K. Voigtländer leg.; 1 ♂ (SMNG), Georgia, Telavi, in a house garden, 41.9240°N, 45.4670°E, 09.VII.2019, K. Voigtländer leg.; many ♂♂ and ♀♀ (SMNG), Tusheti, Pirikita Alazani Valley, close to Girevi, 42°29'42.1"N, 45°28'43.2"E, stony pasture at river bank, 2010 m a.s.l, 6.IX.2009, F. Walther leg.; many ♂♂, ♀♀, juv. (SMNG), Samtskhe-Javakheti, 500 m W of Gujarula Valley, 41.3078°N, 43.4672°E, 1010 m a.s.l, 17.IX.2011, F. Walther leg.; 2 ♂♂, 3 ♀♀, 1 juv. (SMNG), Imereti, Zekari Pass, 41.8522°N, 42.8078°E, 2200 m a.s.l., 22.VIII.2014, F. Walther leg.; 1 ♂ (SMNG), Khevsureti, Churo Valley, near mouth of Khone Skali River, 42°34'05.8"N, 45°13'56.2"E, 10–12.IX.2009, F. Walther leg.; 1 ♂, 1 ♀ (SMNG), Mtshkheta-Mtianeti, 1.1 km SW of Kobi, 42.5553°N, 44.4973°E, 2060 m a.s.l., 12.VIII.2014, F. Walther leg.; 2 ♂♂ (SMNG), Mukhli, bridge over Rioni River, 100 m from the village; 42.5436°N, 43.2325°E, 640 m a.s.l., 9.X.2011, F. Walther leg.; 1 ♂ (SMNG), Tusheti, Diklo, 42°23'24.0"N, 45°40'41.8"E, 2100 m a.s.l., strongly eroded place near stream, 4.IX.2009, collector?; 1 ♂, 3 ♀♀ (SMNG), Akhaltsikhe to Borjomi, S of Chobiskhevi, near Chobiskhevi River, 42.7651°N, 43.3265°E, mountain *Fagus* forest, VII.2011, L. Mumladze leg.; 1 ♂, 2 ♀♀ (SMNG), N of Lagodekhi, Lagodekhi Nature Reserve, Lagodekhiskhevi River, mixed forest, VII.2013, L. Mumladze leg.; **Russia**: 1 ♂ (SMNG), Stavropol, Tamanskaya Dacha Forest, near Komsomolskiy Reservoir, 45.0481°N, 41.9567°E, 530 m a.s.l., deciduous forest (*Quercus*, *Carpinus*, *Acer*), 22.VIII.2012, F. Walther leg.; 2 ♂♂ (ZMUM), Stavropol, Russkiy Forest, 6.VII.2013, R. V. Zuev leg. and det.; many ♂♂, ♀♀, juv. (ZMUM), Stavropol Province, E of Novopavlovsk, *Quercus* forest, *Salix*, *Alnus* etc. along stream, 28.V.1982, SIG leg.; many ♂♂, ♀♀, juv. (ZMUM), Republic of Dagestan, Sergokalinskiy District, Murguk, mixed forest, near a river, 10.VIII.1985, K. Khajialiyev leg.; many ♂♂, ♀♀, juv. (ZMUM), Republic of Dagestan, Samur Valley, Garakh ca. 35 km SW of Magaramkent, 700–800 m, *Crataegus*, *Quercus*, *Acer*, *Rosa* etc. scrub, in litter, 23.X.1987, SIG leg.; 4 ♂♂, 3 ♀♀, 2 juv. (ZMUM), Republic of Dagestan, Chiragchay Valley, Khiv, 900–950 m, *Carpinus*, *Fagus*, *Corylus*, *Rhododendron* etc. forest., in litter and under stones, 24.X.1987, SIG leg.; 2 ♂♂, 1 ♀, 1 juv. (HNHM), Republic of Dagestan, Kurush, 2400 m a.s.l., under stones, 9.VII.1989, Z. Korsós leg.

#### Diagnosis.

A species of *Omobrachyiulus* differing from congeners mainly by the opisthomere having a thumb-like apical outgrowth of the basoposterior process, bent somewhat anteriad, and by the solenomere ending with a fine, short, sharply pointed, apical process.

#### Descriptive notes.

**Figure 12.**
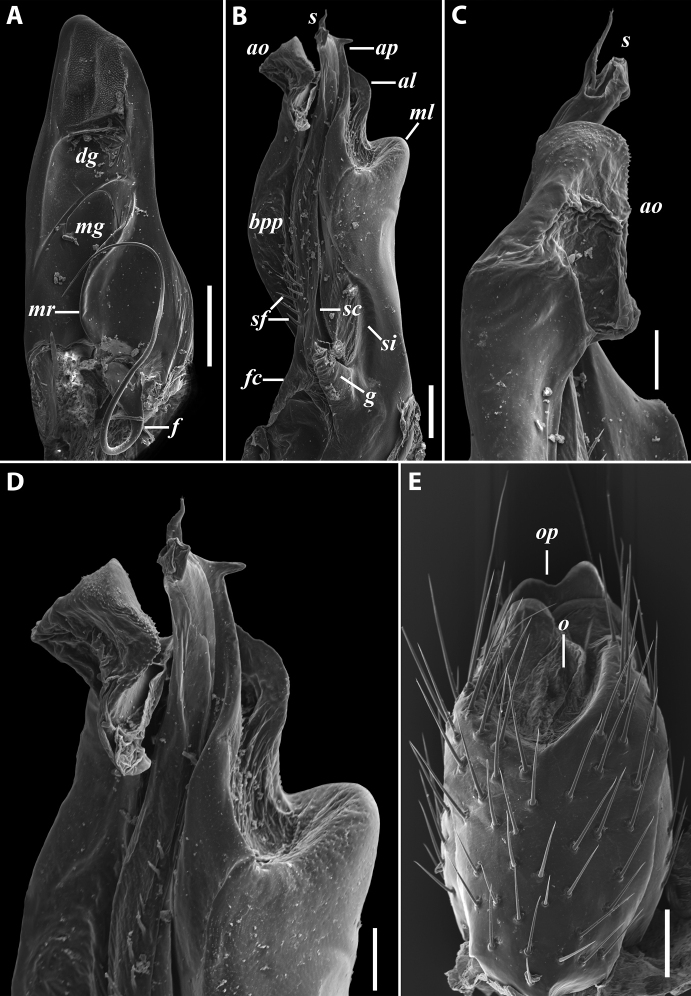
*Omobrachyiuluscaucasicus* (Karsch, 1881) comb. nov., ♂ (**A–D**) and ♀ (**E**) from S of Zərgəran, Azerbaijan (SMNG) **A–D** gonopods **A** right promere, caudal view **B** right opisthomere, mesal view **C** apical part of the same, meso-caudal view **D** distal part of right opisthomere, mesal view **E** right vulva, caudal view. Scale bars: 0.2 mm (**A**), 0.1 mm (**B, E**), 0.05 mm (**C, D**). Abbreviations: ***al*** apicolateral part of mesomeroidal lobe, ***ao*** apical outgrowth of basoposterior process, ***ap*** anterior process, ***bpp*** basoposterior process, ***dg*** distal groove, ***f*** flagellum, ***fc*** flagellum channel, ***g*** (supposed) gonocoxal gland, ***mg*** median groove, ***ml*** mesomeroidal lobe, ***mr*** median ridge, ***o*** opening, ***op*** operculum, ***s*** solenomere, ***sc*** seminal channel, ***sf*** spiniform filaments, ***si*** anteromesal sinus. Pictures by courtesy of Karin Voigtländer, Hans Reip and Dragan Antić.

***Gonopods*:** Promere (Fig. 12A) more or less tapering to a mesally slanting apex, median ridge well-pronounced, arched, median and distal grooves broad and deep; flagellum nearly as high as promere. Opisthomere (Fig. 12B–D): basoposterior process relatively well-pronounced, ending in a roughly thumb-like (in lateral view) apical outgrowth, mesally with a small lamellar part; anterior process running parallel to solenomere, mostly fused to the latter, apically forming a thorn-like outgrowth directed frontad; mesomeroidal lobe well-developed, forming a rounded apicolateral part; mesal side with a rather small lobe (presumably gonocoxal gland), and a small and shallow anteromesal sinus; two rows of rather long spiniform filaments along proximal section of flagellum channel; seminal channel clearly visible; solenomere apically with a very slender, pointed, lateral process, and a much shorter and thicker, lamellar, mesal one. ***Vulva*** (Fig. 12E) somewhat asymmetrical: mesal valve somewhat broader than lateral one, and mesal side of operculum protruding higher than lateral one; opening broad cleft-like; operculum slightly higher than bursa; both bursa and operculum densely setose throughout.

#### Previous records from the Caucasus.

Numerous records from **Georgia**; **Russia**: Republic of Dagestan (Vagalinski and Lazányi 2018); and **Azerbaijan** (Loginova 1993; Bababekova 1996).

#### General distribution.

Also found in Russia, Kalmykia (Golovatch and Matyukhin 2011), the Hyrcan area of Azerbaijan, northern and northwestern Iran, northeastern Turkey, and the island of Thassos, Greece (the subspecies *O.C.thassensis*).

#### Comment.

Revision of the holotype of *Juluscaucasicus* Karsch, 1881 revealed that the juvenile specimen belongs to the tribe Brachyiulini. This can be inferred from the relatively short epiproct with the apex turned dorsad, in combination with the ozopores set tightly behind the pro-metazonal suture. Of the Caucasian Brachyiulini occurring in the region of Borjomi, only *O.brachyurus* (Attems, 1899) and *O.macrourus* Lohmander (Lohmander, 1928) show the aforementioned condition of the epiproct. However, in *O.macrourus* the latter is both much longer and broader, as the name of this species implies. Furthermore, the dorsolaterally dark trunk and contrasting yellow legs (according to the original description) is exactly the colour pattern seen in *O.brachyurus*. Thus, there can be little doubt that *O.brachyurus* is a junior subjective synonym of *J.caucasicus*. Lohmander (1936), in his monograph on the Caucasian Diplopoda, omitted *J.caucasicus* altogether. The earlier use of the proper name (Kokhia 1999) was based on an unpublished identification of material by one of us (SIG), thus being neither a new formal transfer nor of nomenclatural consequences.

#### Remarks.

The species is new to the fauna of Armenia, also being among the most common and widespread in the Caucasus region.

The subspecies *O.C.dagestanus* was described by Lohmander (1936) on the basis of a single male originating from Kumukh, in the south-central part of the Republic of Dagestan. Comparing the original description and drawings of *dagestanus* to the description and drawings referring to the nominate subspecies found in that same paper, the main diagnostic characters of *dagestanus* seem to be the more narrow promere with a straight rather than medially bulging lateral margin, and the opisthomere having a less strongly pronounced, more slanting mesomeroidal lobe and a smaller, tapering apical outgrowth of its basoposterior process. While the promere varies considerably throughout the species’ distribution range, with males from Stavropol and the Hyrcanian part of Azerbaijan showing a similar shape to that seen in the *dagestanus* holotype, the mesomeroidal lobe and the basoposterior process’ apical outgrowth in the examined males from Dagestan are more or less consistent with those depicted by Lohmander (1936), and the aforementioned shapes of the two structures seem to be characteristic of the population that inhabits the northeastern parts of the Caucasus Major. Whether *dagestanus* is a subspecies/geographic variation of the typical *O.caucasicus* or a separate species is a question that probably requires molecular analyses to receive a reliable answer.

Another subspecies, *O.C.thassensis* (Mauriès, 1985), was described from the Greek island of Thassos (Mauriès 1985). The large distance between the Aegean region and the Caucasus aside, the highly distinct shape of the basoposterior process’ apical outgrowth and the very weakly pronounced mesomeroidal lobe seen in the original figures seem to be enough as evidence of a separate species involved. However, no formal taxonomic changes are proposed here yet, pending the accumulation of more comparative information, both morphology- and molecular-based.

### Omobrachyiulus curvocaudatus

Taxon classificationAnimaliaJulidaJulidae

(Lignau, 1903)

5F498907-D49D-5224-927D-DB24CA8A282B

Julus curvocaudatus Lignau, 1903: 135–136, figs 55–58.
Chromatoiulus (Omobrachyiulus) curvocaudatus : Lohmander, 1936: 126–129, figs 108–110; Lang 1959: 1791.
Chromatoiulus curvocaudatus : Kobakhidze 1964: 191; 1965: 394.
Megaphyllum curvocaudatum : Talikadze 1984: 143; Chumachenko 2016: 409.
Omobrachyiulus curvocaudatus : Vagalinski and Lazányi 2018: 96; Kokhia and Golovatch 2018: 41; 2020: 206.

#### Material examined.

**Georgia**: 1 ♂ (SMNG), Mtshkheta-Mtianeti, forest SW of Lake Bazaleti, 42.0308°N, 44.6653°E, 910 m a.s.l., 4.X.2011, F. Walther leg.; 1 ♂ (ZMUM), Racha-Lechkhum-Kvemo Svaneti Planned National Park, 10 km NE of Shovi, Gurshevi near Mamisoni Pass, 2000–2200 m, *Abies*, *Fagus*, *Alnus*, litter and under stones, at a spring, 21.X.1981, SIG leg.; 1 ♂ (ZMUM), AR Abkhazia, Pitsunda-Myussera Nature Reserve, Myussera part, 20–130 m, mixed deciduous forest (*Castanea*, *Alnus* etc.), in litter, under bark and stones, 8–10.IV.1983, SIG leg. 1 ♂ (ZMUM), AR Abkhazia, environs of Lake Ritsa, in litter, mixed forest, 24.X.1978, SIG leg.; 2 ♂♂, 6 ♀♀ (ZMUM), AR Abkhazia, Myussera Forest, 13.VI.1978, V. Dolin leg.; 1 ♂ (ZMUM), AR Abkhazia, Ochamchira District., near Otapi, at the entrance to Cave Golova Otapa, II.1983, S. Smirnov leg.; 1 ♂, 3 ♀♀ (ZMUM), AR Ajara, Batumi Botanical Garden, 15–20 m, 30.V–7.VI.1981, SIG and J. Martens leg. **Russia**: 1 ♂ (SMNG), Republic of Adygea, Lagonakskiy Ridge, limestone cliffs on the SW slope of Mount Gora Matuk, 44.1061°N, 39.9225°E, 1810 m a.s.l., 16.VIII.2012, F. Walther leg.

#### Diagnosis.

A species of *Omobrachyiulus* differing from congeners mainly by the opisthomere bearing a large, flattened, roughly diamond-shaped, apical outgrowth of the basoposterior process, and a shortly bifurcate solenomere, with the anterior branch being clavate, and the posterior one fine and pointed, bent anteriad.

#### Previous records from the Caucasus.

**Russia**: Krasnodar Province, Pseashkho Pass (type locality); Sochi (Lohmander 1936); **Georgia**: AR Abkhazia, Gagra and Avadkhara (Kobakhidze 1965).

#### General distribution.

CAUC.

### Omobrachyiulus divaricatus

Taxon classificationAnimaliaJulidaJulidae

(Lohmander, 1936)

1D8AA424-F53D-5D0D-98AA-18B0CB907110

Fig. 13

Chromatoiulus (Omobrachyiulus) divaricatus Lohmander, 1936: 135–140, figs 117–119.
Chromatoiulus (Omobrachyiulus) divaricatus : Lang 1959: 1791.
Chromatoiulus divaricatus : Kobakhidze 1964: 191; 1965: 394; Bababekova 1996: 90.
Megaphyllum divaricatum : Talikadze 1984: 143.
Omobrachyiulus divaricatus : Vagalinski and Lazányi 2018: 96–97, fig. 89; Kokhia and Golovatch 2018: 41; 2020: 206.

#### Material examined.

**Armenia**: 3 ♂♂, 2 ♀♀ (ZMUM), “Prov. Zori, Mount Polat”, 30.VIII.1925, A. Schelkovnikov leg. [probably H. Lohmander det.]. **Georgia**: 1 ♂ (SMNG), Khevsureti, ca. 200 m below Shatili, 42°39'34.8"N, 45°09'55.0"E, 1415 m a.s.l., 12–15.IX.2009, F. Walther leg.; 1 ♂, 4 ♀♀ (SMNG), Guria, Chkhakoura, towards Bakhmaro, 41.8797°N, 42.3511°E, 1780 m a.s.l., 21.VIII.2014, F. Walther leg.; many ♂♂, ♀♀, juv. (SMNG), Samtskhe-Javakheti, Mtsvanne Monastery, 41.8094°N, 43.3122°E, 840 m a.s.l., 24.VIII.2014, F. Walther leg.; 4 ♂♂, 3 ♀♀, 3 juv. (SMNG), Samegrelo-Zemo Svaneti, 3 km NE of Ushguli, 42.9347°N, 43.0358°E, 19.VIII.2014, F. Walther leg.; 1 ♂, 7 ♀♀ (SMNG), Samtskhe-Javakheti, Bakuriani, ca. 9 km to Tskhratskaro, Ugheltekhili Pass, 41.7070°N, 43.5028°E, 2210 m a.s.l., 22.VIII.2014, F. Walther leg.; 1 ♂ (SMNG), Imereti, Sairme, towards Zekari Pass, 41.8572°N, 42.7894°E, 1920 m a.s.l., 22.VIII.2014, F. Walther leg.; 1 ♂ (SMNG), Mtshketa-Mtianeti, Zhinvali towards Shatili, at rkm 41/65, 42.4031°N, 44.9281°E, 1090 m a.s.l., 8.VIII.2014, F. Walther leg.; 1 ♂ (SMNG), Khashuri, Akhaldaba, Nedzvi Managed Reserve, Nedzvistskali River, 41.9082°N, 43.5151°E, humid coniferous (*Picea*) forest, VI.2011, L. Mumladze leg.; 2 ♂, 1 ♀ (SMNG), AR Ajara, Goderdzis Ugheltekhili Pass, W slope, 41.6483°N, 42.4936°E, 1730 m a.s.l., 3.X.2012, F. Walther leg.; 1 ♂, 2 ♀♀ (SMNG), Racha-Lechkhumi and Kvemo Svaneti, 3 km S of Mravaldzali, 41.8797°N, 42.3511°E, 1900 m a.s.l., 9.X.2011, F. Walther leg.; many ♂♂, ♀♀, juv. (SNMG), Shida Kartli, Gagluantubani, Tana Valley, 24.1 road-km upstream Didi Ateni, 41.8508°N, 43.8658°E, 1290 m a.s.l., 16.IX.2011, F. Walther leg.; 3 ♂♂, 8 ♀♀ (ZMUM), near Manglisi, *Quercus* forest, 12.XI.1984, E. Kvavadze leg.; many ♂♂, ♀♀, juv. (ZMUM), Racha, Oni District, N of Utsera, deciduous forest near a spring, rock, in litter, 20.IX.1981, SIG leg.; 1 ♂, 1 ♀ (ZMUM), Tsodoreti near Tbilisi, in litter, 24.XII.1974, unknown collector; 2 ♂♂, 3 ♀♀ (ZMUM), Mukhura, ca. 14 km E of Tkibuli, 650 m, *Castanea*, *Acer* etc. forest, in litter, under logs, 18.X.1987, SIG leg.; many ♂♂, ♀♀, juv. (ZMUM), Republic of South Ossetia – Alania, 15 km E of Kvaisi, Suram Mountain Ridge, Ertso Pass, ca. 1000 m, deciduous bush along spring, in litter and under stones, 20.X.1981, SIG leg.; 2 ♂♂, 3 ♀♀, 2 juv. (ZMUM), 40 km W of Mestia, Kherkhvashi E of Nakra, 1250–1700 m, *Quercus*, *Fagus*, *Carpinus*, *Picea*, *Abies* etc. forest, litter and bark, 21.VIII–21.IX.1986, SIG leg.; 1 ♂, 5 ♀♀ (ZMUM), Kutaisi District, 8 km E of Orpiri, environs of Cave Tsutskhvati, deciduous forest, rock, litter, 24.X.1981, SIG leg.

#### Diagnosis.

**Figure 13.**
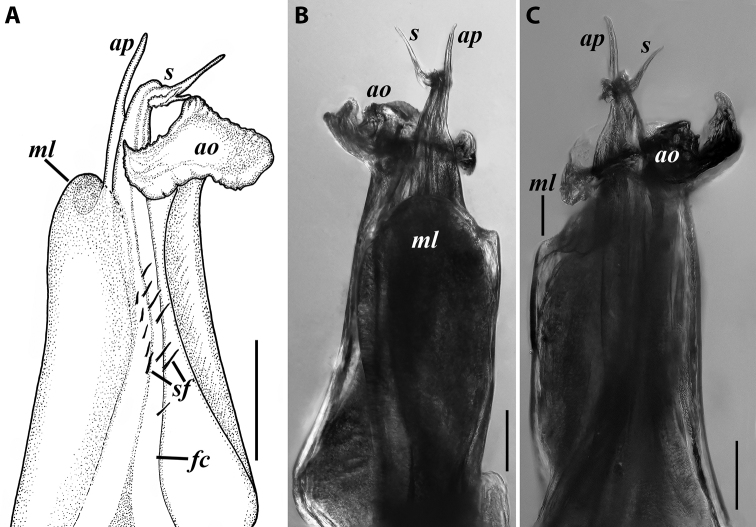
*Omobrachyiulusdivaricatus* (Lohmander, 1936), gonopods of ♂ from near Nakra, Georgia (ZMUM) **A** left opisthomere, mesal view **B** same, oral, slightly lateral view **C** same, caudal, slightly mesal view. Scale bars: 0.2 mm (**A**), 0.1 mm (**B, C**). Abbreviations: ***ao*** apical outgrowth of basoposterior process, ***ap*** anterior process, ***fc*** flagellum channel, ***ml*** mesomeroidal lobe, **s** solenomere, ***sf*** spiniform filaments.

A species of *Omobrachyiulus* very similar to *O.unugulis* sp. nov. by the general shape of the promere and by the opisthomere (Fig. 13) having a slender, rod-like, anterior process directed distad, a broad, strongly flattened, multilobed, apical outgrowth of the basoposterior process, and the similarly finely and asymmetrically bifurcate solenomere. Differs from the latter species by the following gonopodal characters: promere with a more narrowly rounded apex; apical outgrowth of opisthomeral basoposterior process nearly symmetrical, tripartite, vs. the same being asymmetrically bilobed, forming a dentate distal part and a thumb-like lateral part in *O.unugulis* sp. nov.; anterior process of opisthomere equal to or slightly higher than solenomere, vs. the same being significantly shorter than solenomere in *O.unugulis* sp. nov.; solenomere abruptly bent caudad rather than nearly straight and directed almost completely distad; and by the male hypoproct being penta- or septidentate rather than tridentate.

#### Descriptive notes.

***Opisthomere*** (Fig. 13A–C): basoposterior process weakly pronounced, ending in a nearly symmetrical tripartite apical outgrowth; anterior process very long and slender rod-like, reaching the level of, or slightly exceeding, solenomere; mesomeroidal lobe rather well-pronounced, with a rounded apex, not forming an apicolateral part; two rows of rather small spiniform filaments at mid-section of flagellum channel; solenomere, bent abruptly distad before a very fine and slender apical process directed distocaudad.

#### Previous records from the Caucasus.

**Georgia**: shore of Lake Tabatskuri; Turkey/Georgia, Ajara, “Post Arsian” [most likely a locality in the Arsiani Range shared between Turkey and Georgia] (original localities); Ipari; Rveli; Lentekhi District: Ushguli; Koruldashi; at the foot of Mt Shkhara (Kobakhidze 1965); AR Ajara, Kintrishi Nature Reserve, mouth of Khekhpara River (Vagalinski and Lazányi 2018); **Armenia**: Dilijan (original locality).

#### General distribution.

Caucasian endemic with a broad distribution south of the Greater Caucasus watershed; not reported north of the latter.

### Omobrachyiulus geniculatus

Taxon classificationAnimaliaJulidaJulidae

(Lohmander, 1928)

CD0F6BD1-06F7-5DF2-B6E8-631130287C4A

Chromatoiulus geniculatus Lohmander, 1928: 236–238, figs 12–14.
Chromatoiulus (Omobrachyiulus) geniculatus : Lohmander, 1936: 129–131, figs 108–110; Lang 1959: 1791.
Omobrachyiulus geniculatus : Vagalinski and Lazányi 2018: 97.

#### Material examined.

**Georgia**: 1 ♂ (SMNG), Mtskheta-Mtianeti, Stepantsminda: mouth of river Snotskali, 42.6339°N, 44.6258°E, 1750 m a.s.l., 07.X.2012, F. Walther leg., 1 ♂ (SMNG), Mtskheta-Mtianeti, Darial Gorge at rkm 8/131, 42.7039°N, 44.6264°E, 1480 m a.s.l., 10.VIII.2014, F. Walther leg.; **Russia**: 3 ♂♂, 1 ♀, 6 juv. (ZMUM), Karachay-Cherkess Republic, Teberda Nature Reserve, Kizgich Canyon N of Arkhyz, near a riverine, *Alnus* and *Betula* wet forests, under bark and stones, 1450–1500 a.s.l., 5.VI.1985, SIG leg.; 1 ♂ (ZMUM), Chechen Republic, Assa Valley, ca. 9 km SSW of Muzhichi, 800 m, *Fagus*, *Alnus*, *Carpinus* etc. forest, in litter, under bark and stones, 15.VII.1986, SIG leg.

#### Diagnosis.

A species of *Omobrachyiulus* differing from congeners by the following combination of opisthomere characters: basoposterior process in the shape of a strongly pronounced lobe forming a nearly right-angled corner at base, ending with a simple and smooth apical outgrowth with a blunt tip; anterior process shaped as a stout spine; mesomeroidal lobe strongly pronounced; solenomere simple, bent strongly caudad.

#### Previous records from the Caucasus.

**Russia**, Karachay-Cherkess Republic, by Bolshoy Zelenchuk River (type locality), “Post Akssaut” [probably on Aksaut River] (Lohmander 1936); Kabardino-Balkar Republic, Bisinghi [Bezengi] Valley (Vagalinski and Lazányi 2018).

#### General distribution.

GRCA.

#### Remark.

The species is new to the fauna of Georgia.

### Omobrachyiulus macrourus

Taxon classificationAnimaliaJulidaJulidae

(Lohmander, 1928)

0D3006B6-27FE-5A93-8E2F-BC2376206771

Chromatoiulus macrourus Lohmander, 1928: 542–544, figs 15–17.
Chromatoiulus (Omobrachyiulus) macrourus abchasicus Lohmander, 1936: 131–135, figs 111–114.
Chromatoiulus (Omobrachyiulus) macrourus macrourus : Lang 1959: 1791.
Chromatoiulus (Omobrachyiulus) macrourus abchasicus : Lang 1959: 1791.
Chromatoiulus macrourus abchasicus : Kobakhidze 1964: 191; 1965: 393.
Chromatoiulus macrourus macrourus : Kobakhidze 1964: 191; 1965: 393.
Megaphyllum macrourum macrourum : Talikadze 1984: 143.
Megaphyllum macrourum abchasicum : Talikadze 1984: 143.
Omobrachyiulus macrourus : Vagalinski and Lazányi 2018: 98; Kokhia and Golovatch 2018: 41; 2020: 207.

#### Material examined.

**Georgia**: 2 ♂♂ (SMNG), Akhaltsikhe to Borjomi, S of Chobiskhevi, at Chobiskhevi River, 42.7651°N, 43.3265°E, mountain *Fagus* forest, VII.2011, L. Mumladze leg.; 1 ♂ (SMNG), Samegrelo-Zemo Svaneti, Nikortsminda, Skhartali, near the entrance to Sakishore Cave, 42.4420°N, 43.1590°E, *Picea* and *Abies* forest, under dead wood, 15.VI.2019, H. Reip leg.; 1 ♂, 12 ♀♀ (ZMUM), Kherkhvashi E of Nakra, 1250–1700 m, *Quercus*, *Fagus*, *Carpinus*, *Abies* etc. forest, in litter and under bark, 21.VIII–21.IX.1986, SIG leg.; 2 ♂♂, 1 ♀ (ZMUM), Chokhatauri District near Bakhmaro, 4 km SSE of Nabeglavi, 600 m, *Alnus* along stream, 8.VI.1981, SIG and J. Martens leg.; 1 ♂, 4 ♀♀, 3 juv. (ZMUM), Tbilisi, Tskhneti, 1150–1300 m, *Fagus*, *Carpinus*, *Acer* etc. forest, 16–18.V.1987, SIG and K. Eskov leg.; 1 ♂, 2 ♀♀ (ZMUM), Mukhura, ca. 15 km E of Tkibuli, 700–800 m, *Castanea*, *Fagus*, *Carpinus* etc. forest, in litter, under bark and stones, 7–9.V.1987, SIG and K. Eskov leg.; 2 ♂♂, 3 ♀♀ (ZMUM), environs of Manglisi, *Quercus* forest, 12.XI.1984, E. Kvavadze leg.; 3 ♂♂, 9 ♀♀, 4 juv. (ZMUM), Algeti National Park, W of Manglisi, *Fagus*, *Picea*, *Acer* etc., 1400–1450 m, litter and under bark, 16–18.V.1987, SIG and K. Eskov leg. **Russia**: 5 ♂♂, 10 ♀♀ (ZMUM), Chechen Republic, Argun Valley, 5 km N of Shatoy, *Corylus*, *Fagus*, *Carpinus* etc. forest, 750 m, in litter, under stones and bark, 18.VII.1986, SIG leg.; 1 ♂ (NHMD), Karachay-Cherkess Republic, ca. 15 km SW of Teberda, 1500 m a.s.l., *Fagus-Picea* forest, pitfall traps, 22–28.VIII.2011, A. Solodovnikov and S. Tarasov leg.

#### Diagnosis.

A species of *Omobrachyiulus* closely resembling *O.divaricatus* and *O.unugulis* sp. nov. by the general shape of both pro- and opisthomere. Differs from these two species mostly by the bulging pillow-shaped, rather than antero-caudally strongly flattened, apical outgrowth of the basoposterior process, as well as by the very large (both long and broad) epiproct.

#### Previous records from the Caucasus.

**Georgia**: Tbilisi; Borjomi (original localities); Zeskho near Lentekhi (Kobakhidze 1965); AR Abkhazia, at the mouth of Kelassuri River [S of Sukhumi], passage cave (type locality of *O.m.abchasicus*); Klych River valley (Kobakhidze 1965).

#### General distribution.

CAUC.

#### Remarks.

The species is new to the fauna of Russia.

The subspecies *O.m.abchasicus* was described by Lohmander (1936) from near Sukhumi, AR Abkhazia. The author emphasised the high similarity between the typical *O.macrourus* and his newly described subspecies, but failed to present a diagnosis to distinguish between the two forms. The sole diagnostic character than can be inferred from the original description of *abchasicus* is the presence of a small conical lobe distally at the apical outgrowth of the basoposterior process (z in figs 111, 113), that lobe being absent from the typical form. Considering the slight to moderate shape variations in the apical outgrowth in some of the examined specimens originating from different parts of the species’ distribution range, *O.m.abchasicus* does not look like a viable taxon. However, in the absence of material from Abkhazia we prefer to refrain from a formal synonymisation with the typical *O.macrourus*.

### Omobrachyiulus unugulis

Taxon classificationAnimaliaJulidaJulidae

Vagalinski sp. nov.

0941EA70-CE9D-595E-8BCE-1FAFB9B2D21A

http://zoobank.org/3D6B8739-C27B-4151-ABAF-36CFCA9A4AE0

Figs 14 , 15 , 16

#### Material examined

**(SMNG). *Holotype***: ♂ (in head to ring 6, pleurotergum 7 to ring 29, and rest of body, gonopods, left antenna, right flange of pleurotergum 7, hypoproct, leg 6, a mid-body, and an end-body leg dissected), Georgia, Samegrelo-Zemo Svaneti, Mestia, source of the valley above Lengeri, 43.0742°N, 42.6983°E, 2060 m a.s.l., 27.IX.2012, F. Walther leg. ***Paratype***: 1 subadult ♂ (in head and collum and rest of body), same collecting data as for holotype.

#### Diagnosis.

A species of *Omobrachyiulus* very similar to *O.divaricatus* by the general shape of the promere and by the opisthomere having a slender, rod-like, anterior process directed distad, a broad, strongly flattened, multilobed, apical outgrowth of the basoposterior process, and a similarly finely and asymmetrically bifurcate solenomere. Differs from *O.divaricatus* by the following gonopodal characters: promere with a more broadly rounded apex; apical outgrowth of opisthomeral basoposterior process asymmetrically bilobed, forming a dentate distal part and a thumb-like lateral part, vs. the same being more or less symmetrical, tripartite in *O.divaricatus*; anterior process of opisthomere significantly lower rather than equal to, or higher than, solenomere; solenomere mostly straight, directed almost completely distad, vs. the same being abruptly bent caudad in *O.divarictus*; and by the male hypoproct being tri-, rather than penta- or septidentate.

#### Name.

Derived from the Latin *unugula* meaning paw, after the apical outgrowth of the basoposterior process of the opisthomere, which resembles an animal foot with claws and a large thumb. Adjective.

#### Description.

***Measurements*:** holotype ♂ in S in XI, 50+1+T, L = 29 mm, H = 1.95 mm. Paratype subadult ♂ in S X, 47+2+T.

**Figure 14.**
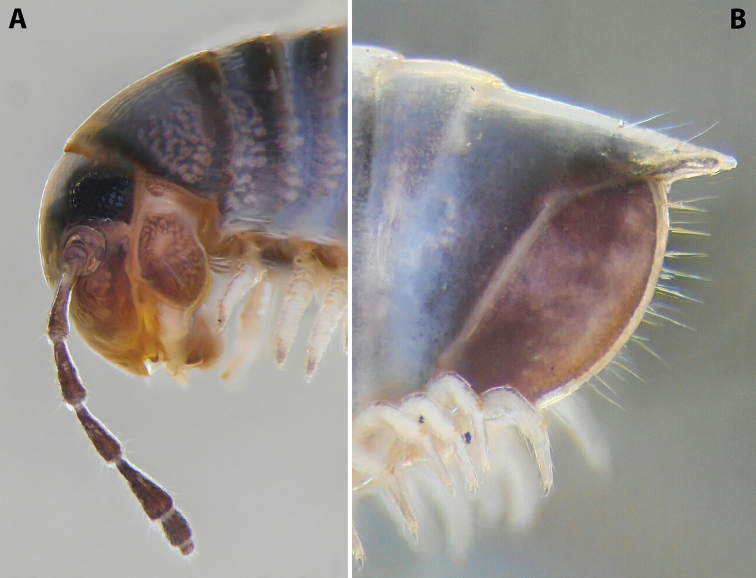
*Omobrachyiulusunugulis* sp. nov., ♂ holotype **A** head and body rings 1–3 **B** telson, lateral views. Not to scale.

***Colouration*** (Fig. 14): Mostly brown-grey with purple tinges; head brown, with the usual pattern; collum mostly grey, dorsally brownish, with dark brown margins; prozonae grey, ventrolaterally with numerous light spots; metazonae grey, with a transverse dark brown stripe in posterior half, this abruptly narrowing below ozopore level, hind margins light brown-beige; dorsum with a black axial line; epiproct dark brown-grey, paraprocts somewhat lighter brown; legs light yellow.

***External structures*:** Eye patches consisting of ca. 45 ommatidia arranged in easily recognisable vertical rows. Vertigial, supralabral, and labral setae: two, four, and 18, respectively. Antennae (Fig. 14A) ca. 1.7 × as long as head in males; antennomere 2 > 5 ≥ 3 > 4 > 6. Gnathochilarium with promentum of moderate size, separating lamellae linguales in ca. 2/5 of their length, each latter with three setae in a longitudinal row. Collum mostly smooth, with several short and shallow striae at posterolateral corners.

Body rings not vaulted. Prozonae with numerous minute grooves in posterior third. Metazonae relatively shallowly striated, n*_Schub_* = 10 or 11; setae (apparently) mostly abraded, rather short based on the few remaining. Ozopores set tightly behind pro-metazonal suture in more anterior rings, gradually moved further back, to nearly equal to their diameter behind the suture in caudalmost rings, sutures gently sinuous in front of ozopores in some rings. Tarsus of mid-body legs slightly shorter than tibia, and slightly over 3 × as long as apical claw.

**Figure 15.**
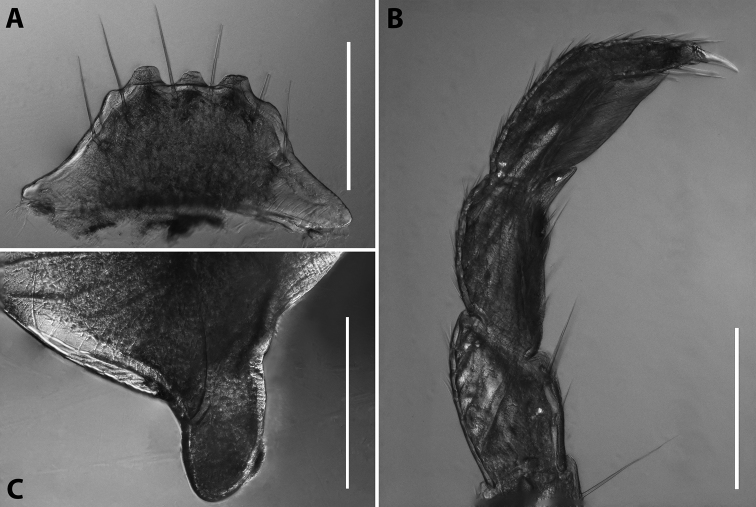
*Omobrachyiulusunugulis* sp. nov., ♂ holotype (SMNG) **A** hypoproct, ventral view **B** distal three podomeres of leg 6 **C** left flange of pleurotergum 7, ventro-lateral view. Scale bars: 0.3 mm.

***Telson*** (Fig. 14B): Epiproct moderately long, straight, broad, roof-like, ending with a short and blunt hyaline tip equal to, to slightly surpassed by, the longest paraproctal setae. Hypoproct (in males) (Fig. 15A) broadly trapezoidal, tridentate, ventrally with six submarginal setae, only the teeth protruding behind rear contour of paraprocts. Paraprocts moderately densely setose, with a row of sparse short (ca. half as long as the ‘regular’ ones) and stiff setae along each caudal margin.

***Male sexual characters*:** Mandibular stipites (in Fig. 14A) considerably expanded, protruding mostly anteriad, forming a narrow anterior corner. Leg pair 1 compact parallel. Walking legs (Fig. 15B) with well-developed crested pads, both tibial and postfemoral ones gradually reduced towards telson, but still visible in caudalmost pairs; femora without modifications. Pleurotergum 7 (Fig. 15C) ventrally forming rather slender and rounded lobes originating from the zone around pro-metazonal suture, protruding mostly ventrad behind gonopods.

**Figure 16.**
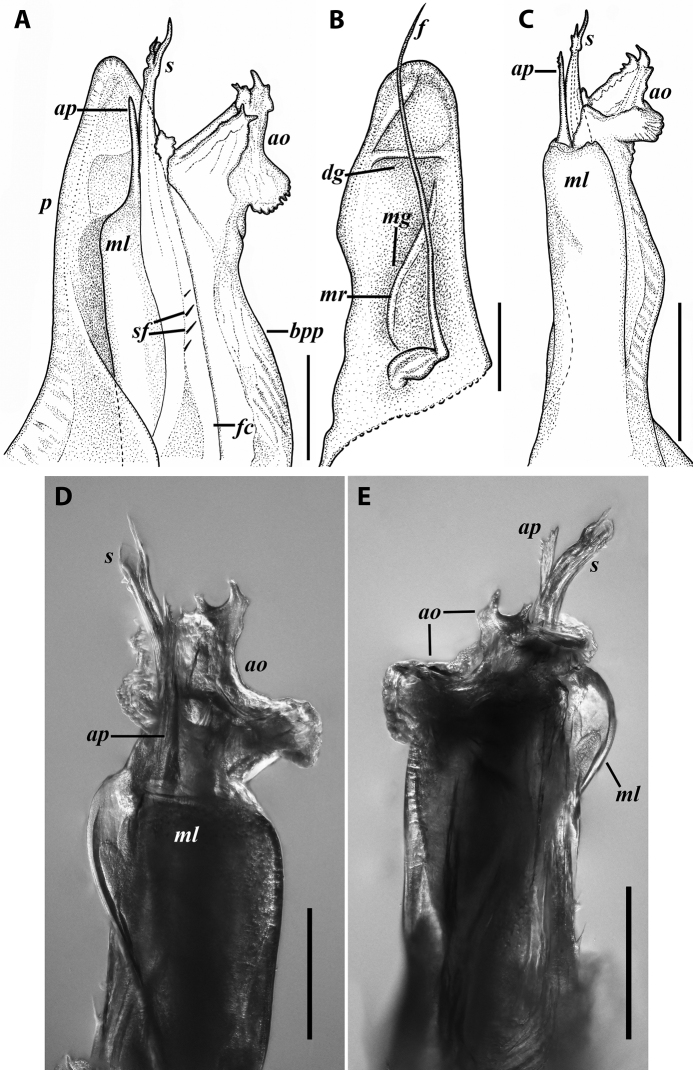
*Omobrachyiulusunugulis* sp. nov., gonopods of ♂ holotype **A** left gonopods (without the basal parts), mesal view **B** right promere, caudal view **C** right opisthomere, oral-lateral view **D** distal half of the same, oral view **E** same, caudal view. Scale bars: 0.4 mm (**C**), 0.3 mm (**B**), 0.2 mm (**A, D, E**). Abbreviations: ***ao*** apical outgrowth of basoposterior process, ***ap*** anterior process, ***bpp*** basoposterior process, ***fc*** flagellum channel, ***ml*** mesomeroidal lobe, ***p*** promere, ***s*** solenomere, ***sf*** spiniform filaments.

***Gonopods*** (Fig. 16): Promere (Fig. 16B, p in Fig. 16A) relatively slender, broadest at base, mesal margin gently concave, lateral one bulging in mid-section, both joining into a broadly rounded apex; caudal surface with a strongly pronounced and relatively long median ridge, a narrow and rather deep median groove, and a broad and deep, but indistinctly marked, distal groove; flagellum just slightly longer than height of promere. Opisthomere (Fig. 16A, C–E) rather slender; basoposterior process shaped as a weakly pronounced, hump-like lobe, distally forming a broad, bilobed, apical outgrowth: with an apically dentate distal lobe, and a rounded thumb-like lateral lobe; anterior process well-developed, slender and rod-like, with a slightly serrate apical part; mesomeroidal lobe large, but rather weakly pronounced, flattened; mesal side with a large lobe (presumably gonocoxal gland), and a very deep anteromesal sinus; several minute spiniform filaments along flagellum channel; solenomere long and slender, significantly exceeding anterior process; apically finely and asymmetrically bifurcate, directed almost completely distad and only slightly caudad.

***Female sexual characters*:** unknown.

#### Remarks.

Despite the considerable morphological similarity between *Omobrachyiulusdivaricatus* and *O.unugulis* sp. nov., they undoubtedly represent two separate species, this being inferred from the few, but significant differences concerning gonopodal characters. Furthermore, the gonopod structure of *O.divaricatus* is remarkably consistent (in comparison with the condition in, e.g., *O.caucasicus*), with almost no visible variations between specimens from all over the species’ distribution area.

#### General distribution.

SWGC.

##### The *hortensis* group

**Characterisation.** Promere significantly higher than opisthomere, distolaterally with a micro-squamose lobe or field; with a distinct distal groove. Opisthomere stout, compact, with a weakly to moderately pronounced mesomeroidal lobe, a basoposterior process with a moderately to well-developed proximal part, ending with a variously shaped apical outgrowth, a ridge-like anterior process of various size, being mostly fused to solenomere, a deep anteromesal sinus, a flagellum channel overgrown with very long, erect, spiniform filaments, and a simple, finger- or rod-like solenomere bent more or less caudad. Vulva with operculum shorter than bursa.

**Included species.**

*O.hortensis* (Golovatch, 1981)

*O.armatus* Vagalinski, sp. nov.

*O.pristis* Vagalinski, sp. nov.

### Omobrachyiulus hortensis

Taxon classificationAnimaliaJulidaJulidae

(Golovatch, 1981)

CA380A8E-3E42-597D-BF8E-4971D5A39B52

Fig. 17A

Chromatoiulus hortensis Golovatch, 1981: 110–112, figs 14–26.
Megaphyllum hortense : Talikadze 1984: 143.
Omobrachyiulus hortensis : Vagalinski and Lazányi 2018: 97; Kokhia and Golovatch 2018: 41; 2020: 206.

#### Material examined.

**Georgia**: 2 ♂♂, 2 ♀♀ (ZMUM), NE of Poti, Chaladidi, *Alnus*, *Quercus*, *Fraxinus* forest on swamp, in litter, 13.IV.1983, SIG leg.; 1 ♂, 1 ♀ (ZMUM), AR Abkhazia, Sukhum District, Nizhnyaya Yashtukha, forest nursery, 29.III.1985, А. Markossian; 1 ♂, 2 juv. (ZMUM), same place, tobacco plantation, 16.VI.1980 and 25.VII.1980, А. Markossian.

#### Diagnosis.

A species of *Omobrachyiulus* most similar to *O.armatus* sp. nov. by the promere significantly outreaching the opisthomere, the latter possessing a massive lobe-like basoposterior process forming two distinct corners, a basal and a distal one, and having a micro-spiculate mesal side, and a unipartite solenomere with a slender rod-like ending. Differs from *O.armatus* sp. nov. mainly by the clearly tripartite apical outgrowth of the basoposterior process, consisting of a mesal lamellar, a median fan-shaped, and a lateral spiniform part, vs. that same outgrowth being broad, unipartite, collar-shaped and dentate at the margins in the latter species.

#### Descriptive notes.

**Figure 17.**
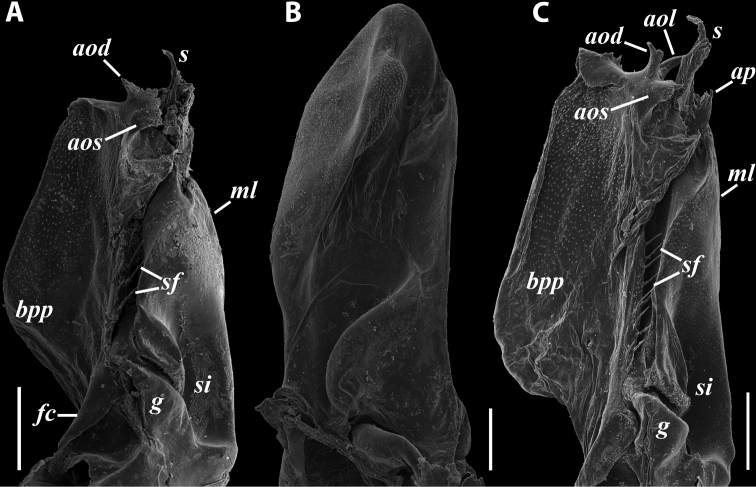
**A***Omobrachyiulushortensis* (Golovatch, 1981), ♂ from near Poti, Georgia (ZMUM) **B, C**O.cf.hortensis from Mestia, Georgia (ZMUM) **B** right promere, caudal view **A, C** right opisthomere, mesal view. Scale bars: 0.1 mm. Abbreviations: ***aod*** median part of basoposterior process’ apical outgrowth, ***aol*** lateral part of basoposterior process’ apical outgrowth, ***aos*** mesal part of of basoposterior process’ apical outgrowth, ***ap*** anterior process, ***bpp*** basoposterior process, ***fc*** flagellum channel, ***g*** (supposed) gonocoxal gland, ***ml*** mesomeroidal lobe, ***s*** solenomere, ***sf*** spiniform filaments, ***si*** anteromesal sinus.

Promere rather slender, significantly outreaching the opisthomere, with a narrowly rounded apex; distolaterally micro-papillate. Opisthomere (Fig. 17A) short and stout; basoposterior process massive, with a micro-spiculate mesal side, and an apical outgrowth consisting of three parts: a mesal and a median one, both being fan-like and partly fused, and a lateral spine-like one (very similar to aol in Fig. 17C); an anterior process absent or vestigial; mesomeroidal lobe moderately developed; mesal side with a relatively large, but not freely protruding lobe (presumably gonocoxal gland), and a very large and deep anteromesal sinus; two rows of very long, erect, spiniform filaments parabasally at flagellum channel; solenomere unipartite, very fine and rod-like.

#### Previous records from the Caucasus.

**Georgia**: AR Abkhazia, Sukhum Botanical Garden (type locality).

#### General distribution.

COLC-SWGC.

### Omobrachyiulus cf. hortensis

Taxon classificationAnimaliaJulidaJulidae

(Golovatch, 1981)

20045851-659B-544A-ADE8-C329313F625C

Fig. 17B, C

#### Material examined.

**Georgia**: 3 ♂♂, 2 ♀♀ (ZMUM), Mestia, 1500 m a.s.l., *Betula*, *Rhododendron* on moraine, litter and under stones, 5 and 15.IX.1986, SIG leg.; 1 ♂ (SMNG), Mestia, below Chalaadi Glacier, 43.1118°N, 42.7453°E, *Abiesnordmanniana* and various deciduous trees, in leaf litter, 9.VI.2019, H. Reip leg.

#### Remarks.

The males in these two samples from Mestia differ from both the original description and drawings, and the currently studied material of *O.hortensis* by certain structural details of the opisthomere (Fig. 17C), viz., presence of a smaller or larger, pointed, anterior process and differently shaped mesal and median parts of the apical outgrowth of the basoposterior process (compare to Fig. 17A); as well as by a stouter, abruptly rather than gradually tapering, promere (Fig. 17B). While these specimens may represent a new, closely related species, we cannot exclude possibly intraspecific variations; the more so that there are no significant differences between the solenomere, the taxonomically most important structure in Julidae, in these two forms (compare s in Fig. 17A and Fig. 17C), while the promere and the anterior process of the opisthomere tend to be the most variable gonopodal structures within the Brachyiulini.

### Omobrachyiulus armatus

Taxon classificationAnimaliaJulidaJulidae

Vagalinski sp. nov.

B2D33B9F-8E9B-505D-9D49-8D7874C35D48

http://zoobank.org/2DB1AB90-F666-42EB-88E2-0A583F83A581

Figs 18 , 19 , 20

#### Material examined

**(all from Georgia). *Holotype***: ♂ (unbroken) (ZMUM), Manglisi, *Quercus* forest, 12.XI.1984, E. Kvavadze leg. ***Paratypes***: 2 ♂♂ (ZMUM) (one in head to ring 2, ring 3 to ring 6, pleurotergum 7, and rest of body; right antenna, leg pair 2, penis, legs 6, 7, and mid-body leg dissected, opisthomeres and left promere prepared for SEM; the other unbroken with removed hypoproct), 3 ♀♀ (ZMUM), same collecting data as for holotype; 1 ♂ (ZMUM) (partly broken in anterior section), 2 ♀♀ (ZMUM) (one unbroken, the other in 3 pieces), AR Ajara, E of Khulo, decidous forest near spring, rock, litter, 10.X.1981, SIG leg.; 2 ♂♂ (ZMUM) (one in three pieces, with dissected gonopods, the other with a missing posterior body half), 1 ♂ (NHMD) (unbroken), 1 ♂ (NMNHS) (in two pieces), 1 ♀ (NMNHS) (unbroken), Kutaisi District, Sataplia Nature Reserve, forest litter and under stones, 25.X.1981, SIG leg.; 1 ♂ (ZMUM) (in three pieces, with dissected gonopods), 1 ♂ (IBER) (unbroken), 5 ♀♀ (ZMUM) (unbroken), 7 juv. (ZMUM) (two unbroken, the rest in 2 or more fragments), AR Ajara, Khulo, 900 m, forest of *Quercus*, *Abies*, *Alnus*, litter, 11.X.1981, SIG leg.; 2 ♂♂, 3 ♀♀ (SMNG), Georgia, Samtskhe-Javakheti, 17.IX.2011, Bakuriani, Tskhratskaro Pass, 41.7067°N, 43.5058°E, 2080 m a.s.l., 17.IX.2011, F. Walther leg.

#### Diagnosis.

A species of *Omobrachyiulus* most similar to *O.hortensis* by the promere significantly outreaching the opisthomere, the latter possessing a massive lobe-like basoposterior process forming two distinct corners, a basal and a distal one, and having a micro-spiculate mesal side, and a unipartite solenomere with a slender rod-like ending. Differs from *O.hortensis* mainly by the broad, unipartite, collar-shaped, apical outgrowth of the basoposterior process dentate at margin, that same outgrowth being clearly tripartite in the latter species, consisting of a mesal lamellar, a median fan-like, and a lateral spiniform part.

#### Name.

Meaning armed in Latin, referring to the overall appearance of the male including the opisthomere bearing various spines and denticles, the large tapering mandibular stipites, and the prominent dentate hypoproct. Adjective.

#### Description.

***Measurements*:** holotype ♂ in S IX, 45+1+T, L = 17 mm, H = 1.4 mm; paratype ♂♂ in S IX, 44–46+1+T, L = 16–20 mm, H = 1.35–1.55 mm; paratype ♀♀ in S IX, 44–46+1+T, L = 17.5–22 mm, H = 1.7–1.9 mm.

**Figure 18.**
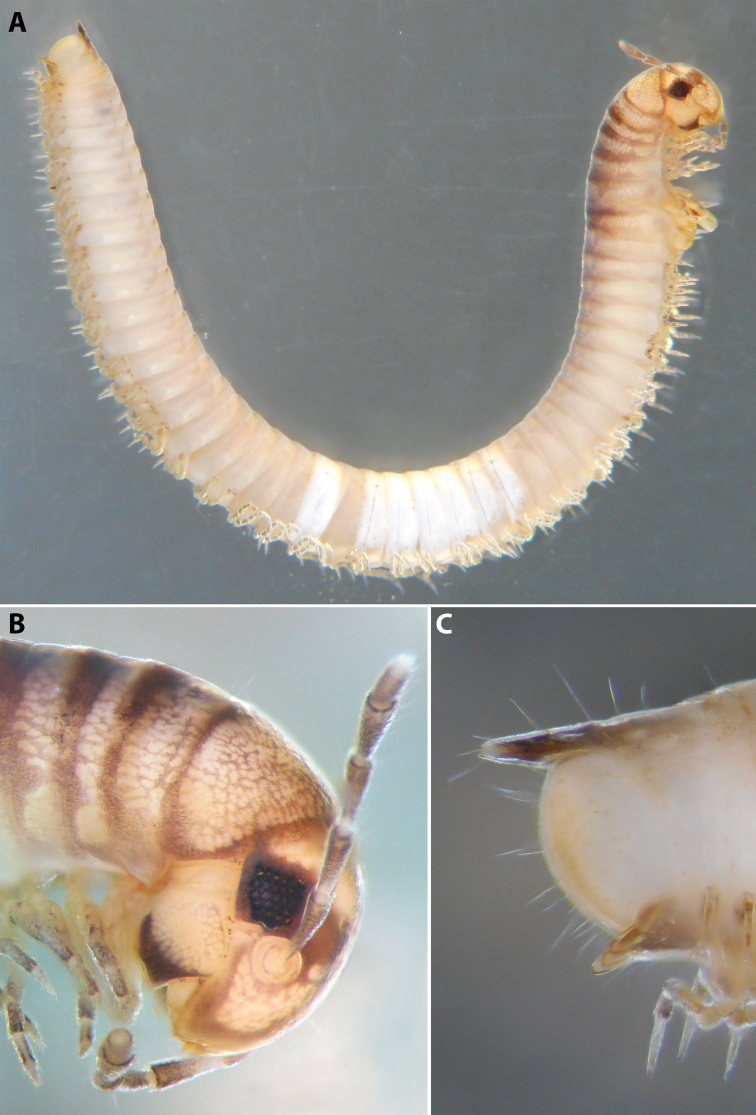
*Omobrachyiulusarmatus* sp. nov., ♂ holotype **A** habitus **B** head and body-rings 1–3 **C** telson, lateral views. Not to scale.

***Colouration*** (most specimens considerably faded in ethanol) (Fig. 18): Head light brown with the usual dark band between eye patches, distal part of male mandibular stipites saturated dark brown, contrasting to the much lighter proximal part; body mostly light brown, lighter ventrally; prozonae dorsally with dark grey to blackish transverse stripes; each metazona with a blurred dark brown band encircling entire ring; dorsum with a black axial line.

***External structures*:** Eye patches in adults consisting of 30–35 ommatidia, usually arranged in easily countable vertical rows. Vertigial, supralabral, and labral setae: two, four, and 19–23, respectively. Antennae (in Fig. 18B) ca. 1.6–1.7 × as long as head in males and 1.1–1.4 × in females; antennomere 2 > 3 = 4 = 5 > 6. Gnathochilarium with an elongated promentum, the latter separating both lamellae linguales > halfway; four setae in a longitudinal row on each lamella. Collum completely smooth.

Body rings barely vaulted. Prozonae with short and very shallow longitudinal striae spread across whole surface. Metazonae moderately deeply striated, n*_Schub_* = 7 or 8 in males and 9 or 10 in females; metazonal setae rather sparse, 2/3 (in middle to caudal parts of body)–3/4 (in frontal part of body) of metazonal length. Ozopores set tightly behind pro-metazonal suture in more anterior rings, gradually moved further backwards, to nearly equal to their diameter behind the suture in caudalmost rings; sutures straight to gently sinuous in front of ozopores. Tarsus of a mid-body leg slightly shorter than to equal to tibia, and nearly 3 × as long as apical claw.

**Figure 19.**
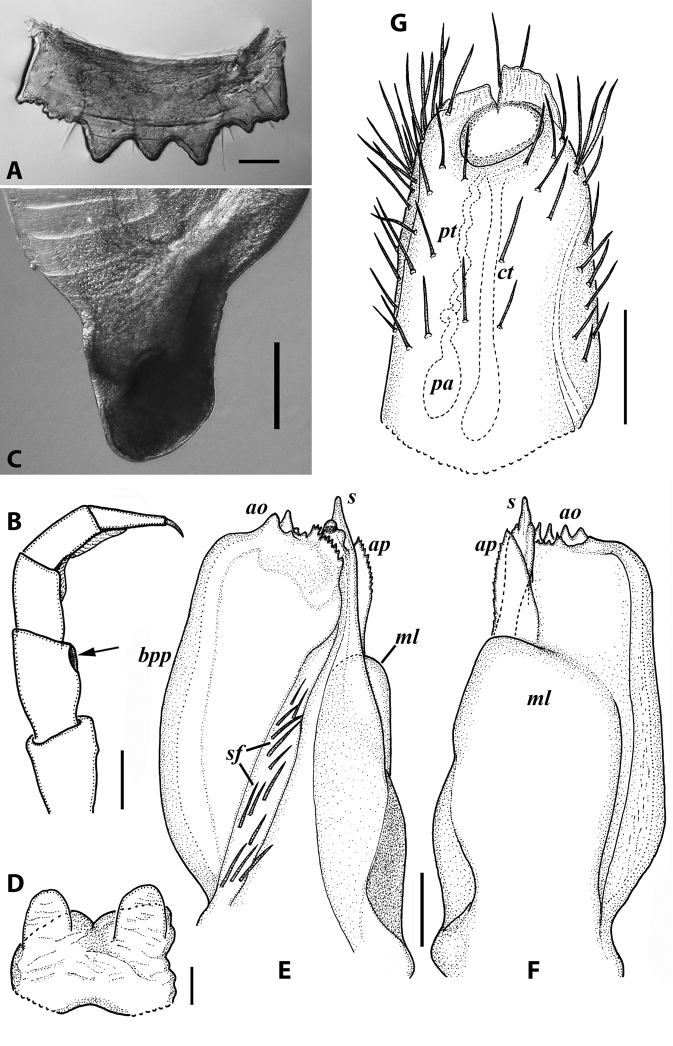
*Omobrachyiulusarmatus* sp. nov., ♂ (**A–F**) and ♀ (**G**) paratypes from near Khulo, AR Ajara, Georgia (ZMUM) **A** hypoproct, ventral view **B** leg 6 (coxa not shown) **C** right flange of pleurotergum 7, ventro-lateral view **D** penis, caudal view **E** right opisthomere, caudo-mesal view **F** same, latero-oral view **G** left vulva, caudal, slightly mesal view. Scale bars: 0.2 mm (**C, G**), 0.1 mm (**A, B, E, F**) 0.6 mm (**D**). Abbreviations: ***ao*** apical outgrowth of basoposterior process, ***ap*** anterior process, ***bpp*** basoposterior process, ***ct*** central tube, ***ml*** mesomeroidal lobe, ***pa*** posterior ampulla, ***pt*** posterior tube, ***s*** solenomere, ***sf*** spiniform filaments.

***Telson*** (Fig. 18C): Epiproct moderately long, stout, shorter than, or barely reaching, the level of longest paraproctal setae, ending bluntly, without a distinct, or with a poorly defined, hyaline tip; with several long setae dorsolaterally. Hypoproct (Fig. 19A) in males very broad, subrectangular, its margin with three large median teeth and two lower protuberances laterally on each side, the latter being sometimes serrate; in females broadly rounded, tightly adhering under paraprocts; ventral surface sparsely setose. Paraprocts moderately densely setose, without distinct row of shorter setae along caudal margins.

***Male sexual characters*:** Mandibular stipites (in Fig. 18B) strongly expanded, forming an acute anterior corner. Leg pair 1 compact rounded hooks, slightly turned against one another. Leg pair 2 and sometimes 3 slightly thicker than following pairs, adhesive pads rather weakly developed, becoming better developed towards mid-body, postfemoral ones disappearing in caudal third of body, tibial ones present until last leg pairs; femora from leg pair 5 to mid-body legs distally with a small shallow pit (arrow in Fig. 19B). Pleurotergum 7 considerably enlarged, ventrally forming large and rounded lobes (Fig. 19C) originating mostly from metazona, protruding caudoventrad behind gonopods. Penis (Fig. 19D) very short, slightly broader than long, compressed anterocaudally, with very short apical lobes ending in rounded terminal lamellae directed distad.

**Figure 20.**
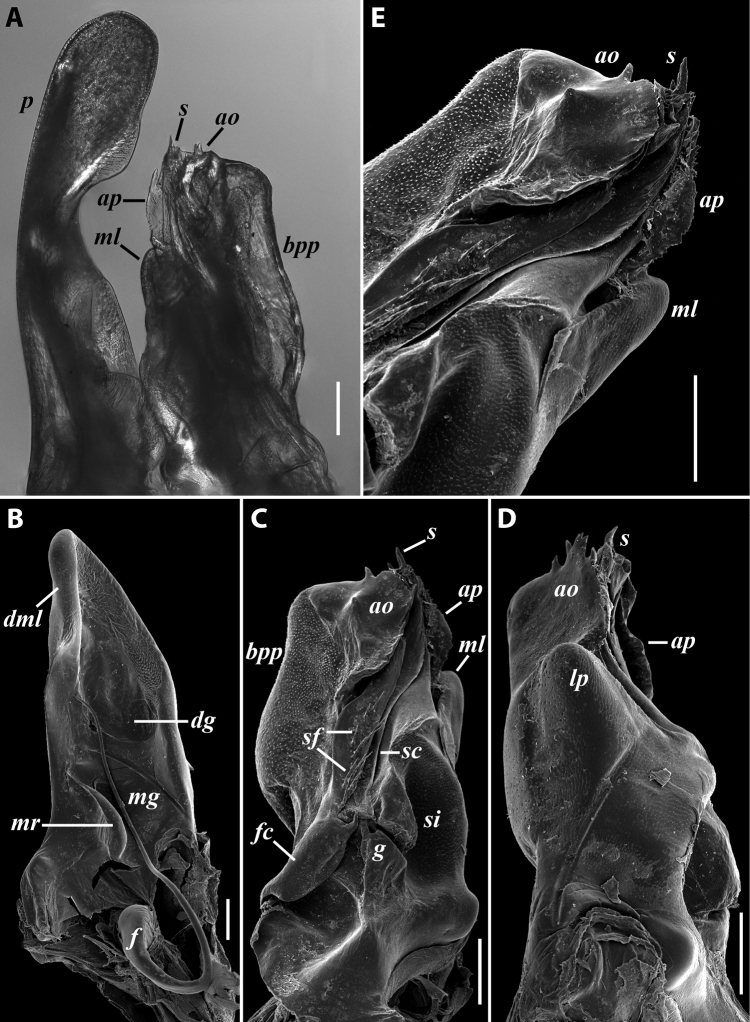
*Omobrachyiulusarmatus* sp. nov., gonopods of ♂ paratype from near Manglisi, Georgia (ZMUM) **A** left gonopods, mesal view **B** left promere, caudal view **C** right opisthomere, mesal view **D** left opisthomere, lateral view **E** distal part of right opisthomere, mesal view. Scale bars: 0.1 mm. Abbreviations: ***ao*** apical outgrowth of basoposterior process, ***ap*** anterior process, ***bpp*** basoposterior process, ***dg*** distal groove, ***f*** flagellum, ***fc*** flagellum channel, ***g*** (supposed) gonocoxal gland, ***lp*** lateral lobe, ***mg*** median groove, ***ml*** mesomeroidal lobe, ***mr*** median ridge, ***s*** solenomere, ***sc*** seminal channel, ***sf*** spiniform filaments, ***si*** anteromesal sinus.

***Gonopods*** (Figs 19E, F, 20): In situ considerably protruding from gonopodal sinus. Promere (Fig. 20B, p in 20A) considerably higher than opisthomere, slender; mesal margin mostly straight, lateral margin parallel to mesal one in proximal section, distally steeply slanting towards a blunt apex; caudal surface with a short, well-pronounced, median ridge, and a broad and rather shallow median groove; with a distinct, deep, distal groove, and a smooth, rounded, distomesal lobe directed caudad; flagellum slightly longer than height of promere. Opisthomere (Figs 19E, F, 20A, C–E) compact and robust; basoposterior process represented by a massive lobe with a micro-spiculate mesal surface, distally forming a very broad apical outgrowth bent anteriad, tightly adhering to caudal and partly to mesal side of solenomere, with several teeth/horns at margin; anterior process shaped as a lamellar ridge with a slightly serrate anterior margin; mesomeroidal lobe broad and rounded, moderately developed; a rounded hump-like lobe on lateral side; mesal side with a large lobe (presumably gonocoxal gland) leading to the seminal channel, and a large anteromesal sinus frontally to the gland; solenomere unipartite, mostly straight, relatively broad, apically with a fine digitiform process; a group of very long, erect, spiniform filaments in proximal section of flagellum channel.

***Female sexual characters*:** Leg pairs 1 and 2 shorter, leg pair 2 also thicker, than following legs. Vulva (Fig. 19G) mostly symmetrical (lateral valve somewhat more oblique than mesal one), rather elongate, slightly compressed on sides; bursa with an obtuse postero-apical margin; operculum shorter than bursa, both ending up with well-developed hyaline protrusions; setation denser on bursa than on operculum. Receptaculum seminis consisting of a long and mostly straight central tube with a somewhat enlarged bottom part, and a long and thin posterior tube forming several spiral twists on its way to an ovoid posterior ampulla.

#### General distribution.

LECA-COLC.

### Omobrachyiulus pristi

Taxon classificationAnimaliaJulidaJulidae

s Vagalinski sp. nov.

4CCB2E36-BE84-56A8-9A2F-7D285177C9BD

http://zoobank.org/A22C7D2A-412C-4F6E-B854-FC1165348E2C

Figs 21 , 22 , 23

#### Material examined

**(all from Georgia). *Holotype***: ♂ (unbroken) (ZMUM), AR Ajara, 15 km W of Adigeni, *Abies*, *Picea*, *Fagus*, *Acer*, etc., 1500–1700 m, litter, logs, under stones, 14–15.V.1983, SIG leg. ***Paratypes***: 1 ♂ (ZMUM) (in head and 4 pieces, gonopods, penis, legs 2, 3, 7 and a mid-body leg dissected), 1 ♂ (NHMD) (unbroken), 1 ♂ (NMNHS) (unbroken), 2 ♀♀ (ZMUM) (one unbroken, the other in 3 pieces with dissected left vulva), 1 ♀ (NHMD) (unbroken), 1 ♀ (NMNHS) (unbroken), 1 subad. ♀ (ZMUM) (unbroken), 5 juv. (ZMUM) (two unbroken, the others fragmented), same collecting data as for holotype; 1 ♂ (ZMUM) (in 3 pieces, gonopods prepared for SEM), AR Ajara, Khulo Distr., 1800 m a.s.l., Danisparauli, *Abies* and fern litter, 10.X.1981, SIG leg.; 1 ♂ (ZMUM) (in 5 pieces, with dissected gonopods), 2 ♀♀ (ZMUM) (one in 2 pieces, the other unbroken), AR Ajara, Khulo Distr., Goderdzi Pass, 2000 m, sparse *Abies* and *Picea* forest, litter, under stones and in rotten logs, 10.X.1981, SIG leg.; 3 ♂♂ (SMNG) (two in 2 pieces with dissected gonopods, one unbroken), 1 ♂ (IBER) (in head to ring 6, pleurotergum 7 to ring 9, and rest of body, gonopods dissected), 3 ♀♀ (SMNG) (all unbroken), 1 ♀ (IBER) (unbroken), 1 juv., one head to ring 6 ♂ fragment (SMNG), Sairme Gorge, 41.9580°N, 42.7713°E, 600 m a.s.l., *Fagusorientalis* and *Castaneasativa*, in litter, 14.VII.2013, L. Mumladze leg.; 3 ♂♂ (SMNG) (one unbroken, one in 2 pieces with intact gonopods, one in 3 pieces with dissected gonopods), Sairme Gorge, 41.8468°N, 42.8075°E, 2200 m a.s.l., *Acer* sp. and *Betula* sp., 11.VII.2013, L. Mumladze leg.

#### Diagnosis.

A species of *Omobrachyiulus* most clearly differing from congeners by the caudally flattened, serrate, elongated, apical outgrowth of the basoposterior process of opisthomere, and by the conspicuously large anterior process which is almost as high as the solenomere.

#### Name.

To emphasise the caudally flattened, serrate, apical outgrowth of the basoposterior process of opisthomere, resembling the rostrum in the sawfishes of the family Pristidae when observed from caudal or caudomesal view. Noun in apposition.

#### Description.

***Measurements*:** holotype ♂ in S IX, 41+1+T, L = 15 mm, H = 1.3 mm; paratype ♂♂ in S IX–X, 41–44+1+T, L = 15–17 mm, H = 1.25–1.4 mm; paratype ♀♀ in S IX–X, 38–42+1–2+T, L = 15–19 mm, H = 1.4–1.65 mm.

**Figure 21.**
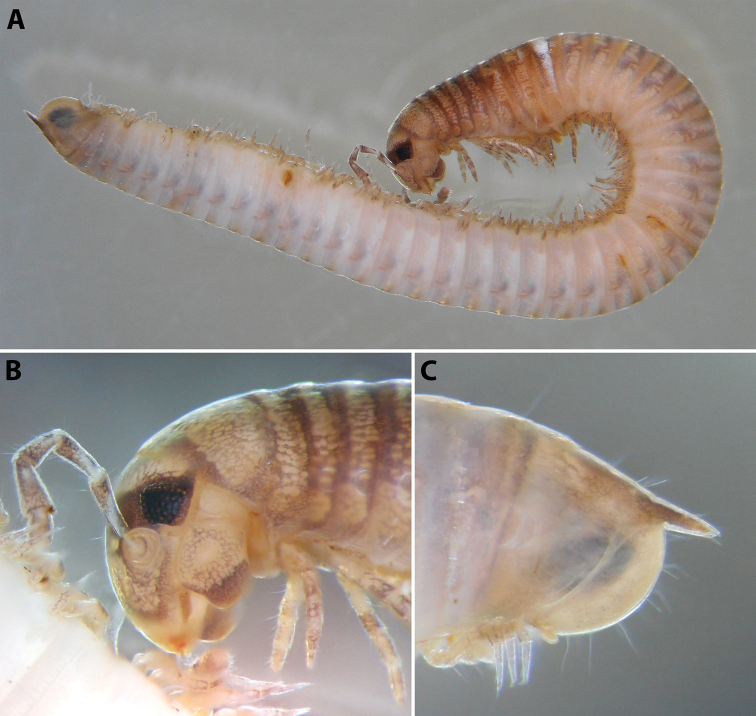
*Omobrachyiuluspristis* sp. nov., ♂ holotype **A** habitus **B** head and body rings 1–4 **C** telson, lateral views. Not to scale.

***Colouration*** (considerably faded from ethanol) (Fig. 21): Prozonae dorsolaterally dark grey to blackish, ventrolaterally dark grey near pro-metazonal suture; metazonae dorsolaterally with a transverse dark brown stripe; ozopores surrounded by an irregular blackish spot; ventrolateral side of body mostly light brown-beige; dorsum with a black axial line.

***External structures*:** Eye patches in adults consisting of 30–35 ommatidia, mostly arranged in easily countable vertical rows. Vertigial, supralabral, and labral setae: two, four, and 22–26, respectively. Antennae (in Fig. 21B) 1.40–1.55 × as long as head in males and 1.25–1.45 × in females; antennomere 2 > 3 = 5 > 4 > 6. Gnathochilarium with a relatively small promentum separating lamellae linguales by < 1/3 of their length, each latter with five setae in a longitudinal row; stipites in males basally with a kidney-shaped swelling densely covered with fine and stiff setae. Collum mostly smooth, with only several very short and fine striae near posterolateral corner.

Body rings somewhat vaulted (more significantly in caudal part of body). Prozonae with very short and fine longitudinal striae in their hind sections. Metazonae rather shallowly striated, n*_Schub_* = 8 or 9 in males and 11 or 12 in females; metazonal setae 2/5–1/2 of metazonal length. Ozopores set tightly behind pro-metazonal suture in more anterior rings, gradually moved further back, to nearly equal to their diameter behind the suture in caudalmost rings, sutures not sinuous in front of ozopores. Tarsus of mid-body legs 1.1–1.2 as long as tibia and ca. 4 × as long as apical claw.

***Telson*** (in Fig. 21C): Epiproct long (reaching the level of longest paraproctal setae), straight or slightly bent dorsad, ending with a minute hyaline tip. Hypoproct broadly rounded, ventrally with two median submarginal setae; barely protruding behind rear contour of paraprocts in males, tightly adhering to their ventral side in females. Paraprocts sparsely to moderately densely covered with long setae; no distinct rows of shorter setae along caudal margins.

**Figure 22.**
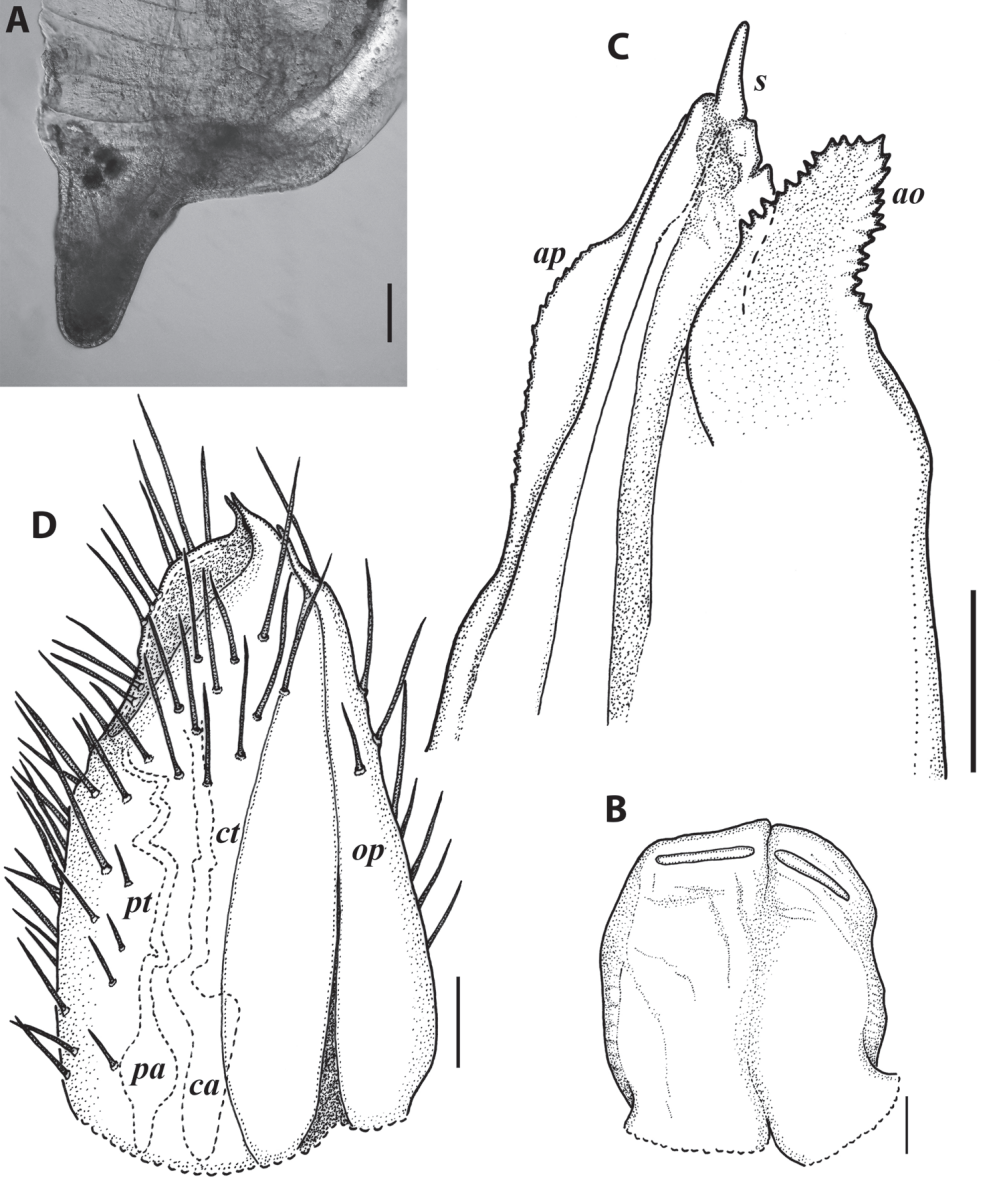
*Omobrachyiuluspristis* sp. nov., ♂ (**A–C**) and ♀ (**D**) paratypes from near Adigeni, AR Ajara, Georgia (ZMUM) **A** right flange of pleurotergum 7, ventro-lateral view **B** penis, caudal view **C** distal part of left opisthomere, caudo-mesal view **D** left vulva, caudo-lateral view. Scale bars: 0.1 mm (**A, D**), 0.06 mm (**C**), 0.05 mm (**B**). Abbreviations: ***ao*** apical outgrowth of basoposterior process, ***ap*** anterior process, ***ca*** central ampulla, ***ct*** central tube, ***pa*** posterior ampulla, ***pt*** posterior tube, ***s*** solenomere.

***Male sexual characters*:** Mandibular stipites (in Fig. 21B) considerably expanded, broadly rounded, protruding mostly ventrad, with either an indistinct or strongly obtuse and blunt ventro-anterior corner. Leg pair 1 compact hooks, slightly to considerably turned against one another. Leg pair 2 slightly thicker than following legs, with a weakly pronounced adhesive pad distoventrally on tibia, without pad on postfemur; following pairs with two pads, one each on postfemur and tibia, both clearly discernible until caudalmost pairs; femora without modifications. Pleurotergum 7 strongly enlarged, ventrally forming rather slender, fingertip-shaped lobes (Fig. 22A) originating entirely from metazona, protruding ventrad behind gonopods. Penis (Fig. 22B) somewhat longer than broad, with very short, barely discernible apical lobes, and with broad, triangular, terminal lamellae directed completely caudad.

**Figure 23.**
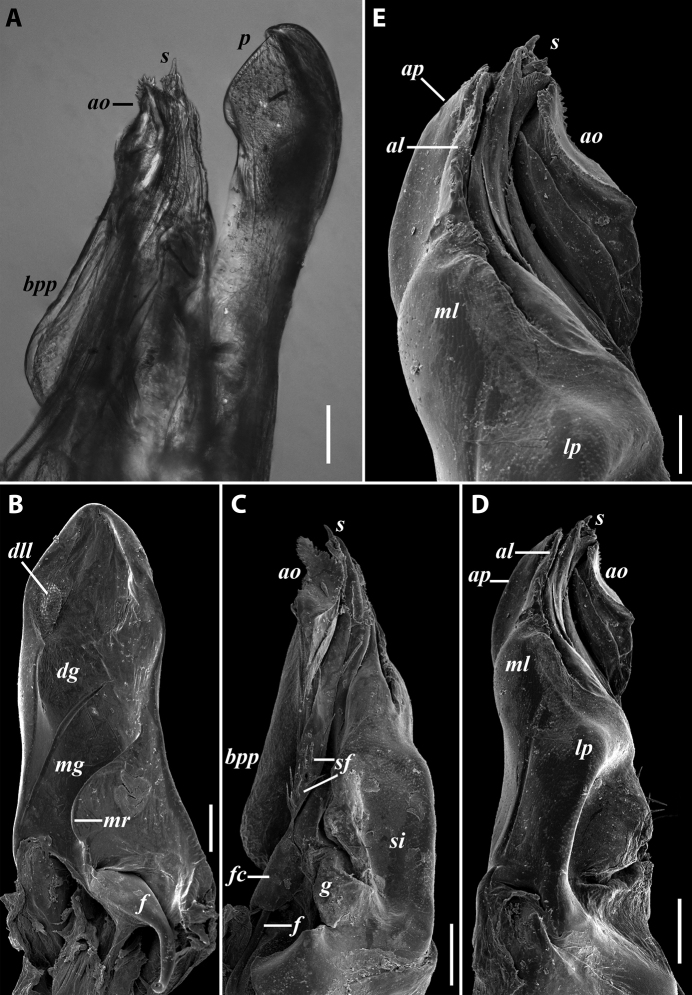
*Omobrachyiuluspristis* sp. nov., gonopods of ♂ paratypes from Adigeni (**A**) and Danisparauli (**B–E**), AR Ajara, Georgia (ZMUM) **A** right gonopods, mesal view **B** right promere, caudal view **C** right opisthomere (with flagellum in its channel), caudo-mesal view **D** right opisthomere, lateral view **E** distal part of the same aspect. Scale bars: 0.1 mm (**A–D**), 0.05 mm (**E**). Abbreviations: ***al*** apicolateral part of mesomeroidal lobe, ***ao*** apical outgrowth of basoposterior process, ***ap*** anterior process, ***bpp*** basoposterior process, ***dg*** distal groove, ***dl*** distolateral lobe, ***f*** flagellum (broken near base), ***fc*** flagellum channel, ***g*** (supposed) gonocoxal gland, ***lp*** lateral lobe, ***mg*** median groove, ***ml*** mesomeroidal lobe, ***mr*** median ridge, ***p*** promere, ***s*** solenomere, ***sc*** seminal channel, ***sf*** spiniform filaments, ***si*** anteromesal sinus.

***Gonopods*** (Figs 22C, 23): In situ considerably protruding from gonopodal sinus. Promere (Fig. 23B, p in Fig. 23A) considerably higher than opisthomere, relatively slender; mesal margin more or less straight, lateral margin gently sinuous, distally both margins symmetrically slanting towards a narrowly rounded apex; caudal surface with a rather short, strongly pronounced, median ridge, a relatively deep and broad median groove, a broad distal groove, and a small, rounded, micro-squamous, distolateral lobe; flagellum somewhat longer than height of promere, distally with rows of blunt teeth directed basad. Opisthomere (Figs 22C, 23A, C–E) relatively elongated; basoposterior process shaped like a weakly pronounced lobe, distally forming a rather narrow apical outgrowth with a flat caudal surface and densely serrate/dentate margins; anterior process large, distally tapering, bent caudad, reaching just below tip of solenomere; mesomeroidal lobe broad and rounded, weakly pronounced, positioned slightly above opisthomeral mid-height, forming a slender apicolateral part reaching as high as anterior process, proximally fused to the latter; lateral side with a rounded hump-like lobe; mesal side with a broad and flattened lobe (presumably gonocoxal gland), and a rather deep and spacious anteromesal sinus; a group of very long, erect, spiniform filaments at proximal section of flagellum channel; solenomere unipartite, tubular, relatively narrow, bent caudad, meso-apically with a fine digitiform process.

***Female sexual characters*:** Leg pairs 1 and 2 markedly thicker, first also shorter, than following legs. Vulva (Fig. 22D) mostly symmetrical, elongated, considerably compressed on sides; bursa with a strongly obtuse postero-apical margin; operculum slightly shorter than bursa, both parts ending with small hyaline protrusions; setation dense throughout. Receptaculum seminis consisting of a rather narrow, mostly straight, central tube, abruptly arched posteriad right before junction with a large, oblong, central ampulla, and a very narrow, somewhat folded, posterior tube leading to a rather large, pyriform, posterior ampulla.

#### Remark.

This species possesses some peculiar morphological characters, being the only representative of *Omobrachyiulus* that shows a vulval seminal receptaculum with a differentiated central ampulla, as well as the only one that lacks postfemoral adhesive pads on male leg pair 2.

#### General distribution.

LECA.

##### The *implicitus* group

**Characterisation.** Both gonopod pairs subequal in height. Promere possessing a distinct distal groove. Opisthomere rather slender, with a weakly to moderately pronounced mesomeroidal lobe, a basoposterior process with a weakly developed proximal part in the shape of a lamellar ridge, ending with a broad, collar-like, apical outgrowth showing a soft mesal part, a vestigial (if present) anterior process in the shape of an indistinct ridge, a narrow and not too deep anteromesal sinus, a flagellum channel overgrown with sparse, short to moderately long, spiniform filaments, and a slender, tubular, unipartite or distally branched solenomere directed mostly distad. Vulva with operculum shorter than, to subequal to, bursa.

**Included species.**

*O.implicitus* (Lohmander, 1936)

*O.fasciatus* Vagalinski, sp. nov.

*O.lazanyiae* Vagalinski, sp. nov.

### Omobrachyiulus implicitus

Taxon classificationAnimaliaJulidaJulidae

(Lohmander, 1936)

1E3B6D74-F39B-5D9D-A785-83358A61C42A

Fig. 24

Chromatoiulus (Omobrachyiulus) implicitus Lohmander, 1936: 140–143, figs 120–122.
Chromatoiulus (Omobrachyiulus) implicitus : Lang 1959: 1791.
Chromatoiulus implicitus : Kobakhidze 1964: 191; 1965: 394.
Chromatoiulus implicitus ritsensis Golovatch, 1981: 108–110, figs 8–11, syn. nov.
Megaphyllum implicitum implicitum : Talikadze 1984: 143.
Megaphyllum implicitum ritsense : Talikadze 1984: 143.
Megaphyllum implicatum (sic!): Chumachenko 2016: 409.
Omobrachyiulus implicitus : Vagalinski and Lazányi 2018: 98; Kokhia and Golovatch 2018: 41; 2020: 206.

#### Material examined.

**Georgia**: AR Abkhazia: 6 ♂♂, 7 ♀♀, 1 juv. (ZMUM), Pitsunda-Myussera Nature Reserve, Myussera part, 120–130 m a.s.l., mixed deciduous forest (*Castanea*, *Alnus* etc.), in litter, under bark and stones, 8–10.IV.1983, SIG leg.; 1 ♂, 3 ♀♀, 1 juv. (ZMUM), Avadhara, 1600–1700 m a.s.l., *Abies*, *Fagus*, fern, *Rubus*, *Galium*, 18.IX.1985, I.A. Ushakov leg.; 2 ♂♂, 3 ♀♀, 1 juv. (ZMUM), N of Lake Ritsa, *Abies*, *Fagus*, in litter, 13.IX.1985, I.A. Ushakov leg. **Russia**: Republic of Adygea: 1 ♂, 2 ♀♀ (AE), near Maykop, Polkovnitskaya Ravine, 44°20.72'N, 40°11.37'E, pitfall traps, 01–17.X.2011, Yu. Chumachenko leg.; 1 ♂, 1 juv. ♂ (AE), same place, collecting method and collector, 17.X–01.XI.2011; 1 ♂, 1 juv. (ZMUM), confluence of Kisha and Belaya rivers, mixed forest, ca. 200 m a.s.l., 11.VI.2013, R. V. Zuev leg.; 5 ♂♂, 6 ♀♀, 5 juv. (ZMUM), Krasnodar Province, 5.5 km NE of Krasnaya Polyana, lower course of Achipse River, 43°15'25"N, 40°15'25"E, ca. 600 m a.s.l., 19–23.VIII.2014, K. Makarov and A. Matalin leg.

#### Diagnosis.

A species of *Omobrachyiulus* most similar to *O.fasciatus* sp. nov. and *O.lazanyiae* sp. nov. by the overall shape of the promere, the weakly developed basoposterior process and mesomeroidal lobe of the opisthomere, and the broad, collar- or scarf-shaped, apical outgrowth of the basoposterior process, the latter having a characteristic, wrinkled, lamellar part partially covering the mesal side of the solenomere. Differs from these two species mainly by the very slender and tripartite solenomere apically forked into two mostly symmetrical, strongly diverging branches, with a minute ear-like lobe mesally at the base of the bifurcation; and by the distally broader promere with a very broad and deep distal groove on the caudal surface, this being a small pit in *O.fasciatus* sp. nov. and *O.lazanyiae* sp. nov.; as well as in other gonopod details summarised in a tabular key (Table 1) to these three species.

**Table 1.** Key to the species of the *Omobrachyiulusimplicitus* group based on male gonopodal and external somatic characters.

Character	* O. implicitus *	*O.fasciatus* sp. nov.	*O.lazanyiae* sp. nov.
length	17–19 mm	14–16 mm	12–13 mm
epiproct	with a distinct hyaline tip (usually) turned somewhat dorsad	as in *O.implicitus* (Fig. 25B)	straight, ending bluntly without distinct hyaline tip (Fig. 28B)
promere	basal and distal parts of nearly the same width; distolateral lobe weakly to moderately pronounced (Fig. 24A)	distally more or less tapering; distolateral lobe strongly pronounced (Fig. 27A, B)	of proportions intermediate between *O.implicitus* and *O.fasciatus* sp. nov.; distolateral lobe weakly pronounced (Fig. 30A)
apical outgrowth of basoposterior process	margin smooth or weakly dentate; lamellar part moderately developed (Fig. 24D)	margin pronouncedly dentate; lamellar part strongly developed (Figs 26D, 27D–G)	margin pronouncedly dentate; lamellar part moderately developed (Figs 29D, 30B–E)
mesomeroidal lobe	without distinct apicolateral part (Fig. 24F)	with distinct apicolateral part (Fig. 27E, F)	without distinct apicolateral part (Figs 29D, 30C, E)
solenomere	apically with two slender and strongly diverging branches (Fig. 24C–E)	apically clavate, with two contiguous, irregularly shaped, apical lobes (Figs 26D, 27E)	apically with two slender, tightly contiguous branches (Fig. 30D)

#### Descriptive notes.

**Figure 24.**
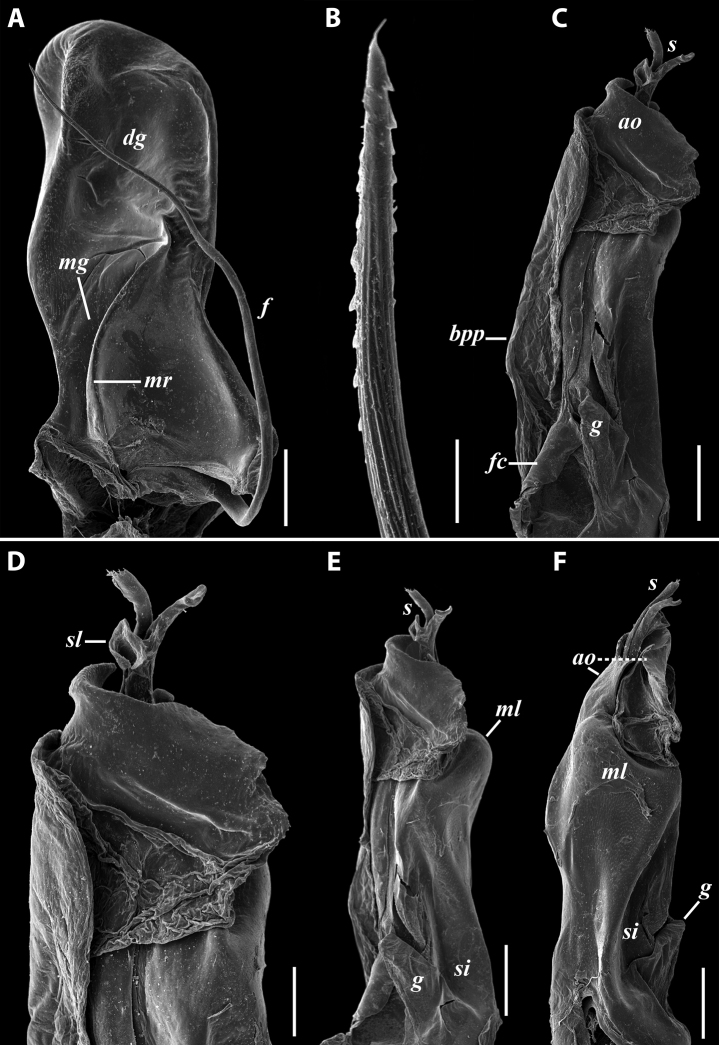
*Omobrachyiulusimplicitus* (Lohmander, 1936), gonopods of ♂ from Myussera, AR Abkhazia, Georgia (ZMUM) **A** right promere, caudal view **B** apical part of flagellum **C** right opisthomere, mesal view **D** distal part of the same aspect **E** right opisthomere, oral-mesal view **F** same, oral view. Scale bars: 0.1 mm (**A, C, E, F**), 0.05 mm (**D**), 0.01 mm (**B**). Abbreviations: ***ao*** apical outgrowth of basoposterior process, ***bpp*** basoposterior process, ***dg*** distal groove, ***dl*** distolateral lobe, ***f*** flagellum, ***fc*** flagellum channel, ***g*** (supposed) gonocoxal gland, ***mg*** median groove, ***ml*** mesomeroidal lobe, ***mr*** median ridge, ***s*** solenomere, ***si*** anteromesal sinus.

Promere (Fig. 24A) slightly sigmoid, of more or less same width all along, ending in a thick and broadly rounded apex; median ridge strongly pronounced and arched, median groove deep and narrow, distal groove deep and broad; flagellum slightly longer than height of promere, apically sparsely micro-dentate (Fig. 24B). Opisthomere (Fig. 24C–F) rather slender; basoposterior process weakly pronounced, lamellar, ending with a collar-like apical outgrowth having a rather small, wrinkled, lamellar part; mesomeroidal lobe moderately pronounced, with a concave apical margin; mesal side with a stout lobe (presumably gonocoxal gland) and a rather deep anteromesal sinus; flagellum channel with only a few minute spiniform filaments; solenomere long and slender, apically forked into two diverging, somewhat flattened branches of nearly equal size and shape; a minute, ear-shaped lobe mesally at the base of the bifurcation.

#### Previous records from the Caucasus.

Georgia, AR Abkhazia, Gagry (type locality), near Lake Ritsa (the type locality of *O.i.ritsensis*)

#### General distribution.

WECA.

#### Remarks.

The species is new to the fauna of Russia.

The subspecies *O.i.ritsensis* was described by Golovatch (1981) from the environs of Lake Ritsa, AR Abkhazia. The author listed the sparser metazonal striations, the slightly upwards bent epiproct and the different shape of the promere as the main diagnostic characters that distinguish *ristensis* from the typical form. The gonopods of all examined males, including the topotypic material from north of Lake Ritsa, show no significant differences from each other and they all match well the original descriptions and drawings of both the typical *O.implicitus* and *O.i.ritsensis*. On the other hand, the two aforementioned external somatic characters cannot alone reliably delimit a subspecies, as in Julidae they normally display some variations even within one population. We thus formally synonymise *O.implicitusritsensis* with the typical *O.implicitus*, syn. nov.

### Omobrachyiulus fasciatus

Taxon classificationAnimaliaJulidaJulidae

Vagalinski sp. nov.

902B73BE-7461-58FC-BBF5-A319018CC993

http://zoobank.org/168675F9-3F8B-4028-B5F5-A269337E6DF7

Figs 25 , 26 , 27

#### Material examined

**(all from Russia). *Holotype***: ♂ (in two pieces, gonopods dissected, right antenna missing) (ZMUM), Republic of Adygea, Caucasian Nature Reserve, Lagonaki Plateau, Azishskiy Pass, forest, pitfall traps, 14–26.VIII.2013, Yu. Chumachenko leg. ***Paratypes***: 1 ♂, 1 ♀, 1 subad. ♂, 2 juv. ♀♀ (SMNG 34136) (the male in 2 pieces, with dissected gonopods; the female unbroken, the subadult in two pieces, the juveniles unbroken), Republic of Adygea, Mt. Koryto, by a hut, 44°03'53"N, 40°21'02"E, 1600 m a.s.l., open *Abies* forests with dense undergrowth of herbs and shrubs, sifting leaf litter, 22.VIII.2005, K. Voigtländer leg.; 1 ♂, 1 ♀ (SMNG 34142) (the male in 2 pieces, with dissected gonopods; the female in two pieces, right vulva dissected), Republic of Adygea, Shestakova Meadow, 44°01'56"N, 40°23'25"E, *Abies*, *Acer*, ferns, etc., pitfall traps, 24–25.VIII.2005, K. Voigtländer leg.; 1 ♂, 1 juv. ♀ (SMNG 34154) (the male in head to ring 6 and rest of body, gonopods and hypoproct dissected; the juvenile unbroken), Republic of Adygea, a hut on the west bank of Afonka River, 44.0019°N, 40.4066°E, at a tree base, hand collecting, 27.VIII.2005, K. Voigtländer leg.; 1 ♂ (NMNHS) (in head to ring 6, ring 7, and rest of body in three pieces; gonopods and hypoproct dissected), at Sakhray River, some 2 km E of Novoprokhladnoye, 44°08'11.4"N, 40°19'04.4"E, 635 m a.s.l., *Fagus* forest, in dead wood, hand collecting, 30.VIII.2005, K. Voigtländer leg.; 2 ♂♂ (NHMD) (one in 2 pieces, gonopods, dissected, the other in 2 pieces, gonopods missing), 5 ♀♀ (NHMD) (some partly damaged), 3 juv. (NHMD), Krasnodar Province, ca. 9 km SW of Mount Fisht, Babuk-Aul, 43°53'26"N, 39°49'11"E, 560 m a.s.l., *Castanea* and *Fagus* forest, sifting leaf litter, 11.VII.2011, A. Solodovnikov leg.; 2 ♂♂ (NHMD) (one unbroken, the other in five pieces, antenna, penis, leg 3, left flange of pleurotergum 7, and hypoproct dissected, opisthomeres and left promere prepared for SEM), 17 ♀♀ (NHMD) (some partly broken, one with dissected vulvae), 28 juv. and subad. (NHMD), Russia, Krasnodar Prov., ca. 7 km SW of Mount Fisht, 43°54.214'N, 39°50.507'E, 1200 m, *Fagus* and *Rhododendron* forest, sifting leaf litter, 14.VII.2011, A. Solodovnikov leg.

#### Diagnosis.

A species of *Omobrachyiulus* most similar to *O.implicitus* and *O.lazanyiae* sp. nov. by the overall shape of the promere, the weakly developed basoposterior process and mesomeroidal lobe of the opisthomere, and the broad, collar- or scarf-shaped, apical outgrowth of the basoposterior process which has a characteristic, wrinkled, lamellar part and partially covering the mesal side of the solenomere. Differs from these two species mainly by the apically clavate solenomere, and by the apical outgrowth of the opisthomeral basoposterior process consisting of a relatively narrow cockscomb-like distal part and a strongly developed scarf-shaped mesal part; as well as by other morphological details summarised in Table 1.

#### Name.

From the Latin *fascia*, meaning scarf or band, after the strongly developed lamellar part of the apical outgrowth of the basoposterior process of the opisthomere, which is wrapped around the basomesal part of the solenomere. Adjective.

#### Description.

***Measurements*:** holotype ♂ in S IX, 45+3+T, L = 15.5 mm, H = 1.05 mm; paratype ♂♂ in S VIII–IX, 44–46+1–3+T, L = 14–16 mm, H = 1–1.1 mm; paratype ♀♀ in S VIII–X, 41–46+2–3+T, L = 11.9–19.9 mm, H = 0.85–1.3 mm.

**Figure 25.**
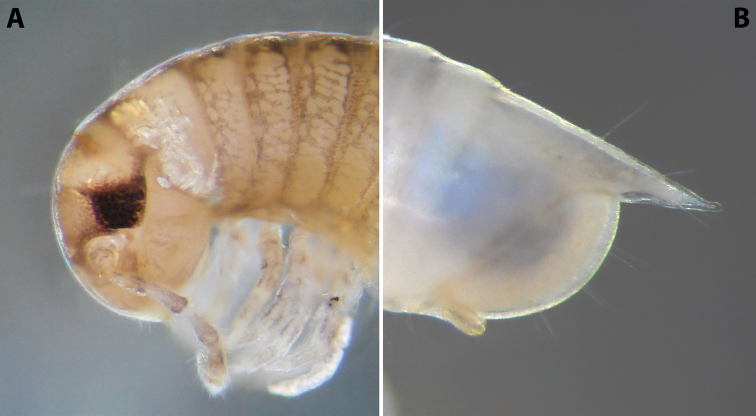
*Omobrachyiulusfasciatus* sp. nov., ♂ holotype **A** head and body rings 1–4 **B** telson, lateral views. Not to scale.

***Colouration*** (Fig. 25A): mostly brown, dorsum dark brown, prozonae dorsally with two short, transverse, blackish stripes, one each at frontal margin and near suture; metazonae with one longer, brown, transverse stripe, gradually narrowing and disappearing somewhat below ozopore level; dorsum with a blurred, blackish, axial line, this being more prominent on prozonae; with a dark brown spot above and in front of ozopore.

***External structures*:** Eye patches in adults consisting of 30–40 ommatidia, mostly arranged in easily countable vertical rows. Vertigial, supralabral, and labral setae: two, four (one ♂ with five), and 16–18, respectively. Antennae 1.3–1.4 × as long as head in males and 1.2–1.3 × in females; antennomere 2 > 4 = 5 ≥ 3. Gnathochilarium with a promentum separating both lamellae linguales over ca. 2/5 of their length, each latter with three or four setae in a longitudinal row; male stipites parabasally each with a longitudinal row of several short and stiff setae. Collum mostly smooth, with two or three broad shallow grooves near posterolateral corners, and with several densely set, oblique, shallow striae laterally at anterior margin.

Body rings barely vaulted. Prozonae with very short, fine, mostly parallel, longitudinal striae near pro-metazonal suture. Metazonae moderately deeply striated, n*_Schub_* = 8 or 9; metazonal setae 1/3 to nearly half of metazonal length. Ozopores set tightly behind pro-metazonal suture in more anterior rings, gradually moved further backwards, to nearly equal to their diameter behind the suture in caudalmost rings, sutures not being sinuous in front of ozopores. Tarsus of mid-body legs equal to, to slightly longer than, tibia, and nearly 3 × as long as apical claw.

**Figure 26.**
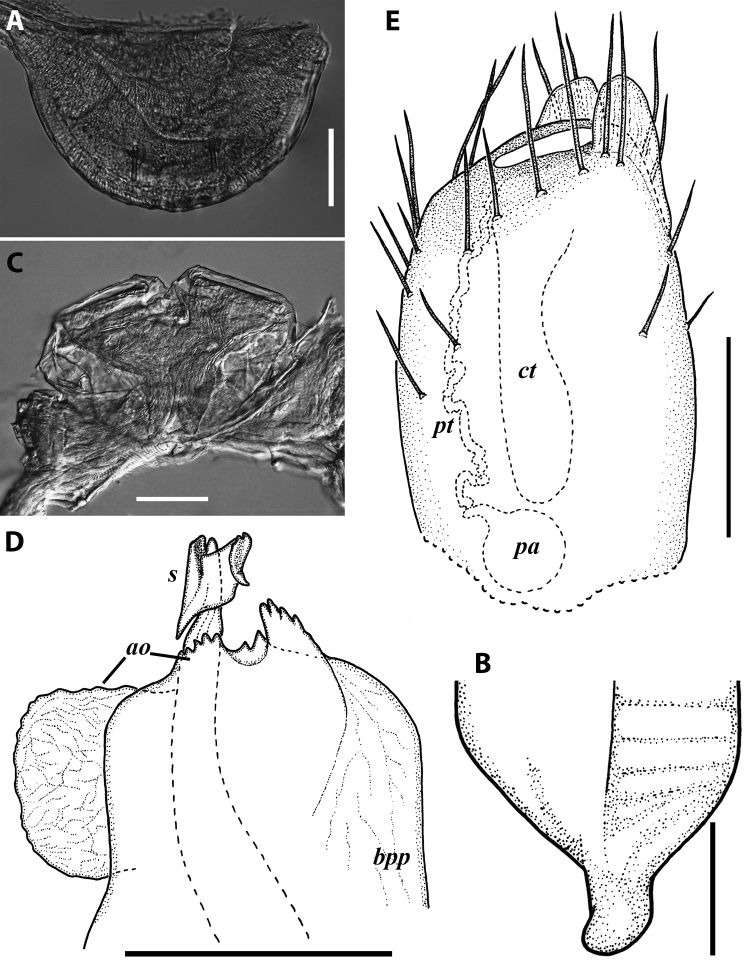
*Omobrachyiulusfasciatus* sp. nov., ♂ (**A–D**) and ♀ (**E**) paratypes from near Mount Fisht, Krasnodar Prov., Russia (ZMUC) **A** hypoproct, ventral view **B** left flange of pleurotergum 7, ventro-lateral view **C** penis, caudal view **D** distal part of right opisthomere, lateral, somewhat oral view **E** right vulva, mesal view. Scale bars: 0.2 mm (**B, E**), 0.1 mm (**D**), 0.06 mm (**A, C**). Abbreviations: ***ao*** apical outgrowth of basoposterior process, ***bpp*** basoposterior process, ***ct*** central tube, ***pa*** posterior ampulla, ***pt*** posterior tube, ***s*** solenomere.

***Telson*** (Fig. 25B): Epiproct long, wedge-like, with a well-developed, slightly clavate, hyaline tip turned dorsad, considerably surpassing longest paraproctal setae. Hypoproct (Fig. 26A) rounded subtrapezoidal to semi-circular, ventrally with two distal paramedian setae; somewhat protruding past caudal margin of paraprocts in males, tightly fitting to their ventral side in females. Paraprocts sparsely to moderately setose.

***Male sexual characters*:** Mandibular stipites weakly to moderately expanded, forming a blunt rectangular or slightly obtuse anterior corner. Leg pair 1 rather slender, parallel to slightly converging hooks. Adhesive leg pads large and crested, gradually reduced in caudal direction, postfemoral ones disappearing in caudal third of body, tibial ones discernible until caudalmost leg pairs; femora without modifications. Pleurotergum 7 ventrally forming rather elongated, distally somewhat clavate and broadly rounded lobes (Fig. 26B) originating from the zone around pro-metazonal suture. Penis (Fig. 26C) very short, with short diverging apical lobes and broad terminal lamellae directed caudad.

**Figure 27.**
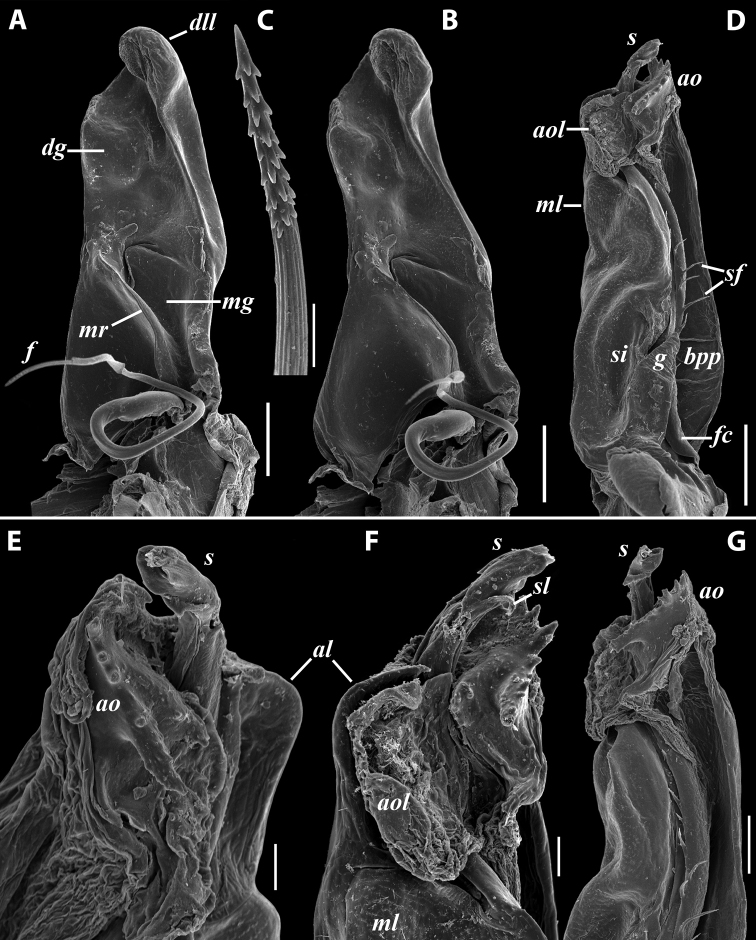
*Omobrachyiulusfasciatus* sp. nov., gonopods of ♂ paratype from near Mount Fisht, Krasnodar Prov., Russia (NHMD) **A** left promere, caudal, somewhat lateral view **B** same, caudal view **C** apical part of flagellum **D** left opisthomere, mesal view **E** distal part of right opisthomere, caudo-mesal, somewhat apical view **F** distal part of left opisthomere, oral-mesal view **G** left opisthomere, caudo-mesal view. Scale bars: 0.1 mm (**A, B, D**), 0.05 mm (**G**), 0.02 mm (**E, F**) 0.01 mm (**C**). Abbreviations: ***ao*** apical outgrowth of basoposterior process, ***aol*** lamellar part of basoposterior process’ apical outgrowth, ***bpp*** basoposterior process, ***dg*** distal groove, ***dl*** distolateral lobe, ***f*** flagellum, ***fc*** flagellum channel, ***g*** (supposed) gonocoxal gland, ***mg*** median groove, ***ml*** mesomeroidal lobe, ***mr*** median ridge, ***s*** solenomere, ***si*** anteromesal sinus.

***Gonopods*** (Figs 26D, 27): Promere (Fig. 27A, B) rather slender, subequal in height to opisthomere, broadest at base, gradually tapering towards a narrow apex bent mesad; caudal surface with a strongly developed median ridge, and a very deep and relatively narrow median groove; with a small and not too deep distal groove, and a thick, strongly pronounced, distolateral lobe; flagellum (Fig. 27C and f in Fig. 27A) slightly longer than height of promere, apically with several whorls of basad pointing denticles. Opisthomere (Figs 26D, 27D–G) rather elongated; a basoposterior process present as a long and narrow, weakly developed lobe, distally forming a dentate cockscomb-like apical outgrowth, mesally extending into a strongly developed, soft, wrinkled, lamellar part, tightly enveloping the base of solenomere mesally and partly frontally; an anterior process absent; a mesomeroidal lobe barely discernible as a weakly developed rounded swelling slightly above opisthomeral mid-height, with a slender apicolateral part bent strongly mesad, considerably outreached by solenomere; mesal side with a large conoid lobe (presumably gonocoxal gland), and a not too deep anteromesal sinus frontal to the gland; several very long, erect and spiniform filaments parabasally along flagellum channel; solenomere relatively slender, bent caudomesad; apex somewhat clavate, consisting of two tightly contiguous, irregularly shaped, apical lobes; a minute tapering lobe bent basad and positioned subapically on mesal side.

***Female sexual characters*:** Leg pairs 1 and 2 thicker and considerably shorter than following legs. Vulva (Fig. 26E) symmetrical, strongly compressed on sides; bursa with an obtuse postero-apical margin; operculum as high as bursa; setation sparse, present only in distal parts of bursa and operculum. Receptaculum seminis consisting of a rather narrow and short central tube, and a very narrow and short, mostly straight, posterior tube leading to a minute, ovoid, posterior ampulla.

#### General distribution.

NWGC.

#### Remark.

The legs on body rings 2–6 of the paratype male from NMNHS were densely overgrown with fungi.

### Omobrachyiulus lazanyiae

Taxon classificationAnimaliaJulidaJulidae

Vagalinski sp. nov.

8D3A3547-0ECB-560C-AEFE-46BE165E424C

http://zoobank.org/FC2D5C93-5638-4EFD-83E6-202BD97D03E4

Figs 28 , 29 , 30

#### Material examined

**(SMNG). *Holotype***: ♂ (in two pieces, gonopods, leg 6 and a mid-body leg dissected), Georgia, AR Abkhazia, Gulripsh Distr., 6 km E of Tsebelda, right bank of Jampali River, Cave Nizhnyaya Shakuranskaya, 8–9.XI.1987, N. Myuge leg. ***Paratype***: 1 ♂ (in head, collum, rings 2–3, ring 4, rings 7–8, and rest of body; leg pair 1, leg 7, a mid-body leg, right flange of ring 7, and hypoproct dissected; opisthomeres and one promere prepared for SEM), same collecting data as for holotype.

#### Diagnosis.

A species of *Omobrachyiulus* most similar to *O.implicitus* and *O.fasciatus* sp. nov. by the overall shape of the promere, the weakly developed basoposterior process and mesomeroidal lobe of the opisthomere, and the broad, collar- or scarf-shaped, apical outgrowth of the basoposterior process, having a characteristic, wrinkled, lamellar part, partially covering the mesal side of the solenomere. Differs from those two species mainly by the solenomere which is apically slender (not clavate as in *O.fasciatus* sp. nov.), divided in two parallel, tightly contiguous (not strongly diverging as in *O.implicitus*) branches, subapically on mesal side bearing a minute lobe with several denticles, protruding perpendicular to the main axis of the solenomere.

#### Name.

Honours the friend and long-term collaborator of the first author, Eszter Lazányi, a diplopodologist from the Hungarian Natural History Museum, Budapest, Hungary, whose scientific contributions mostly focus on the tribe Brachyiulini as well.

#### Description.

***Measurements*:** holotype ♂ in S IX, 41+2+T, L = 12 mm, H = 0.95 mm; paratype ♂ in S IX, 42+1+T, L = 12.5 mm, H = 0.9 mm.

**Figure 28.**
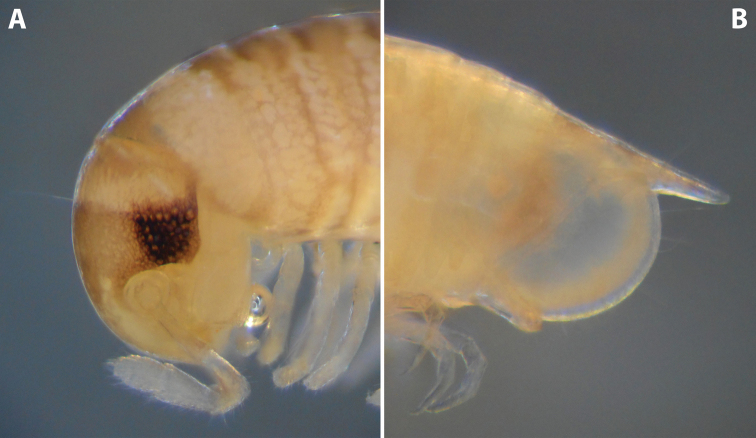
*Omobrachyiuluslazanyiae* sp. nov., ♂ holotype **A** head and body rings 1–3, lateral, slightly dorsal view **B** telson, lateral view. Not to scale.

***Colouration*** (strongly faded from the ethanol conservation) (Fig. 28A): mostly brownish beige; head and collum with the usual pattern; prozonae dorsally with two dark transverse stripes, one in frontal section and the other next to pro-metazonal suture, the former broader; a dark washed spot in front of ozopores; metazonae uniformly light brown-beige; dorsum with a blackish axial line.

***External structures*:** Eye patches in adults consisting of 20–30 ommatidia arranged in unclear vertical rows. Vertigial, supralabral, and labral setae: two, four (one ♂ with five), and 16, respectively. Antennae (in Fig. 28A) 1.1–1.2 × as long as head in males; antennomere 2 > 3 ≥ 4 = 5. Gnathochilarium with a very small promentum separating both lamellae linguales over ca. 1/4 of their length, each latter with four setae in a longitudinal row. Collum completely smooth, with just one or two faint longitudinal grooves near posterolateral corners.

Body rings not vaulted. Prozonae with very short, shallow, scattered, longitudinal striae in posterior parts. Metazonae moderately deeply striated, n*_Schub_* = 7 or 8; metazonal setae from 1/2 (in frontal and mid-body rings) to 2/3 (in caudalmost rings) of metazonal length. Ozopores set tightly behind pro-metazonal suture in more anterior rings, moved slightly backwards, to ca. half their diameter behind the suture in more posterior rings, sutures being gently sinuous in front of ozopores. Tarsus of mid-body legs ca. 1.3 × as long as tibia and ca. 3 × as long as apical claw.

**Figure 29.**
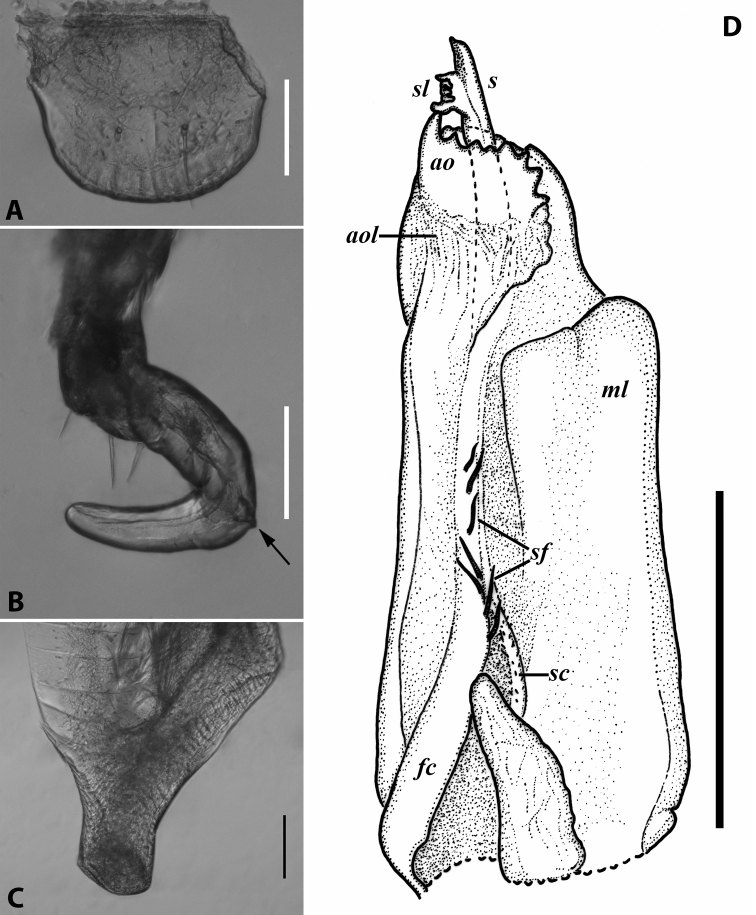
*Omobrachyiuluslazanyiae* sp. nov., ♂ paratype (SMNG) **A** hypoproct (basal corners broken off), ventral view **B** right leg 1, mesal view **C** right flange of pleurotergum 7, ventro-lateral view **D** right opisthomere, mesal, somewhat oral view. Scale bars: 0.2 mm (**D**), 0.1 mm (**A–C**). Abbreviations: ***ao*** apical outgrowth of basoposterior process, ***aol*** lamellar part of basoposterior process’ apical outgrowth, ***fc*** flagellum channel, ***g*** (supposed) gonocoxal gland, ***ml*** mesomeroidal lobe, ***s*** solenomere, ***sc*** seminal channel, ***sf*** spiniform filaments, ***sl*** mesal lobe of solenomere.

***Telson*** (Fig. 28B): Epiproct rather long (approximately reaching the level of longest paraproctal setae), straight, ending bluntly without distinct hyaline tip; bearing several long setae. Hypoproct (Fig. 29A) rounded subtrapezoidal, ventrally with two distal paramedian setae; slightly protruding past caudal margin of paraprocts. Paraprocts sparsely setose.

***Male sexual characters*:** Mandibular stipites (in Fig. 28A) rather weakly expanded, forming a narrowly rounded anterior corner. Leg pair 1 (Fig. 29B) markedly slender, slightly converging hooks with minute pointed tarsal remnants in the paratype (black arrow), the latter being absent in the holotype. Leg pair 2 somewhat shorter than following pairs. Adhesive pads as in *O.fasciatus* sp. nov. Ventral protrusions of pleurotergum 7 (Fig. 29C) similar to those in *O.fasciatus* sp. nov., but insignificantly widening distally.

**Figure 30.**
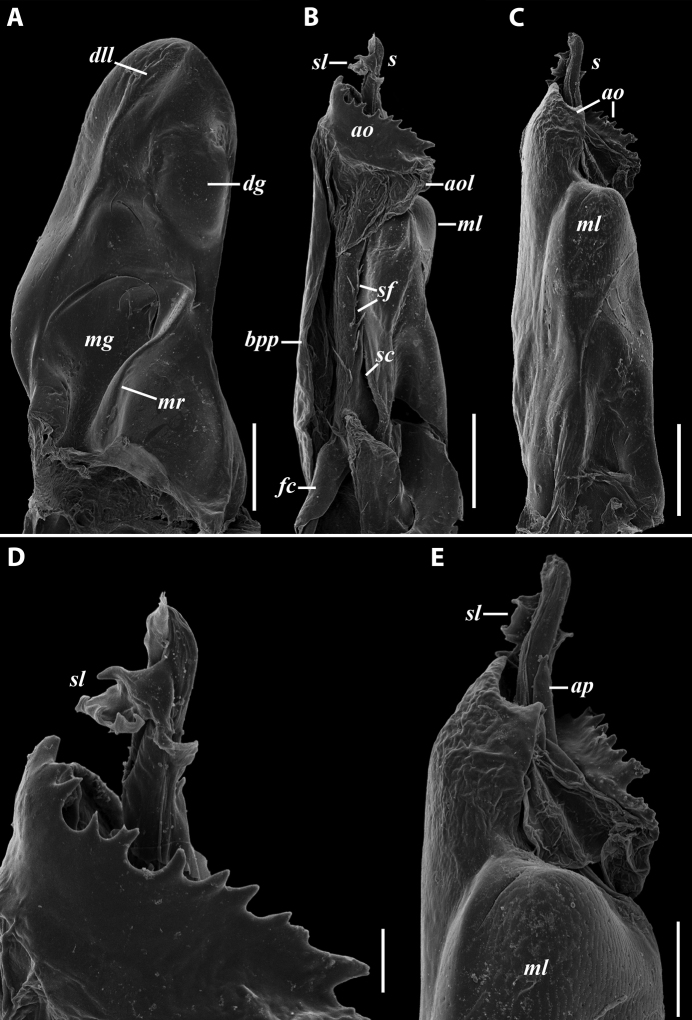
*Omobrachyiuluslazanyiae* sp. nov., gonopods of ♂ paratype (SMNG) **A** right promere (flagellum broken off), caudal view **B** right opisthomere, mesal view **C** left opisthomere, latero-oral view **D** apical part of right opisthomere, mesal view **E** distal part of left opisthomere, latero-oral view. Scale bars: 0.1 mm (**A–C**), 0.05 mm (**E**), 0.02 mm (**D**). Abbreviations: ***ao*** apical outgrowth of basoposterior process, ***aol*** lamellar part of basoposterior process’ apical outgrowth, ***bpp*** basoposterior process, ***dg*** distal groove, ***fc*** flagellum channel, ***g*** (supposed) gonocoxal gland, ***mg*** median groove, ***ml*** mesomeroidal lobe, ***mr*** median ridge, ***s*** solenomere, ***sc*** seminal channel, ***sf*** spiniform filaments, ***si*** anteromesal sinus, ***sl*** mesal lobe of solenomere.

***Gonopods*** (Figs 29D, 30): Promere (Fig. 30A) rather stout, subequal to opisthomere, broadest at base, insignificantly tapering towards a broadly rounded apex; caudal surface with a pronounced median ridge, a deep and broad median groove, a small, but deep distal groove near lateral margin, and a rather weakly pronounced distolateral lobe. Opisthomere (Figs 29D, 30B–E) relatively slender; basoposterior process similar to that in *O.fasciatus* sp. nov., but with a broader apical outgrowth surrounding the proximal part of solenomere from all sides except frontally, and having a less strongly developed lamellar part; a (presumably) anterior process present as a small slender leaf running tightly contiguous with the basal part of solenomere; mesomeroidal lobe moderately pronounced, without an apicolateral part; mesal side mostly as in *O.fasciatus* sp. nov., but with somewhat shorter spiniform filaments at flagellum channel; basal section of seminal channel densely micro-spiculate; solenomere slender, gently sigmoid, directed distad; apex divided into two parallel, tightly contiguous branches, the mesal one bearing a minute lobe protruding meso-caudad, with several denticles at margin.

***Female sexual characters*:** unknown.

#### General distribution.

SWGC.

##### The *roseni* group

**Characterisation.** Both gonopod pairs subequal in height. Promere showing a distinct, deep, distal groove. Opisthomere more or less robust and compact, with a strongly pronounced mesomeroidal lobe, a basoposterior process with a moderately pronounced proximal part in the shape of a lamellar ridge or lobe, ending with an apical outgrowth with a shield-like median part and a more or less pronounced lamellar mesal part turned anteriad, a well-differentiated, freely protruding anterior process of various size and shape, a moderately to very deep anteromesal sinus, a flagellum channel overgrown with rather long and erect spiniform filaments, and a stout, tubular, unipartite solenomere, this being broadened apically and turned more or less anteriad. Vulva with operculum subequal in height to bursa.

**Included species.**

*O.roseni* (Verhoeff, 1921)

*O.faxifer* Vagalinski, sp. nov.

*O.zuevi* Vagalinski, sp. nov.

### Omobrachyiulus roseni

Taxon classificationAnimaliaJulidaJulidae

(Verhoeff, 1921)

05723FAE-CB80-5CE5-A479-BA890DE3DBD4

Fig. 31

Brachyiulus roseni Verhoeff, 1921: 44–45, figs 8, 9.
Chromatoiulus roseni : Lohmander 1936: 148; Lang 1959: 1791.
Omobrachyiulus roseni : Vagalinski and Lazányi 2018: 100.

#### Material examined.

***Syntype*** slide (gonopods, flange of male body ring 7) (ZMB 13622; Verhoeff prep. 3794). **Russia**: Republic of Adygea: 1 ♂ (SMNG 33579), Mount Shibaba, 21.V.2004, K. Voigtländer leg.; 4 ♂♂, 1 ♀ (SMNG 33575), at Sakhray River, 20.V.2004, K. Voigtländer leg.; 2 ♂♂, 5 ♀♀, 7 juv. (SMNG 33576), same collecting data.

#### Diagnosis.

A species of *Omobrachyiulus* morphologically very similar to *O.zuevi* sp. nov. Differing from the latter species by having generally more slender and less strongly embossed gonopods, and by certain details of opisthomere structure, as follows: anterior process well-developed, linguiform, clearly visible from most aspects, vs. the same being vestigial, visible only in oral and oral-mesal views in *O.zuevi* sp. nov.; mesomeroidal lobe with a rather weakly developed apicolateral part, vs. the same being massive, nearly reaching the level of the solenomere in *O.zuevi* sp. nov.; solenomere apically only moderately enlarged, with a soft sigmoid process directed basad, vs. the same ending with a very broad, flower-like apex in *O.zuevi* sp. nov.

#### Descriptive notes.

Length 11–12 mm, height 0.8–0.9 mm. Labral setae: 16. Promentum of gnathochilarium separating both lamellae linguales almost halfway; stipites parabasally each with a row of short, stiff setae, similar to the condition seen in *O.ponticus* sp. nov. Tarsus of mid-body legs somewhat shorter than tibia and ca. 3 × as long as apical claw. Leg pair 1 parallel hooks; ventral lobes of ring 7 protruding entirely from metazona.

**Figure 31.**
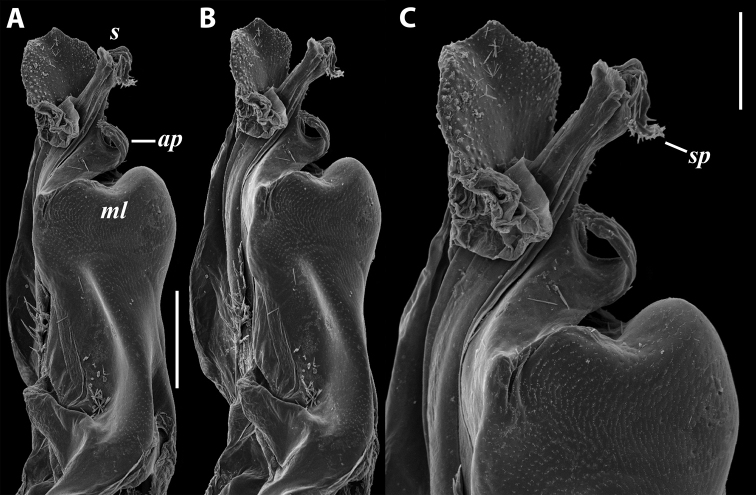
*Omobrachyiulusroseni* (Verhoeff, 1921), gonopods of ♂ from Mount Shibaba, Adygea, Russia (SMNG) **A** right opisthomere, meso-oral view **B** same, oral-mesal view **C** distal part of the same aspect. Scale bars: 0.1 mm (**A, B**), 0.05 mm (**C**). Abbreviations: ***ap*** anterior process, ***ml*** mesomeroidal lobe, ***s*** solenomere, ***sp*** sigmoid process of solenomere.

All remaining external somatic characters as in *O.zuevi* sp. nov.

#### Previous records from the Caucasus.

**Russia**: Republic of Adygea, 40 km NE of Mount Oshten, at an influx of Belaya River [some 20 km S of Maykop] (type locality).

#### General distribution.

WCIS.

#### Remark.

The gonopods of the presently examined near-topotypic males show no differences from those seen on the syntype slide.

### Omobrachyiulus faxifer

Taxon classificationAnimaliaJulidaJulidae

Vagalinski sp. nov.

832812E1-2F0F-5812-80A0-D8C5645CE0F2

http://zoobank.org/7FF26D0F-F0A9-40F4-B4E0-77110770EB04

Figs 32 , 33 , 34

#### Material examined

**(all from Russia): *Holotype***: ♂ (unbroken) (ZMUM), Republic of Adygea, Caucasian Biosphere Nature Reserve, Lagonaki Plateau, Azishskiy Pass, subalpine meadow, pitfall traps, 26.VIII–23.IX.2013, Yu. Chumachenko leg. ***Paratypes***: 1 ♂ (SMNG) (in 2 pieces, with dissected gonopods), Stavropol, Tamanskaya Dacha Forest, near Komsomolskiy Reservoir, 45.0481°N, 41.9567°E, 530 m a.s.l., *Quercus*, *Carpinus*, *Acer*, etc., 22.VIII.2012, F. Walther leg.; 1 ♂ (SMNG 34143) (in 3 pieces, with dissected gonopods), Republic of Adygea, Shestakova Meadow, 44°01'56"N, 40°23'25"E, grassy area with sparse *Fagus* and *Betula* next to a brook, pitfall traps, 24–25.VIII.2005, K. Voigtländer leg.; 2 ♂♂ (ZMUM) (one in 2, the other in 3 pieces, with dissected gonopods), 1 ♂ (unbroken) (NHMD), same collecting data as for holotype; 1 ♂ (ZMUM) (in head to collum and 4 pieces, gonopods and penis dissected), 1 juv. (ZMUM), Republic of Adygea, Caucasian Biosphere Nature Reserve, Lagonaki Plateau, Instruktorskaya Cleft, forest, pitfall traps, 23.VIII–11.IX.2013, Yu. Chumachenko leg.; 2 ♂♂ (ZMUM) (one in two pieces with dissected gonopods, the other in head, collum to ring 2, ring 3 to ring 6, and rest of body, gonopods prepared for SEM), Stavropol, Mamayskiy Forest, 31.III.2013, R. Zuev leg.; 1 ♂ (NMNHS) (in two pieces with dissected gonopods), Stavropol, Tamanskaya Dacha Forest, 10.IX.2013, R.V. Zuev leg.; 1 ♂ (ZMUM) (in 2 pieces with dissected gonopods), 3 adult ♀♀ (ZMUM) (two unbroken, one in 2 pieces with dissected vulvae), 2 subadult ♀♀ (ZMUM), Stavropol, Tamanskaya Dacha Forest, *Quercus*, *Acer*, *Carpinus*, 01.VI.2014, R.V. Zuev leg.

#### Diagnosis.

A species of *Omobrachyiulus* differing from congeners mainly by the apically strongly enlarged and hollow solenomere, postero-apically bearing a fine, pointed, sigmoid process, and by the well-developed lateral lobe of the opisthomere ending with a pointed process which is turned anteriad.

#### Name.

Meaning torch-bearer in Latin, after the resemblance of the solenomere to a burning torch, the flame being represented by a fine apical sigmoid process. Noun in apposition.

#### Description.

***Measurements*:** holotype ♂ in S IX, 42+1+T, H = 1.1 mm, L = 18.5 mm; paratype ♂♂ in S VIII–IX, 40–43+1+T, L = 15–19 mm, H = 1.05–1.3 mm; paratype ♀♀ in S VIII–IX, 42–45+0–1+T, L = 15–21 mm; H = 1.5–1.8 mm.

**Figure 32.**
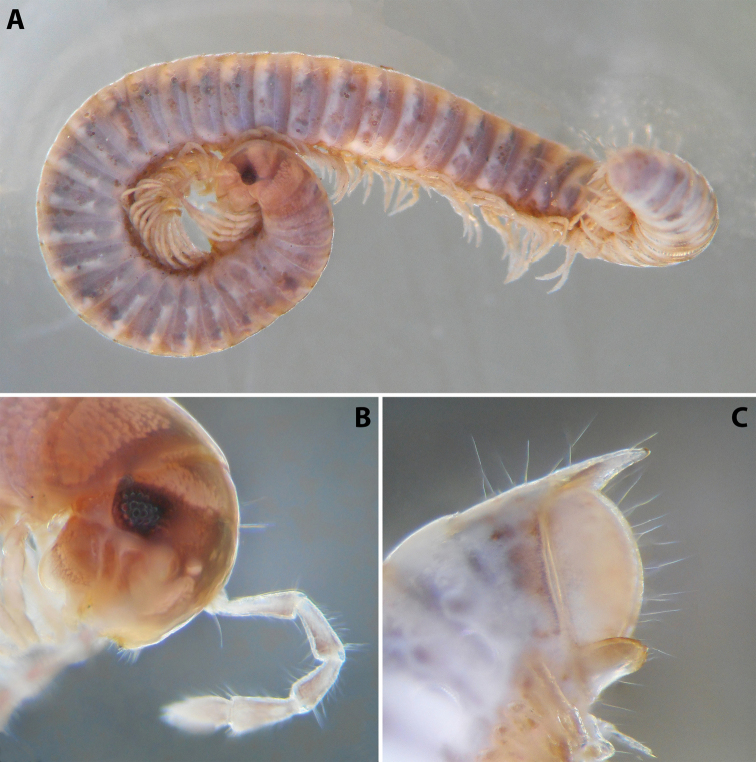
*Omobrachyiulusfaxifer* sp. nov., ♂ holotype (**A**) and ♂ paratype from Lagonaki Plateau, Adygea, Russia (ZMUC) (**B**) **A** habitus, mostly lateral view **B** head and collum, lateral, slightly frontal view **C** telson, lateral view. Not to scale.

***Colouration*** (Fig. 32): Head and collum with the usual pattern; prozonae brown-beige, dorsally with a narrow, dark grey, transverse band near pro-metazonal suture, and with a blackish spot in front of ozopores; metazonae brownish, darker in anterior section, light ochre-brown at hind margin; dorsum with a continuous, blackish, axial line; pre-anal ring dark brown, paraprocts lighter brown-beige.

***External structures*:** Eye patches in adults consisting of 22–35 well-pronounced ommatidia mostly arranged in easily countable vertical rows. Vertigial, supralabral, and labral setae: two, four, and 22–28, respectively. Antennae (in Fig. 32B) 1.6–1.7 × as long as head in males and 1.3–1.4 × in females; antennomere 2 > 5 ≥ 3 ≥ 4 > 6. Gnathochilarium with a relatively large promentum separating both lamellae linguales nearly halfway, each latter with two or three setae in a longitudinal row. Collum mostly smooth, with only tree or four striae near posterolateral corners.

Body rings gently vaulted. Prozonae with very short, fine, mostly purely longitudinal striae in posterior sections. Metazonae rather deeply striated, n*_Schub_* = 6 or 7 in males and 7 or 8 in females; metazonal setae 1/2–2/3 of metazonal length. Ozopores touching or set barely behind pro-metazonal sutures along entire body; sutures not sinuous in front of ozopores. Tarsus of mid-body legs equal to, to slightly longer than, tibia, and ca. 3 × as long as apical claw.

**Figure 33.**
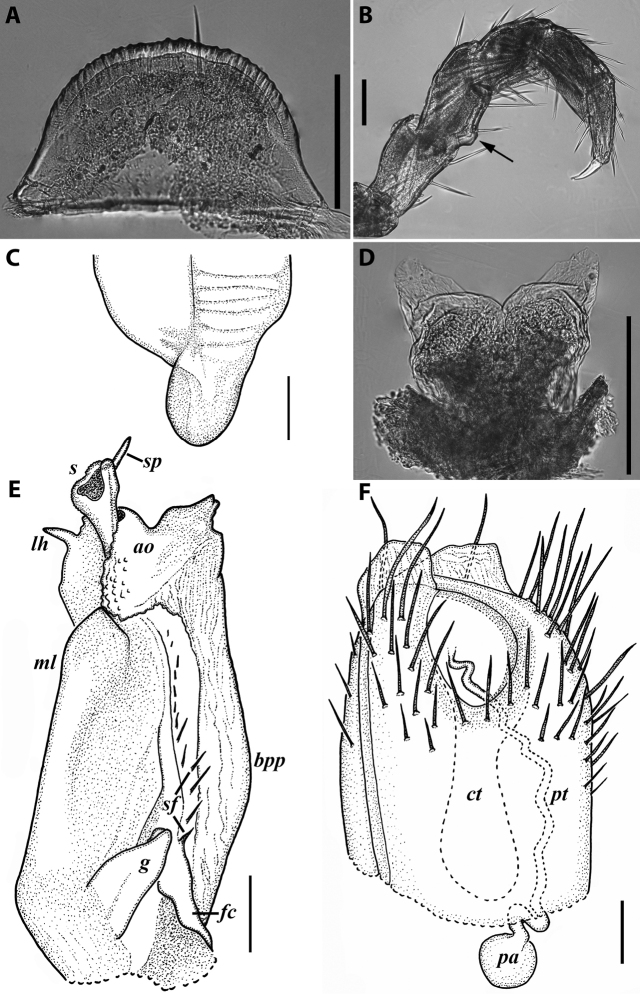
*Omobrachyiulusfaxifer* sp. nov., ♂ paratypes from Lagonaki Plateau, Adygea, Russia (ZMUM) (**A–D**) and Mamayskiy Forest, Stavropol, Russia (ZMUM) (**E**), and ♀ paratype (**F**) from Tamanskaya Dacha Forest, Stavropol, Russia (ZMUM) **A** hypoproct, ventral view **B** leg 2 **C** left flange of pleurotergum 7, ventro-lateral view **D** penis, caudal view **E** left opisthomere, oral-mesal view **F** right vulva, caudal view. Scale bars: 0.2 mm (**A, C, D**), 0.1 mm (**B, E, F**). Abbreviations: ***ao*** apical outgrowth of basoposterior process, ***bpp*** basoposterior process, ***ct*** central tube ***fc*** flagellum channel, ***g*** (supposed) gonocoxal gland, ***lh*** horn-like process of lateral lobe, ***ml*** mesomeroidal lobe, ***pa*** posterior ampulla ***pt*** posterior tube ***s*** solenomere, ***sc*** seminal channel, ***sf*** spiniform filaments, ***si*** anteromesal sinus, ***sp*** sigmoid process of solenomere.

***Telson*** (Fig. 32C): Epiproct mostly straight, distally slightly turned ventrad, wedge- rather than roof-like, ending with an indistinct, blunt, hyaline tip reaching the level of longest anal setae. Hypoproct (Fig. 33A) in males broad trapezoidal with broadly rounded corners, margin coarse, sometimes forming three small blunt denticles, considerably protruding past rear margin of paraprocts; same being broader, with an always smooth margin, barely protruding behind rear margin of paraprocts. Paraprocts covered with relatively sparse long setae; without distinct rows of shorter setae along caudal margins.

***Male sexual characters*:** Mandibular stipites (in Fig. 32B) considerably expanded, forming a distinct, blunt or narrowly rounded, anteroventral corner. Leg pair 1 compact, parallel hooks, slightly turned mesad. Legs 2 (Fig. 33B) and following pairs ventrally with well-developed crested adhesive pads, postfemoral ones vanishing in caudal third of body; femora 2 basoventrally with small bumps (black arrow), femora of other legs without modifications. Pleurotergum 7 ventrally forming broad shovel-like lobes (Fig. 33C) originating mostly from metazona, directed ventromesad, touching one another behind gonopods. Penis (Fig. 33D) nearly as long as broad, compressed antero-caudally, with short diverging apical lobes ending in rather large terminal lamellae directed distad.

**Figure 34.**
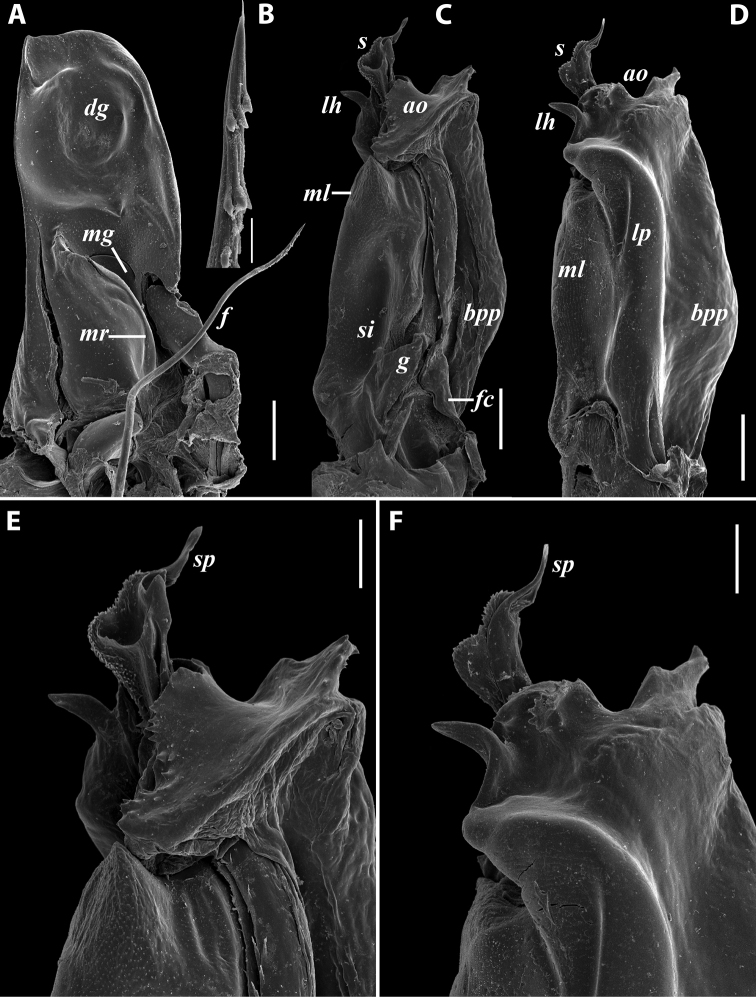
*Omobrachyiulusfaxifer* sp. nov., gonopods of ♂ paratype from Mamayskiy Forest, Stavropol, Russia (ZMUM) **A** left promere (somewhat damaged in proximal part), caudal view **B** apical part of flagellum **C** left opisthomere, mesal view **D** right opisthomere, lateral view **E** distal part of left opisthomere, mesal view **F** distal part of right opisthomere, lateral view. Scale bars: 0.1 mm (**A, C, D**), 0.05 mm (**E, F**), 0.01 mm (**B**). Abbreviations: ***ao*** apical outgrowth of basoposterior process, ***bpp*** basoposterior process, ***dg*** distal groove, ***f*** flagellum, ***fc*** flagellum channel, ***g*** (supposed) gonocoxal gland, ***lh*** horn-like process of lateral lobe, ***lp*** lateral lobe, ***mg*** median groove, ***ml*** mesomeroidal lobe, ***mr*** median ridge, ***s*** solenomere, ***si*** anteromesal sinus, ***sp*** sigmoid process of solenomere.

***Gonopods*** (Figs 33E, 34): Promere (Fig. 34A) as high as opisthomere, broadest at base, abruptly narrowing after an incision at lateral margin’s mid-proximal section; lateral margin distally slightly convex, mesal one very gently sigmoid; apex broad, with a nearly rectangular meso-apical corner; caudal surface with a strongly pronounced median ridge, a deep and very narrow median groove, and a deep, rounded, distal groove; flagellum somewhat longer than height of promere, apically bearing blunt denticles directed basad, arranged in two or three transverse rows. Opisthomere (Figs 33E, 34B–F) moderately elongated; basoposterior process moderately developed, its apical outgrowth large, forming two parts: a smaller median one, and a larger mesal one bent strongly basofrontad, covering the basomesal side of solenomere like a hood; both parts distally with several small conical denticles; an anterior process absent; mesomeroidal lobe well-developed, narrow, ridge-like in its proximal part, distally widening in a pyramidal structure with a distinct apex; lateral side with a well-developed ridge-like lobe apically forming a pointed horn-like process bent frontad; mesal side with a relatively large, subtriangular lobe (presumably gonocoxal gland), and a shallow, indistinct, anteromesal sinus; moderately long, erect, spiniform filaments present over most of flagellum channel; solenomere unipartite, apically clavate and deeply hollow, with a fine sigmoid apical process.

***Female sexual characters*:** Vulva (Fig. 33F) rather short and compact, symmetrical; operculum slightly higher than bursa, both bursa and operculum ending up with broad and rather short hyaline protrusions; setation dense throughout. Receptaculum seminis consisting of a very large, sack-shaped, central tube, and a very long, narrow, somewhat folded, posterior tube leading to a nearly spherical posterior ampulla.

#### General distribution.

NWGC-WCIS.

### Omobrachyiulus zuevi

Taxon classificationAnimaliaJulidaJulidae

Vagalinski sp. nov.

119161A5-4781-55C1-91C0-58D161704B8E

http://zoobank.org/A13AF0F9-DE2A-41E2-9DD3-6CC78DFDDDD0

Figs 35 , 36 , 37

#### Material examined

**(ZMUM). *Holotype***: ♂ (in head to ring 6 and rest of body, opisthomeres dissected), Stavropol, Mamayskiy Forest, 31.III.2013, R. Zuev leg. ***Paratypes***: 1 ♂ (in head to ring 2, ring 3 to ring 6, pleurotergum 7 (broken into two pieces), and rest of body; penis and pleurotergum 7 dissected, gonopods prepared for SEM), 1 ♀ (in head to body ring 2 + rest of body, left vulva dissected), same collecting data as for holotype.

#### Diagnosis.

A species of *Omobrachyiulus* being morphologically very similar to *O.roseni* (Verhoeff, 1921). Differing from the latter species by having generally stouter, more robust gonopods, and by details of the opisthomere, as follows: anterior process minute, vestigial, mostly hidden between base of solenomere and distal part of mesomeroidal lobe, vs. the same being significantly larger, clearly visible from most angles in *O.roseni*; mesomeroidal lobe protruding in a massive, rounded, apicolateral part, and a much smaller mesoapical part, vs. the apicolateral part being much less pronounced, nearly same size as the mesoapical one in *O.roseni*; solenomere with a strongly enlarged, flower-like apex, vs. the same being apically only moderately enlarged, with a soft sigmoid process directed basad in *O.roseni*.

#### Name.

Honours Roman Zuev, a myriapodologist from Stavropol, Russia, and the collector of the material on which the description of this new species is based.

#### Description.

***Measurements*:** holotype ♂ in S VIII or IX, 40+2+T, L = 10.5 mm, H = 0.8 mm; paratype ♂ in S VIII or IX, 42+2+T, L = 12 mm, H = 0.85 mm; paratype ♀ in S IX or X, 44+1+T, L = 14.5 mm, H = 1.15 mm.

**Figure 35.**
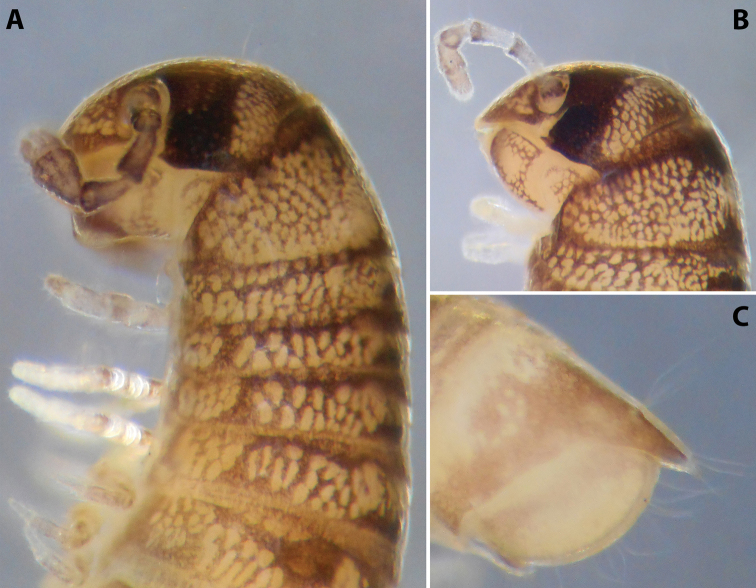
*Omobrachyiuluszuevi* sp. nov., ♂ holotype (**A, C**) and ♀ paratype (**B**) **A** head and body rings 1–5, lateral view **B** head and collum, dorsolateral view **C** telson, lateral view. Not to scale.

***Colouration*** (Fig. 35): Mostly brown-beige, frontal and caudal parts of body darker than mid-body; head with the usual pattern; antennae light brown; collum dark brown at margins; prozonae with numerous densely set yellowish oval spots surrounded by brown rings forming a reticulate pattern; dorsally with a transverse dark brown stripe; metazonae light brown to yellowish beige; whole ventral side of body light beige; dorsum with a dark brown axial line; legs very light, in males dorsally with brown tinges.

***External structures*:** Eye patches in adults consisting of ca. 30 weakly pronounced ommatidia, arranged in hardly recognisable vertical rows. Vertigial, supralabral, and labral setae: two, four, and 13 or 14, respectively. Antennae (in Fig. 35A, B) ca. 1.3 × as long as head in males and 1.15 × in females; antennomere 2 > 3 = 5 > 4 > 6. Gnathochilarium with relatively small promentum separating lamellae linguales in ca. 1/3 of their length, each latter with four setae in a longitudinal row. Collum mostly smooth, with only three or four very shallow, barely visible grooves near posterolateral corners.

Body rings not vaulted. Prozonae with very short and fine, mostly parallel longitudinal striae. Metazonae rather shallowly striated, n*_Schub_* = 6 or 7; setae absent. Ozopores set tightly behind pro-metazonal suture in more anterior rings, gradually moved further back, to nearly equal to their dm behind the suture in caudalmost rings, sutures slightly (in the males) to strongly (in the female) sinuous in front of ozopores in anterior body section. Tarsus of mid-body legs as long as tibia, and slightly > 3 × as long as apical claw.

***Telson*** (Fig. 35C): Epiproct moderately long, stout, turned somewhat ventrad in males, almost perfectly straight in females, ending with a short and blunt hyaline tip, not reaching the level of the longest paraproctal setae; with several long setae. Hypoproct semi-elliptical (more broadly rounded in the female) ventrally with two median submarginal setae, margin somewhat protruding behind rear contour of paraprocts in the males, tightly adhering to their ventral side in the female. Paraprocts sparsely covered with very long setae; no distinct rows of shorter setae along caudal margins.

**Figure 36.**
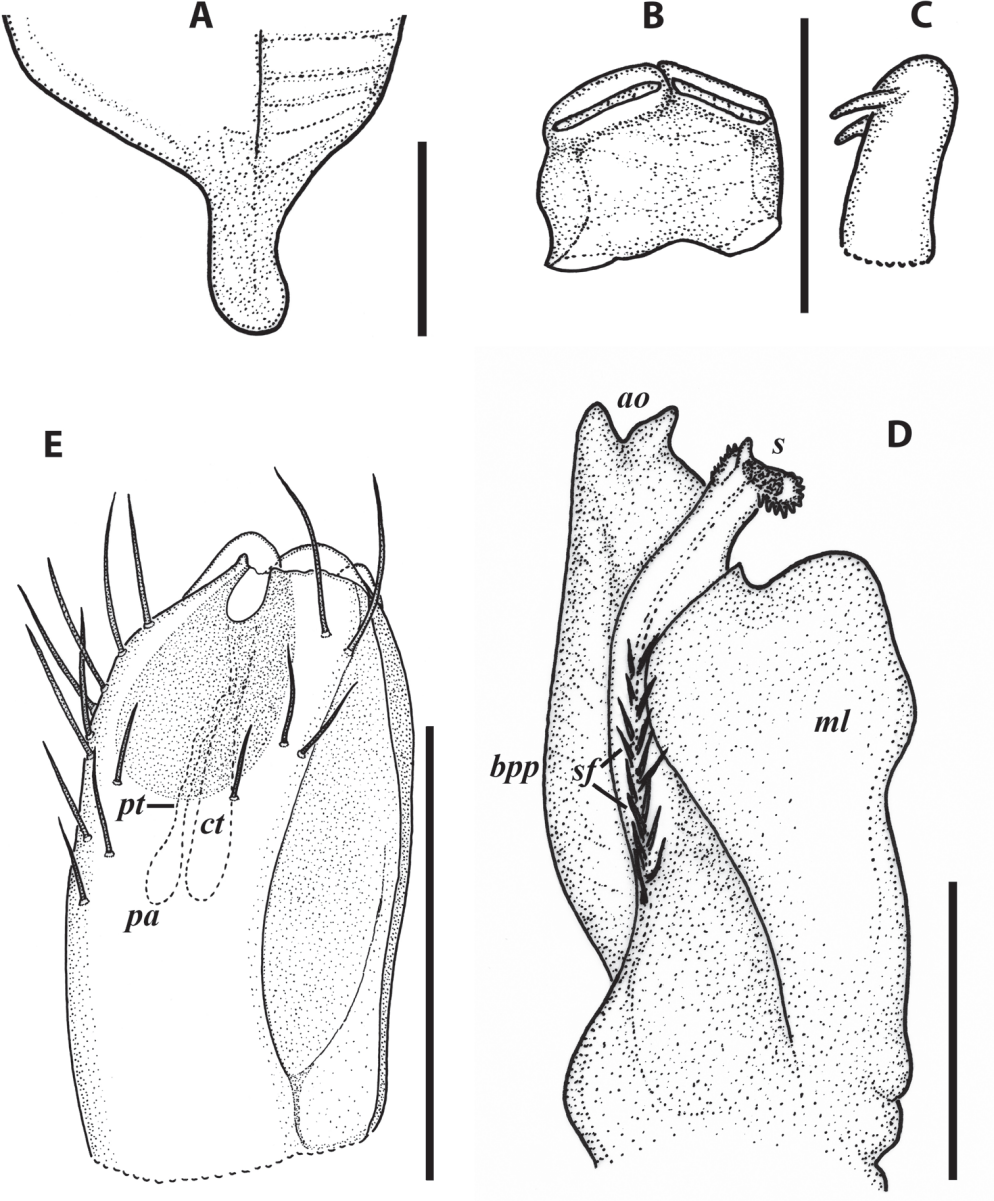
*Omobrachyiuluszuevi* sp. nov., ♂ (**A–D**) and ♀ (**E**) paratypes **A** left flange of pleurotergum 7, ventro-lateral view **B** penis, caudal view **C** same, lateral view **D** right opisthomere, oral-mesal view **E** left vulva, caudal, somewhat lateral view. Scale bars: 0.2 mm. Abbreviations: ***ao*** apical outgrowth of basoposterior process, ***bpp*** basoposterior process, ***ct*** central tube, ***ml*** mesomeroidal lobe, ***pa*** posterior ampulla, ***pt*** posterior tube, ***s*** solenomere, ***sf*** spiniform filaments.

***Male sexual characters*:** Mandibular stipites (in Fig. 35A) moderately expanded, protruding mostly ventrad, forming a rounded anteroventral corner. Leg pair 1 compact hooks turned slightly to considerably against one another. Leg pair 2 and following pairs with crested adhesive pads, postfemoral ones disappearing after mid-body, tibial ones present until caudalmost legs; femora of leg pair 3 and next several pairs with an oval groove. Pleurotergum 7 ventrally forming elongated rounded lobes (Fig. 36A) originating from the zone around pro-metazonal suture, protruding mostly mesad behind gonopods. Penis (Fig. 36B, C) compact, stout, broader than long, with very short, contiguous, apical lobes ending in small terminal lamellae turned caudad.

**Figure 37.**
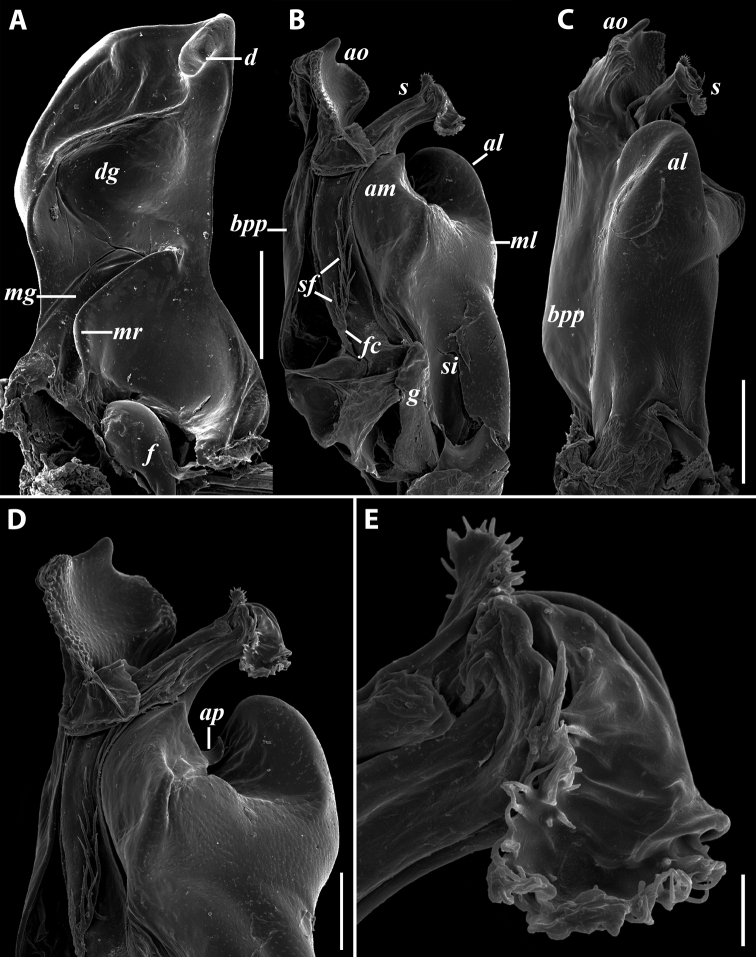
*Omobrachyiuluszuevi* sp. nov., gonopods of ♂ paratype **A** right promere, caudal view **B** right opisthomere, mesal, somewhat oral view **C** left opisthomere, lateral, somewhat oral view **D** distal part of right opisthomere, oral-mesal view **E** apex of solenomere, same aspect. Scale bars: 0.1 mm (**A–C**), 0.05 mm (**D**), 0.01 mm (**E**). Abbreviations: ***al*** apicolateral part of mesomeroidal lobe, ***am*** apicomesal part of mesomeroidal lobe, ***ao*** apical outgrowth of basoposterior process, ***ap*** anterior process ***bpp*** basoposterior process, ***d*** subapical denticle ***dg*** distal groove ***f*** flagellum, ***fc*** flagellum channel, ***g*** (supposed) gonocoxal gland, ***mg*** median groove ***ml*** mesomeroidal lobe, ***mr*** median ridge, ***s*** solenomere, ***sf*** spiniform filaments, ***si*** anteromesal sinus.

***Gonopods*** (Figs 36D, 37): Promere (Fig. 37A) relatively broad and stout, as high as opisthomere, broadest at base, somewhat narrowing in mid-section, then widening again distally, before lateral margin abruptly slanting to join mesal margin in a narrow, blunt apex; caudal surface with a well-pronounced, strongly arched, median ridge, a relatively deep and very narrow median groove, and a broad and deep distal groove; a broad and flat, subapical denticle turned basocaudad; flagellum somewhat longer than height of promere. Opisthomere (Figs 36D, 37B–E) stout and robust; basoposterior process present as a weakly pronounced lobe, distally forming a broad, flattened, shield-like, apical outgrowth protruding in a median, blunt, digitiform tip at margin; anterior process very small, mostly concealed behind distal part of mesomeroidal lobe, the latter massive, very strongly pronounced, distally extending into a broad and rounded apicolateral part, and a much lower, pointed, pyramidal apicomesal part; mesal side with a rather narrow lobe (presumably gonocoxal gland), and a very deep and spacious anteromesal sinus; a row of long and erect spiniform filaments at proximal section of flagellum channel; solenomere unipartite, rather broad tubular, turned anteriad, apically strongly broadening, forming a lamellar flower-like structure with multiple, minute, spiniform filaments at margin.

***Female sexual characters*:** Leg pairs 1 and 2 slightly thicker than following legs. Vulva (Fig. 36E) symmetrical, relatively elongate, somewhat compressed on sides; bursa with a strongly obtuse postero-apical margin; operculum as high as bursa, both parts ending with small hyaline protrusions; setation dense throughout. Receptaculum seminis consisting of a rather broad, digitiform, central tube, and a very narrow, short, mostly straight, posterior tube gradually widening into a small, drop-like, posterior ampulla.

#### Remark.

The complete lack of metazonal setae is a rare condition to be observed in the Brachyiulini: apart from *O.zuevi* sp. nov. and its close sibling, *O.roseni*, this can only be seen in their Aegean congener, *O.strasseri* Vagalinski & Lazányi, 2018.

#### General distribution.

WCIS.

##### The *sevangensis* group

**Characterisation.** Both gonopod pairs subequal in height. Promere lacking a distinct distal groove. Opisthomere rather elongated, with a moderately to strongly pronounced mesomeroidal lobe developed mostly or entirely within the basalmost part of the opisthomere, a basoposterior process with variously pronounced proximal part, ending with a narrow, more or less tapering apical outgrowth turned partly to completely anteriad, a well-developed, ridge-like, at least partly lamellar, anterior process, a variously developed anteromesal sinus, a flagellum channel overgrown with rather short and slanting spiniform filaments, and a more or less slender, uni- or bipartite solenomere directed (almost) completely distad. Vulva with the operculum considerably higher than the bursa.

**Included species.**

*O.sevangensis* (Lohmander, 1932), comb. nov.

*O.kvavadzei* Vagalinski, sp. nov.

*O.ponticus* Vagalinski, sp. nov.

*O.trochiloides* Vagalinski, sp. nov.

### Omobrachyiulus sevangensis

Taxon classificationAnimaliaJulidaJulidae

(Lohmander, 1932) comb. nov.

BD441442-676D-5FF9-87B3-6DC206601CDE

Chromatoiulus sevangensis Lohmander, 1932: 178–180, fig. 9.
Chromatoiulus (Armeniobrachyiulus) sevangensis : Lohmander 1936: 146, figs 123–125; Lang 1959: 1791.
Megaphyllum cf. sevangense : Enghoff 2006: 184.
“Megaphyllum” sevangense: Vagalinski and Lazányi 2018: 121.

#### Material examined.

**Armenia**: Elenovka [Sevan], 2 ♂♂ (ZIN) (gonopods dissected, not in the vial), 4 ♀♀ (vulvae dissected, not in the vial), 10.VIII.1932, A. Schelkovnikov leg.

#### Diagnosis.

A species of *Omobrachyiulus* most simillar to *O.kvavadzei* sp. nov., *O.ponticus* sp. nov., and *O.trochiloides* sp. nov. in gonopod structure, as well as in the shape of the male telson. Differs from these species mostly by details of the opisthomere: from *O.kvavadzei* sp. nov. by the significantly lower position of the mesomeroidal lobe devoid of a distinct apicolateral part; from *O.ponticus* sp. nov. by both the anterior process and the apical outgrowth of the basoposterior process being subequal to, rather than considerably exceeded by, the solenomere; and from *O.trochiloides* sp. nov. by having a much more obscure, poorly differentiated basoposterior process ending in an unciform, rather than a fine rod-like, apical outgrowth.

#### Previous records from the Caucasus.

**Armenia**: Sevan (type locality); ?**Turkey**: vil. Artvin, above Göktaş (Enghoff 2006).

#### General distribution.

LECA.

#### Remarks.

Until now, the systematic position of this species has been doubtful. Lohmander (1936) treated it as the sole member of Armeniobrachyiulus Lohmander, 1936, a subgenus of Megaphyllum (= *Chromatoiulus* auct.). In that same publication he emphasised the similarity of the gonopod conformation in *sevangense* to that of members of *Omobrachyiulus* (at that time a subgenus of *Megaphyllum*), which implied that the two subgenera were closely related. Vagalinski and Lazányi (2018) elevated *Omobrachyiulus* to a full genus and stated that examination of material of *sevangense* was necessary in order to reveal its generic affiliation.

Unfortunately, the presently examined material has previously been dissected and lacks both male and female copulatory organs. However, the newly described species *Omobrachyiuluskvavadzei* sp. nov., *O.ponticus* sp. nov., and *O.trochiloides* sp. nov. undoubtedly belong to the same species group, together with *sevangense*. Thus, the latter is treated here as a member of *Omobrachyiulus*. We refrain from the usage of the subgenus Armeniobrachyiulus for reasons explained in the Remarks section under O.kvavadzei sp. nov.

Given the general trend for narrow endemism within the *sevangensis* group and the big distance between Sevan, Armenia (the type locality of *O.sevangensis*) and the province of Artvin, Turkey, the latter record of “*Megaphyllumcf.sevangense*” (Enghoff, 2006) may also refer to another closely related, yet undescribed species.

### Omobrachyiulus kvavadzei

Taxon classificationAnimaliaJulidaJulidae

Vagalinski sp. nov.

7F9BC6CC-0166-5AA2-9E71-859147F6DC59

http://zoobank.org/0CE7BE2B-7943-4116-BBB2-1C1A5792CC6A

Figs 38 , 39 , 40

#### Material examined

**(all from Georgia). *Holotype***: ♂ (unbroken) (ZMUM), AR Ajara, Kintrishi Nature Reserve, mouth of Khekpara River, 2.X.1984, E. Kvavadze leg. ***Paratypes***: 8 ♂♂ (ZMUM) (five unbroken, one in head to ring 3, ring 4 to pleurotergum 7, and rest of body, with dissected penis and gonopods, one in head to pleurotergum 7 and rest of body, gonopods dissected, one broken in 2 pieces, one in 3 pieces), 1 ♂ (NHMD) (unbroken), 1 ♂ (NMNHS) (unbroken), 1 ♂ (IBER) (unbroken), 8 ♀♀ (ZMUM) (six unbroken,two in 2 pieces, one in head to ring 3 and rest of body, one in 2 pieces, head missing), 1 ♀ (NHMD) (unbroken), 1 ♀ (NMNHS) (unbroken), 1 ♀ (IBER) (unbroken), 2 juv. (ZMUM), same collecting data as for holotype; 1 ♂ (in head to ring 6 and rest of body), 4 ♀♀ (one broken in two pieces, the rest unbroken) (ZMUM), AR Ajara, Kintrishi Reserve, Zeraboseli, 450–600 m a. s. l., deciduous forest, litter and under stones, 13.X.1981, SIG leg.; 4 ♂♂ (one in head to ring 6 + rest of body, gonopods dissected; one broken in two pieces, the rest unbroken), 2 ♀♀ (one broken in two pieces, the other unbroken), 2 juv. (ZMUM), same area, 800 m a. s. l., same date and collector; 1 ♂ (in leg to ring 6 and rest of body, gonopods dissected), 2 ♀♀ (one in 2 pieces, the other unbroken), 1 juv. (ZMUM), AR Ajara, Batumi Botanical Garden, 13.X.1978, SIG leg.; 1 ♂ (ZMUM) (in head to ring 6, pleurotergum 7 to ring 9, and rest of body, opisthomeres and left promere prepared for SEM), AR Ajara, E of Kobuleti, 3 km SE of Chakhati, deciduous forest, near a spring, litter and under stones, 14.X.1981, SIG leg.; 1 ♂ (ZMUM) (in head to pleurotergum 7 and rest of body, gonopods dissected), Samtskhe-Javakheti, 15 km W of Adigeni, *Abieas*, *Picea*, *Fagus*, *Acer* etc. forest, 1500–1700 m, litter, logs, under stones, 14–15.V.1983, SIG leg.

#### Non-types

(ZMUM): 1 ♂, 7 ♀♀ (ZMUM) (all fragmented in 2 or more pieces), Georgia, AR Ajara, Kintrishi Nature Reserve, Zeraboseli, 450–600 m a. s. l., 1–3.VI.1981, SIG and J. Martens leg.

#### Diagnosis.

A species of *Omobrachyiulus* most similar to *O.sevangensis* comb. nov., *O.ponticus* sp. nov., and *O.trochiloides* sp. nov. in gonopod structure, as well as in the shape of the male telson. Differs from all these species mainly by the opisthomere having a bifid anterior process and a better developed, higher mesomeroidal lobe ending with a distinct apicolateral part.

#### Name.

In memory of Eristo Kvavadze (1940–2013), a specialist in earthworms and biological control, the collector of the bulk of the material of this new species.

#### Description.

***Measurements*:** holotype ♂ in S IX, 40+1+T, L = 15.5 mm, H = 1.05 mm; paratypes in S VIII–IX, 39–42+1–2+T, L = 13–16.5 mm and 19–24 mm, H = 1–1.1 mm and 1.4–1.6 mm, in males and females, respectively.

**Figure 38.**
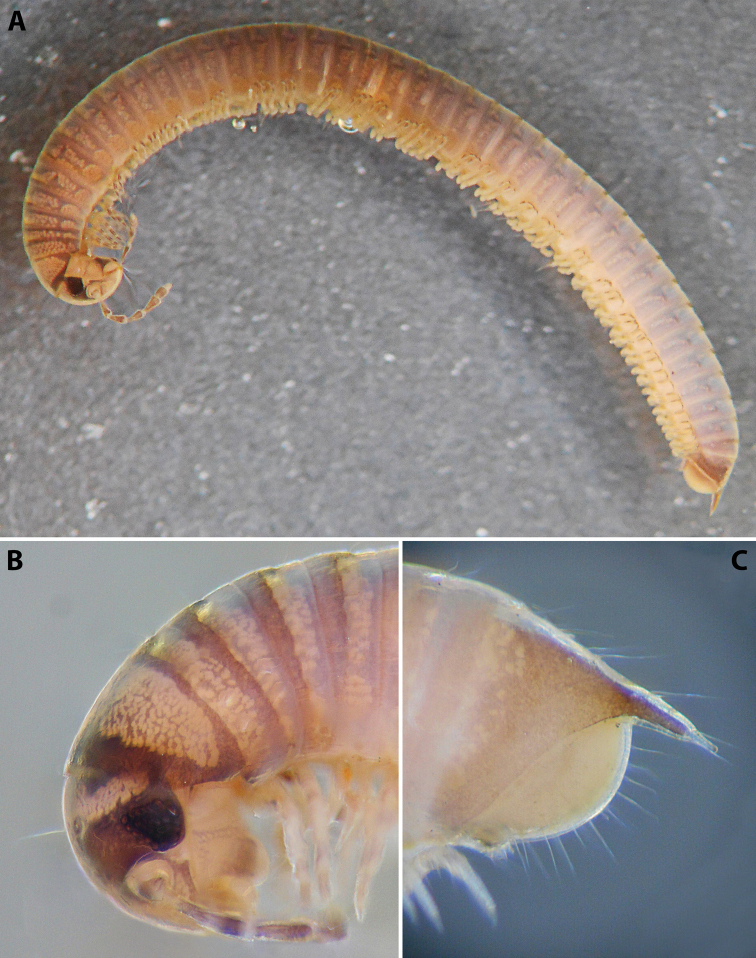
*Omobrachyiuluskvavadzei* sp. nov., ♂ holotype (**A**) and ♂ paratype from near mouth of Khekpara River, AR Ajara, Georgia (ZMUM) (**B, C**) **A** habitus **B** head and body rings 1–4 **C** telson, lateral views. Not to scale.

***Colouration*** (after > 30 years in alcohol) (Fig. 38): Head light brown with the usual dark band between eye patches; collum light brown, margins darker; Prozonae dark brown, becoming greyish on more posterior rings, with numerous lighter spots, dorsally with a blackish transverse stripe; metazonae anteriorly dark brown, lighter at hind margins; dorsum without axial line; whole body lighter on ventral side; pre-anal ring dark brown, paraprocts lighter.

***External structures*:** Eye patches in adults consisting of 30–35 ommatidia arranged in easily countable vertical rows. Vertigial, supralabral, and labral setae: two, four, and 15, respectively. Antennae (in Fig. 38A) ca. 1.5 × as long as head in males and 1.3 × in females; antennomere 5 > 2 > 4 > 3 > 6. Gnathochilarium with promentum almost separating lamellae linguales halfway, each latter with three or four setae in a longitudinal row. Collum smooth, with only several small, shallow, oval grooves near posterolateral corners.

Body rings very gently vaulted. Prozonae with scattered, short and shallow, mostly parallel longitudinal striae. Metazonae relatively deeply striated, n*_Schub_* = 6 or 7 (males) and 7 or 8 (females); metazonal setae ca. 3/4 metazonal length in most rings, this ratio becoming 1:1 in caudalmost rings. Ozopores placed right in pro-metazonal suture in first several rings, gradually taking a more posterior position to ~ 1/2 of their diameter behind suture in caudalmost rings; sutures gently sinuous in front of ozopores in only some rings. Tarsus of mid-body legs 1.3–1.5 × as long as tibia and 3.5–5 × as long as apical claw.

**Figure 39.**
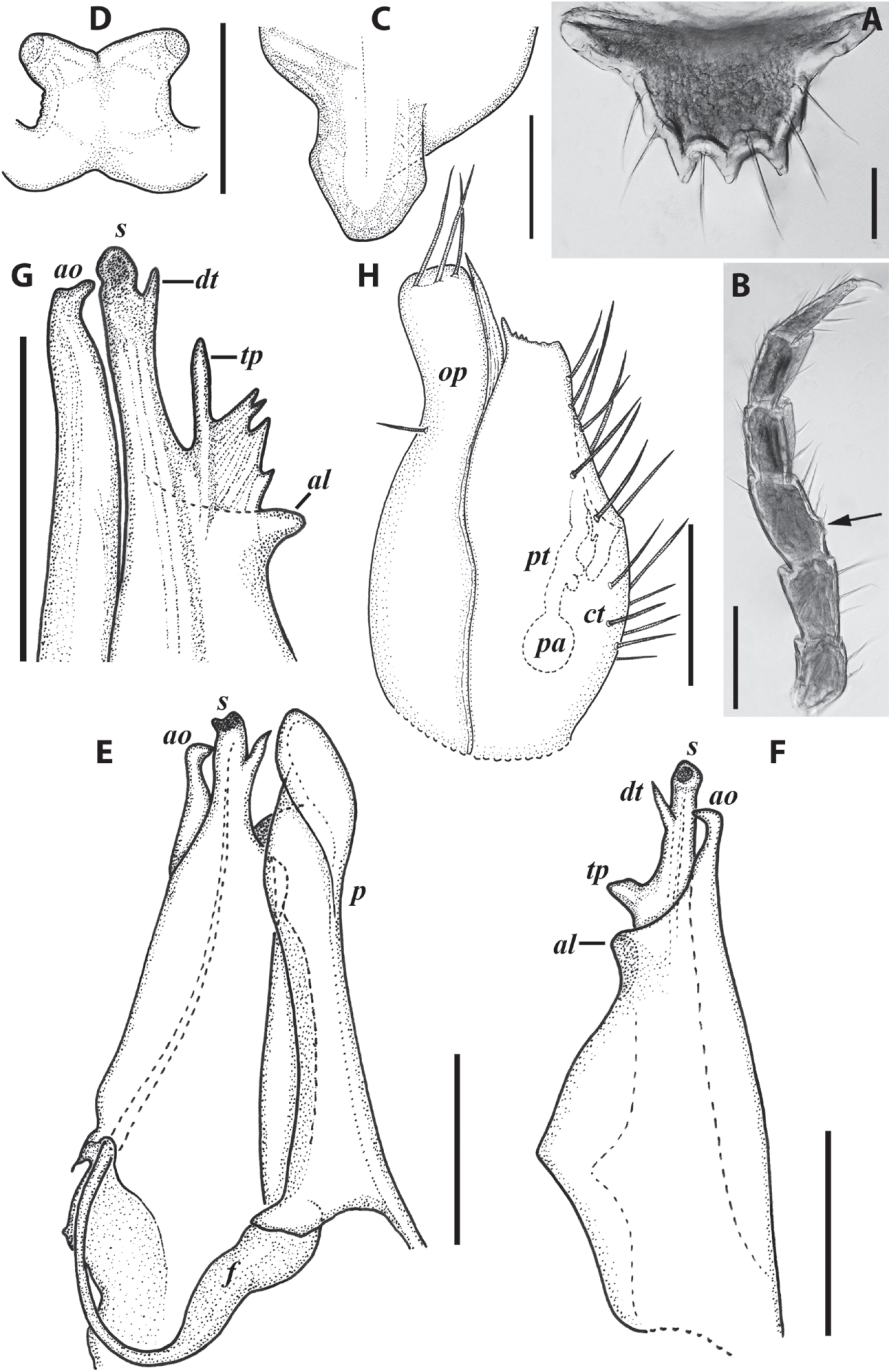
*Omobrachyiuluskvavadzei* sp. nov., ♂ (**A–F**) and ♀ (**H**) paratypes from near mouth of Khekpara River, AR Ajara, Georgia, and ♂ paratype from Zeraboseli, AR Ajara, Georgia (ZMUM) (**G**) **A** hypoproct, ventral view **B** leg 7 **C** right flange of pleurotergum 7, ventro-lateral view **D** penis, caudal view **E** right gonopods, mesal view **F** right opisthomere, caudo-lateral view **G** distal part of right opisthomere, mesal view **H** right vulva, caudo-lateral view. Scale bars: 0.1 mm (**A**), 0.2 mm (**B–H**). Abbreviations: ***al*** apicolateral part of mesomeroidal lobe, ***ao*** apical outgrowth of basoposterior process, ***ct*** central tube, ***dt*** distal tip of anterior process ***f*** flagellum, ***op*** operculum, ***pa*** posterior ampulla, ***pt*** posterior tube ***s*** solenomere, ***sf*** spiniform filaments, ***si*** anteromesal sinus, ***tp*** proximal tip of anterior process.

***Telson*** (Fig. 38C): Epiproct very long, ending in a pointed hyaline tip turned slightly to considerably ventrad, surpassing the longest paraproctal setae in males, just reaching their level in females. Hypoproct (Fig. 39A) relatively small, tridentate in males, with the teeth protruding behind rear contour of paraprocts; rounded, completely concealed under paraprocts in females; ventral surface with six submarginal setae. Paraprocts sparsely to moderately densely setose, without distinct rows of shorter setae along caudal margins.

***Male sexual characters*:** Mandibular stipites (in Fig. 38B) considerably expanded, protruding mostly anteriad, forming a broadly rounded anterior corner. Leg pair 1 compact rounded hooks, somewhat turned against one another. Leg pair 2 and several following pairs (Fig. 39B) with crested adhesive pads, and with a small weakly pronounced bump proximally on femur (black arrow); tibial pads reduced posteriad, but still present until last leg pair, postfemoral ones completely disappearing in last several pairs. Pleurotergum 7 ventrally forming shovel-like lobes (Fig. 39C) originating from the zone around pro-metazonal suture, protruding mostly ventrad, completely concealing opisthomeres from lateral view. Penis (Fig. 39D) antero-caudally strongly compressed, with diverging apical lobes; terminal lamellae not differentiated.

**Figure 40.**
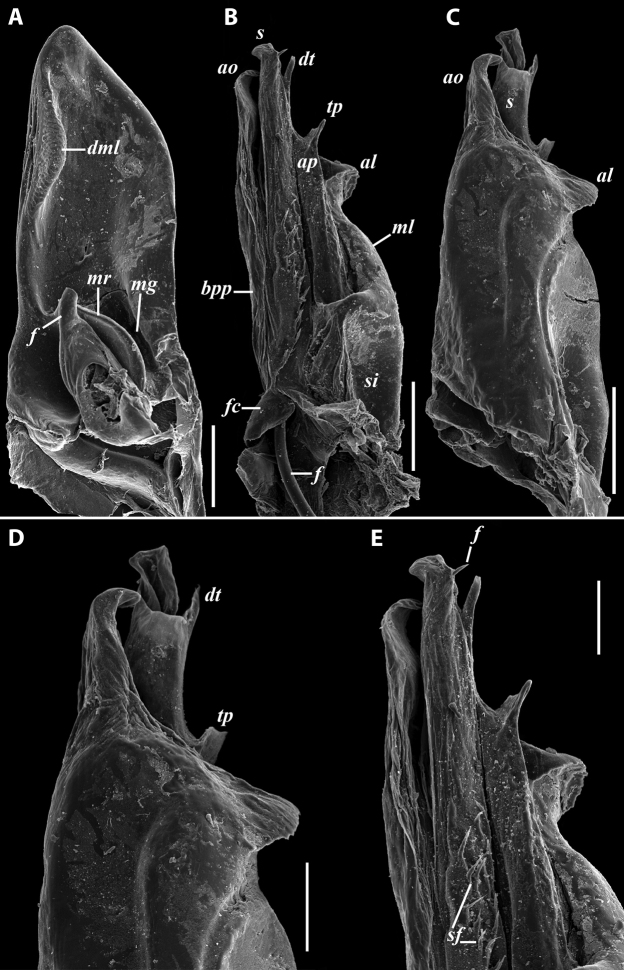
*Omobrachyiuluskvavadzei* sp. nov., gonopods of ♂ paratype from near Chakhati, AR Ajara, Georgia (ZMUM) **A** left promere, caudal view (flagellum broken off near base) **B** right opisthomere (with flagellum in its channel), mesal view **C** left opisthomere, latero-caudal view **D** distal part of the same aspect **E** distal part of right opisthomere (with flagellum tip protruding out of its channel), mesal view. Scale bars: 0.1 mm (**A–C**), 0.05 mm (**D, E**). Abbreviations: ***al*** apicolateral part of mesomeroidal lobe, ***ao*** apical outgrowth of basoposterior process, ***ap*** anterior process, ***bpp*** basoposterior process, ***dml*** distomesal lobe, ***dt*** distal tip of anterior process, ***f*** flagellum, ***fc*** flagellum channel, ***g*** (supposed) gonocoxal gland, ***mg*** median groove, ***ml*** mesomeroidal lobe, ***mr*** median ridge, ***s*** solenomere, ***sf*** spiniform filaments, ***si*** anteromesal sinus, ***tp*** proximal tip of anterior process.

***Gonopods*** (Figs 39G–F, 40A–E): In situ mostly concealed in gonopodal sinus, only apical part of promeres visible, approximately reaching the level of pleurotergal protrusions of pleurotergum 7, opisthomeres fully concealed between promeres and pleurotergal protrusions. Promere (Fig. 40A, p in Fig. 39E) slightly higher than opisthomere, mesal margin mostly straight, lateral margin parallel to the former in proximal section, distally slanting towards a narrowly rounded apex; caudal surface with a short, strongly pronounced, median ridge, a very short and narrow median groove, and a rounded, micro-squamous, distomesal lobe directed caudad; flagellum slightly longer than height of promere. Opisthomere (Figs 39G–F, 40B–E) relatively slender; basoposterior process weakly pronounced, ending in a tapering apical outgrowth with its tip turned anteriad; anterior process shaped as an elongated lobe running parallel to CBO, distally forked into two fine, pointed tips: a shorter, proximal one, and a longer, distal one, the former with a minute lamella at base; mesomeroidal lobe rather weakly pronounced, ending in an apicolateral part with slightly jagged edge bent anteriad; mesal side with a broad and deep anteromesal sinus, and (apparently) without a lobe at base; solenomere unipartite, straight, apically hollow; a row of rather small spiniform filaments along proximal section of flagellum channel.

***Female sexual characters*:** Leg pairs 1 and 2 somewhat thicker than, and ca. as long as, following legs. Vulva (Fig. 39H) relatively elongated; bursa symmetrical, with a strongly obtuse postero-apical margin; side sclerites apically ending up in small hyaline protrusions; operculum significantly higher than bursa, distally thickened and bent somewhat anteriad, with a rounded apical margin, caudal surface laterally ending up with two pointed hyaline protrusions; whole bursa sparsely setose, operculum with only several setae in distal part. Receptaculum seminis consisting of a short finger-shaped central tube, and a mostly straight posterior tube of same gauge, ending in a medium-size ovoid posterior ampulla.

#### Remarks.

As already mentioned under the Remarks section referring to *Omobrachyiulussevangensis* comb. nov., Lohmander (1936) emphasised the peculiar gonopod morphology of that species to justify the erection of the monotypic subgenus Armeniobrachyiulus of the genus *Megaphyllum* (*Chromatoiulus* auctt.) in that same paper. *O.kvavadzei* sp. nov., despite sharing obvious gonopod similarities with *O.sevangensis* comb. nov., differs in having a more vertically developed mesomeroidal lobe and only partly lamellar anterior process of the opisthomere, which sets it closer to the “typical” members of *Omobrachyiulus*. For that reason, we prefer to refrain from the usage of the subgenus Armeniobrachyiulus, and treat *O.sevangensis* and its three most similar congeners under the *sevangensis* species group.

#### General distribution.

LECA.

### Omobrachyiulus ponticus

Taxon classificationAnimaliaJulidaJulidae

Vagalinski sp. nov.

FA33AAF8-20C6-5286-AECF-2C84C63A72A7

http://zoobank.org/5F4E33FA-A9FF-445F-B33A-27BC59971AB5

Figs 41 , 42 , 43

#### Material examined

**(ZMUM). *Holotype***: ♂ (in head, collum to ring 3, ring 4 to ring 6, and rest of body; leg pair 1, penis, right leg 3, gonopods, left flange of pleurotergum 7, and a mid-body leg dissected) (ZMUM), Georgia, AR Abkhazia, Pitsunda-Myussera Nature Reserve, Myussera part, 20–130 m, mixed deciduous forest (*Castanea*, *Alnus*, etc.), litter, under bark and stones, 8–10.IV.1983, SIG leg. ***Paratypes***: 2 ♀♀ (one unbroken, the other in head to ring 3 and rest of body, right vulva dissected), same collecting data as of the holotype.

#### Diagnosis.

A species of *Omobrachyiulus* most similar to *O.sevangensis* (Lohmander, 1936) comb. nov., *O.kvavadzei* sp. nov., and *O.trochiloides* sp. nov.; sharing with O. *sevangensis* and *O.trochiloides* sp. nov. the presence of a lamellar ridge-like anterior process of the opisthomere. Differs from the former species by the presence of a distinct, rounded, apicolateral part of the mesomeroidal lobe of the opisthomere, as well as by the solenomere considerably outreaching both anterior process and apical outgrowth of the basoposterior process, and from the latter species by the much more obscure, poorly differentiated basoposterior process ending in an unciform, rather than a fine rod-like, apical outgrowth. Resembling *O.kvavadzei* sp. nov. mostly by the promere having a distomesal lobe and by the mesomeroidal lobe of the opisthomere bearing an apicolateral part, but differing in other gonopod characters included in the tabular key to the *sevangensis* group (Table 2).

**Table 2.** Key to the species of the *Omobrachyiulussevangensis* group.

Character	*O.sevangensis* comb. nov.	*O.kvavadzei* sp. nov.	*O.ponticus* sp. nov.	*O.trochiloides* sp. nov.
apical outgrowth of basoposterior process	rather slender, distally tapering, pointing disto-frontad	stout, distally tapering, apex abruptly bent frontad (Figs 39G–F, 40B–E)	stout, distally tapering, pointing disto-frontad (Fig. 43A, C–E)	very fine and slender, pointing disto-frontad (Fig. 46B–E)
mesomeroidal lobe	weakly pronounced, without apicolateral part	rather weakly pronounced, with an apicolateral part pointing frontad (Figs 39G, 40B–E)	moderately pronounced, with an apicolateral part pointing frontad (Fig. 43C–E)	strongly pronounced, without apicolateral part (Fig. 46B)
anterior process	well-developed lamellar ridge without free apical part	distally deeply bifurcated, with only a vestigial lamella (Figs 39G, 40B, E)	well-developed lamellar ridge with free apical part (Fig. 43C–E)	well-developed lamellar ridge without free apical part (Fig. 46B–E)
solenomere	rather stout, unipartite, apically shortly bifurcate	rather stout, unipartite, apically hollow (Figs 39G, F, 40C, D)	slender, bipartite (Fig. 43C, E)	slender, unipartite (Fig. 46B–E)

#### Name.

After the Greek name of the Black Sea, Pontos Euxeinos, to emphasise the type locality of the new species at the eastern coast of that sea. Adjective.

#### Description.

***Measurements*:** holotype ♂ in S VII, 35+1+T, L = 10 mm, H = 0.9 mm; paratype ♀♀ in S VII–VIII, 34–35+2+T, L = 11.5–12 mm, H = 1.2–1.35 mm.

**Figure 41.**
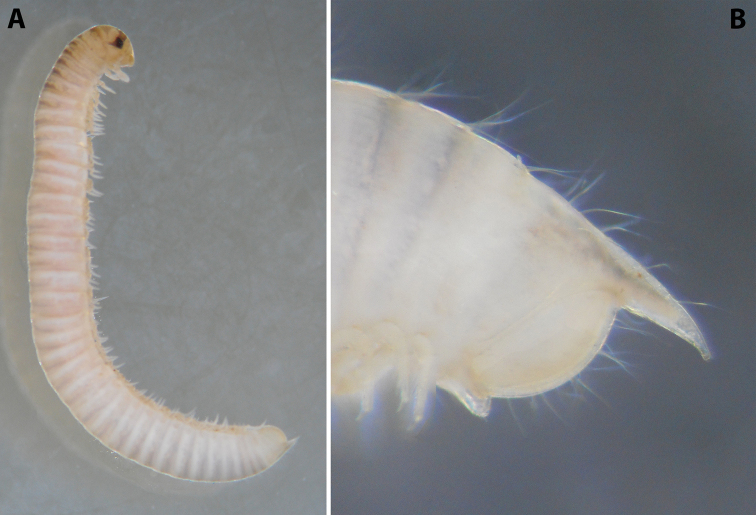
*Omobrachyiulusponticus* sp. nov. **A** habitus of ♀ paratype, lateral, slightly dorsal view **B** telson of ♂ holotype, lateral view. Not to scale.

***Colouration*** (Fig. 41A): Head and collum with the usual colour pattern; body gradually lighter from dorsal to ventral side; prozonae grey, dorsally with a transverse dark brown stripe near pro-metazonal suture; metazonae light brown-beige; dorsum with a continuous whitish axial line.

***External structures*:** Eye patches in adults consisting of 20–23 relatively large, clearly convex ommatidia arranged in mostly easily recognisable vertical rows. Vertigial, supralabral, and labral setae: two, four, and 17, respectively. Antennae ca. 1.4 × as long as head in males and 1.1–1.15 × in females; antennomere 2 > 5 > 4 ≥ 3 > 6. Gnathochilarium with a conspicuously large promentum separating lamellae linguales > halfway, the latter with three or four setae in a longitudinal row; stipites in males parabasally each with a longitudinal row of several setae, non-setose in females. Collum smooth, with a single deep groove running along entire lateral margin.

Body rings somewhat vaulted. Prozonae with scattered, very short and shallow, longitudinal grooves. Metazonae rather deeply striated, n*_Schub_* = 6 or 7; metazonal setae relatively short, ca. 2/5 of (in more anterior rings), to equal to (in more posterior rings), metazonal length, arranged in very dense whorls. Ozopores placed right in pro-metazonal suture in more anterior rings, and tightly behind suture in more posterior ones; sutures not being sinuous in front of ozopores. Tarsus of mid-body legs 1.3 × as long as tibia and 3.8 × as long as apical claw.

***Telson*** (Fig. 41B): Epiproct very long (considerably surpassing the longest paraproctal setae in males, somewhat less pronounced in females), straight, distally bent ventrad, ending with a pointed hyaline tip; with a few setae. Hypoproct in males narrowly trapezoidal, tridentate, the teeth blunt, protruding behind rear contour of paraprocts; the same being narrowly rounded in females. Paraprocts rather sparsely setose, without distinct row of shorter setae along caudal margins.

**Figure 42.**
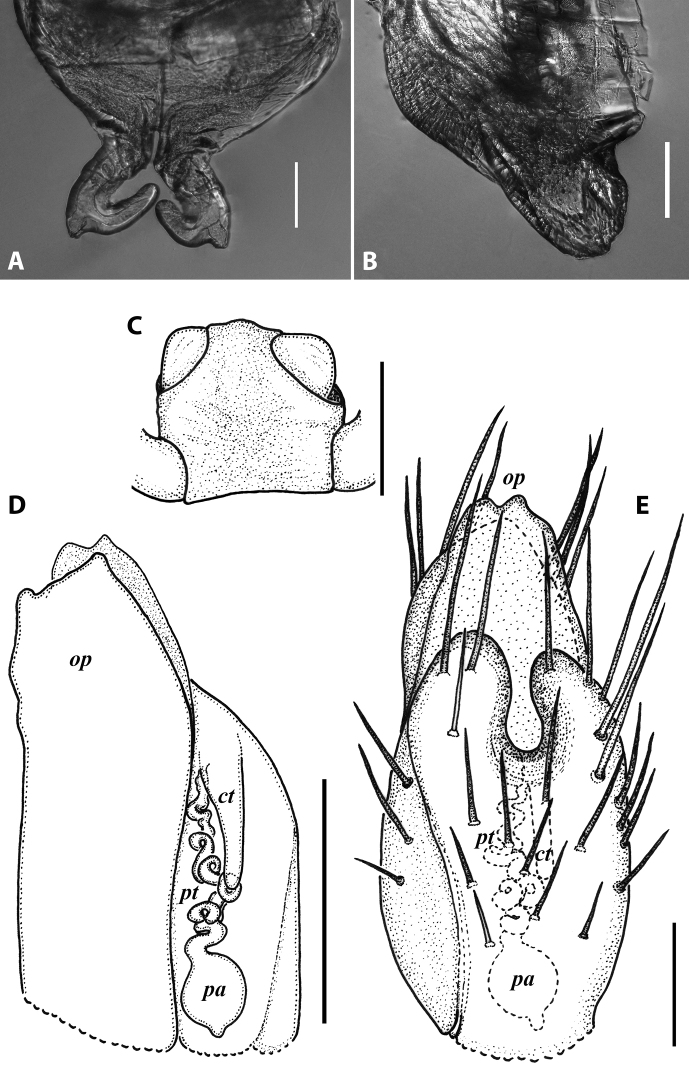
*Omobrachyiulusponticus* sp. nov., ♂ holotype (**A–C**) and ♀ paratype (**D, E**) **A** leg pair 1, caudal view **B** left flange of pleurotergum 7, ventro-lateral view **C** penis, caudal view **D** right vulva, lateral view (receptaculum seminis seen by transparency, but drawn with solid lines for emphasis; setae omitted) **E** same, caudal view. Scale bars: 0.2 mm (**D**), 0.1 mm (**A–C, E**). Abbreviations: ***ct*** central tube, ***op*** operculum, ***pa*** posterior ampulla, ***pt*** posterior tube.

***Male sexual characters*:** Mandibular stipites rather weakly expanded, forming a rounded ventro-anterior corner. Leg pair 1 (Fig. 42A) considerably converging (turned against one another) hooks with slender tibial outgrowths. Leg pair 2 and following pairs with crested adhesive pads, both tibial and postfemoral ones gradually reduced towards telson, but still visible after mid-body; femora of pairs 3–7 with an oval groove in the centre of a bulge. Pleurotergum 7 ventrally forming broadly rounded lobes (Fig. 42B) originating from the zone around pro-metazonal suture, protruding mesoventrad behind gonopods. Penis (42C) very small, subquadrate, without apical lobes and with very short, rounded, terminal lamellae directed distolaterad.

**Figure 43.**
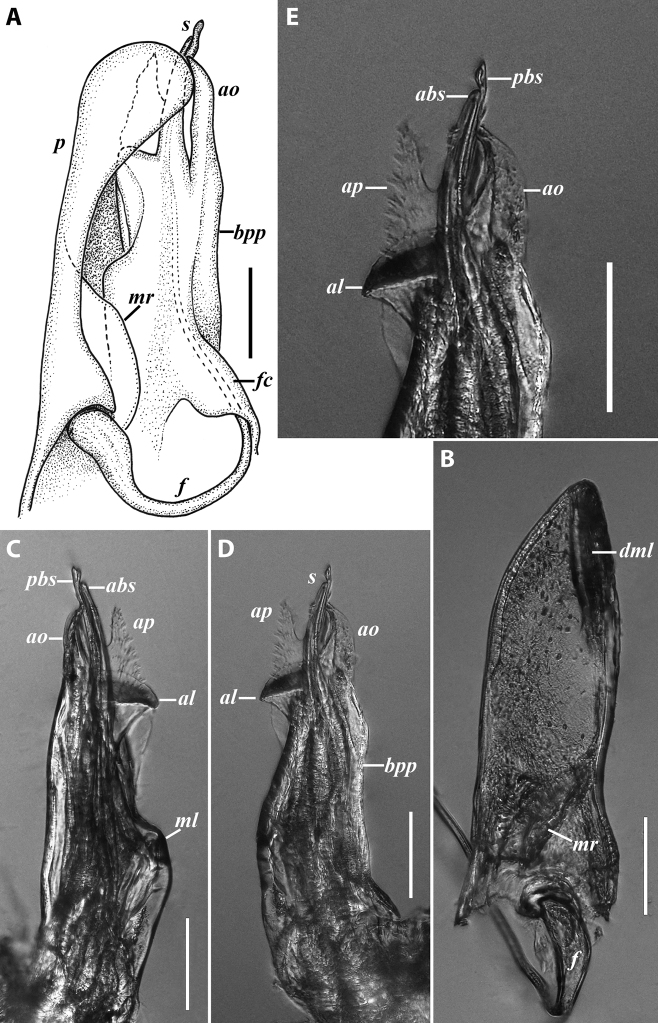
*Omobrachyiulusponticus* sp. nov., gonopods of ♂ holotype **A** left gonopods, mesal view **B** right promere, caudal view **C** right opisthomere, mesal view **D** same, caudo-lateral view **E** distal part of the same aspect. Scale bars: 0.1 mm. Abbreviations: ***abs*** anterior branch of solenomere, ***al*** apicolateral part of mesomeroidal lobe, ***ao*** apical outgrowth of basoposterior process, ***ap*** anterior process, ***bpp*** basoposterior process, ***dml*** distomesal lobe, ***f*** flagellum, ***fc*** flagellum channel, ***ml*** mesomeroidal lobe, ***mr*** median ridge, ***p*** promere, ***pbs*** posterior branch of solenomere, ***s*** solenomere.

***Gonopods*** (Fig. 43): Promere (Fig. 43B, p in Fig. 43A) slightly exceeded in height by solenomere, slender, with nearly symmetrical mesal and lateral margins joining in a narrow, blunt apex; median ridge short and strongly pronounced; a well-developed distomesal lobe. Opisthomere slender; basoposterior process weakly pronounced, ending in a tapering apical outgrowth turned slightly anteriad; anterior process shaped as a micro-serrate lamellar ridge with a freely protruding, tapering, apical part; mesomeroidal lobe with a moderately pronounced basal part, flattened distally and ending in an apicolateral part being very similar in shape to that in *O.kvavadzei* sp. nov.; solenomere deeply divided into two fine, slender, parallel branches: an anterior and a posterior one, the latter being slightly longer than the former; spiniform filaments along flagellum channel either absent or too small and slanting to be detected by light microscopy.

***Female sexual characters*:** Leg pairs 1 and 2 somewhat shorter, first also thicker, than following legs. Vulva (Fig. 42D, E) rather slender, similarly shaped to that of *O.kvavadzei* sp. nov., the main difference being the less strongly slanting apex of bursa, and the smaller, apically positioned opening in *O.ponticus* sp. nov.; both bursa and operculum moderately densely covered with long setae. Receptaculum seminis consisting of a short finger-shaped central tube, and a long, spirally twisted, posterior tube ending in an ovoid posterior ampulla with a minute pocket at bottom.

#### General distribution.

SWGC.

### Omobrachyiulus trochiloides

Taxon classificationAnimaliaJulidaJulidae

Vagalinski sp. nov.

A7E646D3-C74A-5A2D-BA1F-2628BA921E6B

http://zoobank.org/744C230E-01E6-4B1D-B76F-1EA4AEFF4570

Figs 44 , 45 , 46

#### Material examined.

***Holotype*:** ♂ (in head to ring 6, pleurotergum 7, and rest of body; distal part of left antenna broken off, gonopods and right flange of pleurotergum 7 on permanent slide) (ZMUM), Georgia, Borjomi Nature Reserve, Baniskhevi Valley, 800–900 m, *Picea*, *Fagus* and *Carpinus* forest, in litter, logs and under stones, 12 and 16.V.1983, SIG leg. ***Paratypes***: 1 ♂ (in head to ring 2, ring 3 to ring 6, and rest of body; gonopods prepared for SEM, penis, leg pair 2, leg 11 and mid-body leg on permanent slide) (ZMUM), 4 adult ♀♀ (one in head to ring 3 + rest of body, left vulva dissected) (ZMUM), 2 subadult ♀♀ (ZMUM), same collecting data as of the holotype; 1 ♂ (in head, collum to ring 6, pleurotergum 7 (in pieces), and ring 8 to rest of body; right antenna, leg pair 1, left flange of pleurotergum 7, and a mid-body leg dissected) (NMNHS), 1 adult and 2 subadult ♀♀ (unbroken) (NMNHS), Georgia, Chokhatauri District, near Bakhmaro, 4 km SSE Nabeglavi, 1950–2020 m, *Abiesnordmanniana*, 8.VI.1981, SIG and J. Martens leg.

#### Diagnosis.

A species of *Omobrachyiulus* most simillar to *O.sevangensis* comb. nov., *O.kvavadzei* sp. nov., and *O.ponticus* sp. nov. in gonopod structure, as well as in the shape of the male telson. Easily distinguishable from all these species by the massive, well-differentiated basoposterior process of the opisthomere, ending with a very fine and slender apical outgrowth, as well as by the solenomere being unipartite rather than bifurcated apically.

#### Name.

Derived from the resemblance of the apical outgrowth of the basoposterior process of the opisthomere to the beak of a hummingbird, family Trochilidae. Adjective.

#### Description.

***Measurements*:** male holotype in S VIII, 39+2+T, L = 17.5 mm, H = 1.1 mm; male paratypes in S VIII, 37+2+T, L = 12.8 mm, H = 1 mm, and 42+2+T, L = 18 mm, H = 1.2 mm; adult female paratypes in S IX–X, 40–46+0–1+T; L = 21–24 mm, H = 1.4–1.95 mm; subadult female paratypes in S VIII, 36–42+2+T.

**Figure 44.**
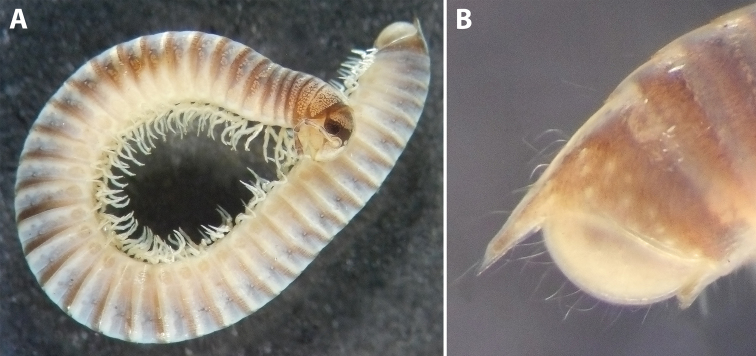
*Omobrachyiulustrochiloides* sp. nov. **A** habitus of ♀ paratype from Baniskhevi Valley, Georgia (ZMUM) **B** telson of ♂ holotype, lateral view. Not to scale.

***Colouration*** (after > 35 years in alcohol) (Fig. 44): Head brown with a broad, dark band between ommatidia; collum brown, with a much darker frontal quarter and a dark posterior margin; frontal parts of prozonae light grey to creamy, posterior parts dark grey with some brownish spots; frontal parts of metazonae dark brown, posteriorly light ochre-brown; whole ventral side of body light brownish beige, each prozona ventrolaterally with a large, darker, blurred spot; paraprocts brown-grey, lighter caudally; dorsum with a black axial line; legs yellowish, tibiae and tarsi in males brown.

***External structures*:** Eye patches in adults consisting of 28–35 ommatidia arranged in easily recognisable vertical rows. Vertigial, supralabral and labral setae: two, four, and 17–20, respectively. Antennae ca. 1.6 × as long as head in males and 1.2 × in females; antennomere 2 > 3 ~ 5 > 4 > 6. Gnathochilarium with a promentum separating lamellae linguales in their proximal 2/5, the latter with three or four setae in a longitudinal row; stipites with a group of short, stiff setae basomedially near the borders with the lamellae. Collum mostly smooth, with four or five very short and shallow striae at posterolateral corners.

Body rings weakly vaulted. Prozonae with scattered minute grooves. Metazonae rather shallowly striated, n*_Schub_* = 9 or 10; metazonal setae relatively short (ca. 1/2 of metazonal length) and erect. Ozopores placed right in pro-metazonal suture in first several rings, gradually taking a more posterior position to ~ 1/2 of their diameter in caudalmost rings; sutures not sinuous in front of ozopores. Tarsus of mid-body legs 1.4–1.5 × as long as tibia and 3.6–3.9 × as long as apical claw.

***Telson*** (Fig. 44B): Epiproct very long, straight to slightly bent ventrad, ending with a pointed hyaline tip turned ventrad, considerably surpassing longest paraproctal setae in both sexes. Hypoproct in males trapezoidal, tridentate, teeth well-developed, robust, protruding behind rear contour of paraprocts; the same being broadly rounded, completely concealed below paraprocts in females; ventral surface with five submarginal setae. Paraprocts moderately densely setose (ca. 20 long setae each side), without distinct rows of shorter setae along caudal margins.

**Figure 45.**
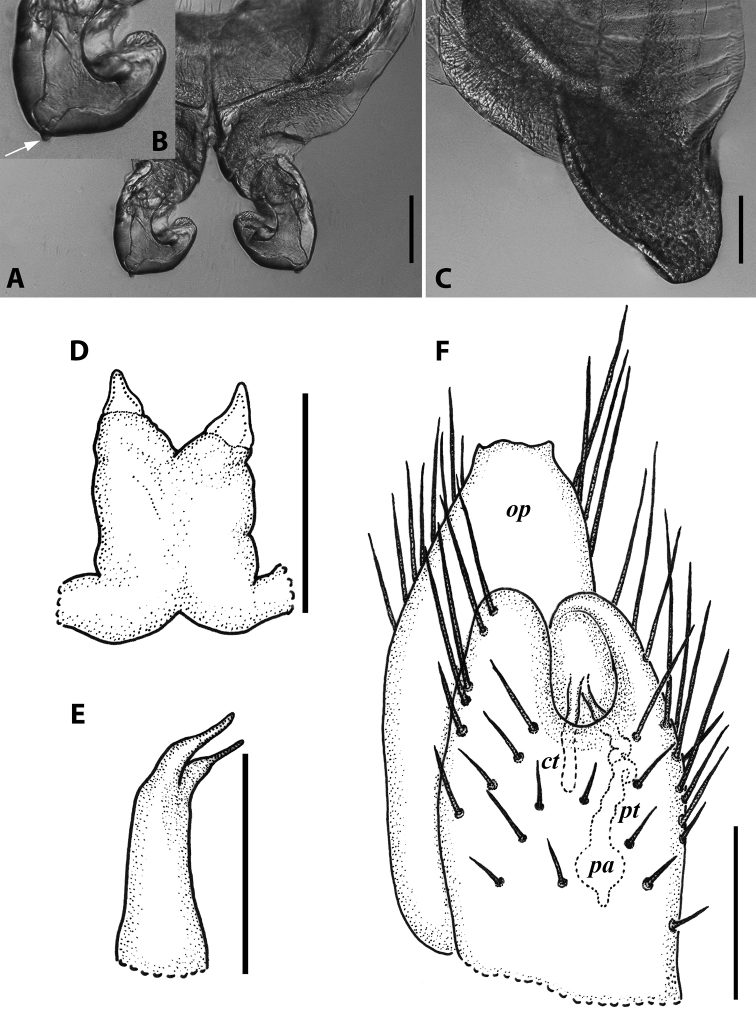
*Omobrachyiulustrochiloides* sp. nov., ♂ (**A, B, D, E**) and ♀ (**F**) paratypes from Baniskhevi Valley, Georgia (ZMUM), and ♂ paratype from near Nabeglavi, Georgia (NMNHS) (**C**) **A** leg pair 2, caudal view **B** distal part of left leg 1, caudal view **C** left flange of pleurotergum 7, ventro-lateral view **D** penis, caudal view **E** same, lateral view **F** left vulva, caudo-mesal view. Scale bars: 0.1 mm (**A, C**), 0.2 mm (**D–F**), not to scale (**B**). Abbreviations: ***ct*** central tube ***op*** operculum ***pa*** posterior ampulla ***pt*** posterior tube.

***Male sexual characters*:** Mandibular stipites moderately expanded, protruding ventro-anteriad, forming a broadly rounded anterior corner. Leg pair 1 (Fig. 45A, B) compact hooks turned somewhat against one another, bearing minute tarsal remnants (white arrow) in some specimens. Leg pair 2 and following pairs with crested adhesive pads ventrally on tibia and postfemur, these gradually reduced towards telson, completely disappearing in caudalmost legs; femora of legs 3–7 with an oval groove. Pleurotergum 7 ventrally forming large rounded lobes (Fig. 45C) originating mostly from metazona, protruding ventromesad behind gonopods. Penis (Fig. 45D, E) somewhat longer than broad, strongly compressed anterocaudally, with diverging apical lobes and well-developed, tapering, terminal lamellae.

**Figure 46.**
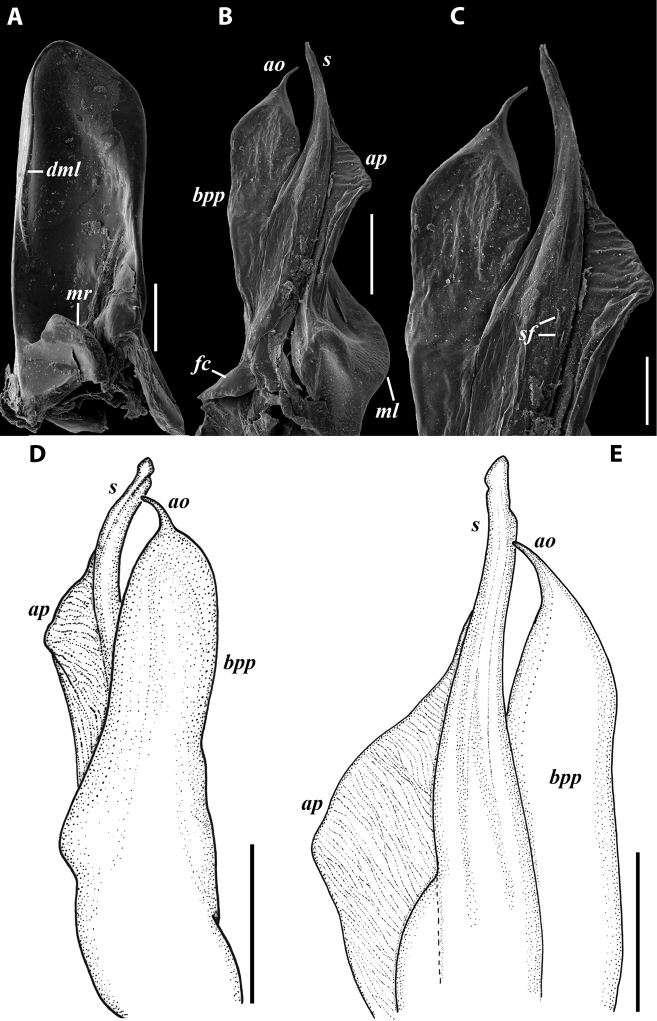
*Omobrachyiulustrochiloides* sp. nov., gonopods of ♂ paratypes from Baniskhevi Valley, Georgia (ZMUM) (**A–D**) and from near Nabeglavi, Georgia (NMNHS) (**E**) **A** left promere (flagellum broken off), caudal view **B** right opisthomere, mesal view **C** distal part of the same aspect **D** right opisthomere, lateral, somewhat caudal view **E** distal part of right opisthomere, lateral view. Scale bars: 0.2 mm (**D**), 0.1 mm (**A, B, E**), 0.05 mm (**C**). Abbreviations: ***ao*** apical outgrowth of basoposterior process, ***ap*** anterior process, ***bpp*** basoposterior process, ***dml*** distomesal lobe, ***f*** flagellum, ***fc*** flagellum channel, ***ml*** mesomeroidal lobe, ***mr*** median ridge, ***s*** solenomere.

***Gonopods*** (Fig. 46): In situ protruding from gonopodal sinus with their apical parts, laterally not concealed by protrusions of pleurotergum 7. Promere (Fig. 46A) as high as opisthomere, being conspicuously similar to that of *O.kvavadzei* sp. nov. and *O.ponticus* sp. nov. in overall shape, and particularly in having a short and strongly pronounced median ridge and a distomesal micro-squamous lobe; differing mostly by the broader apex. Opisthomere (Fig. 46B–E) rather slender; basoposterior process a well-developed and massive lobe, deeply divided from CBO, ending in a small, pointed, apical outgrowth bent anteriad; anterior process shaped as a furrowed lamellar ridge, completely fused to CBO; mesomeroidal lobe strongly pronounced, positioned at base of opisthomere, without any expanded parts; solenomere markedly slender, somewhat bent posteriad; only a few scattered spiniform filaments near flagellum channel.

***Female sexual characters*:** Leg pairs 1 and 2 somewhat thicker than following legs, leg pair 3 also slightly thicker and somewhat longer than neighbouring pairs. Vulva (Fig. 45F) very similar to that in *O.ponticus* sp. nov., including the external shape of the bursa, the structure of the receptaculum seminis, and the very high operculum; differs by a larger opening and a smaller central tube in relation to both posterior tube and ampulla.

#### General distribution.

LECA.

### Genus Svaniulus

Taxon classificationAnimaliaJulidaJulidae

Vagalinski gen. nov.

63312AF7-0C1A-58F9-B18B-247D975052CD

http://zoobank.org/7D6816A0-EC6B-40AE-8AB1-1F98B489BB90

#### Type species.

*Svaniulusryvkini* sp. nov., by present designation.

#### Included species.

*Svaniulusryvkini* sp. nov.

*Svaniuluswaltheri* sp. nov.

#### Diagnosis.

A genus of Brachyiulini differing from contribal genera by the following combination of characters: promeres positioned anteriorly and only slightly laterally in relation to opisthomeres; caudal surface of promere bearing a strongly developed mesal process; opisthomere possessing a broad and flattened basoposterior process and a faint mesomeroidal lobe, and lacking lateral and anterior processes; solenomere simple, more or less slender, ending with a sharply pointed tip; vulva subconical, bursa with completely fused valves (forming neither an opening nor a cleft), a supposed autapomorphy.

#### Name.

Honours the Svan people, the indigenous inhabitants of Svanetia, northwestern Georgia, whence the type species of the new genus originates. Gender: masculine.

#### General description.

- Medium-sized (L (males) = 23–28 mm) Brachyiulini.
- Ommatidia present.
- Ozopores set tightly behind pro-metazonal suture at least on more anterior body rings.
- Epiproct well-developed, relatively (not conspicuously) long.
- Hypoproct rounded trapezoidal to semi-circular, ventrally with two distal paramedian setae.
- Male mandibular stipites considerably expanded, forming a distinct ventro-anterior corner.
- Male pleurotergum 7 significantly bulging.
- Male walking legs ventrally with two well-developed adhesive pads, one each on postfemur and tibia.
- Penis short and stout, with very short apical lobes and small terminal lamellae.
- Gonopods:

- In situ: promeres considerably protruding outside gonopodal sinus, directed partly caudad, opisthomeres visible only with their apical parts.
- Promere slightly higher than opisthomere, elongate, bearing a long, stout, mesal process on caudal side; flagellum slightly shorter than height of promere.
- Opisthomere slender; basoposterior process well-developed, broad, flattened frontocaudally; anterior and apicoposterior processes absent; mesomeroidal lobe shaped as a weakly pronounced ridge; solenomere long and slender, distally bipartite, the frontal part ending in a fine, sharply pointed tip.

- Vulva:

- Subconical.
- Bursa without distinct postero-apical margin.
- Opening or median cleft absent – the two valves completely fused to one another.
- Operculum subequal in height to bursa.
- Receptaculum seminis: central tube short and narrow, ending in a distinct central ampulla; posterior tube long and narrow, ending in a subspherical posterior ampulla.

#### Remarks.

The new genus resembles *Omobrachyiulus* in the opisthomere having a mesomeroidal lobe (although weakly pronounced), a character not seen in other members of Brachyiulini. On the other hand, the long and freely protruding solenomere and the micro-papillary branches (albeit very small) apically on the opisthomeral basoposterior process suggest proximity to *Colchiobrachyiulus*, while the mesal process caudally on the promere might be homologous to the similarly positioned structure characteristic of the subgenus Rhamphidoiulus Attems, 1905 of the genus *Cyphobrachyiulus* Verhoeff, 1900. Anyhow, this unique combination of gonopodal characters, coupled with the supposedly autapomorphic condition of the vulva make the establishment of the new genus warranted.

### Svaniulus ryvkini

Taxon classificationAnimaliaJulidaJulidae

Vagalinski, gen. nov. sp. nov.

A198C84E-FCDF-5695-A525-9E142CDE39F6

http://zoobank.org/40C1EABC-B2E1-43A5-8F24-7A00391B88FD

Figs 47 , 48 , 49

#### Material examined

**(ZMUM): *Holotype***: ♂ (in head and 5 pieces, left antenna, penis, leg pairs 1 and 2, penis, gonopods, right flange of pleurotergum 7, mid-body leg, and hypoproct dissected), Georgia, Svanetia, mouth of Nenskra River, Lukhi, N of Khaishi, ca. 800 m a.s.l., leaf litter, 2.IX.1986, A. Ryvkin leg. ***Paratype***: 1 ♀ (in 2 pieces, left vulva dissected), 5 juv. (unbroken), same collecting data as for holotype.

#### Diagnosis.

Differs from its only known congener, *S.waltheri* gen. nov., sp. nov., mainly by being on average slightly smaller, by the relatively shorter male antennae, by the position and shape of the ventral protrusions of male body pleurotergum 7, and by the following details of gonopod structure: promere proportionally broader, abruptly narrowing only apically, with the mesal process significantly exceeding the apex, vs. the same being more slender, gradually narrowing distally, and with the mesal process just slightly exceeding the apex in *S.waltheri* gen. nov., sp. nov.; opisthomere possessing a distomesal process vs. this being absent from *S.waltheri* gen. nov., sp. nov., having a broad and flat apical margin of the basoposterior process, vs. the same forming a distinct apical corner in *S.waltheri* sp. nov., and with the solenomere gradually narrowing all the way to the top, vs. the same being somewhat enlarged apically in *S.waltheri* gen. nov., sp. nov.

#### Name.

Honours Aleksandr B. Ryvkin, a coleopterist and the collector of the specimens used for the description of this new species.

#### Description.

***Measurements*:** holotype in S IX, 42+1+T, L = 23 mm, H = 1.6 mm; paratype ♀ in S X, 46+1+T, L = 27 mm, H = 2.2 mm.

**Figure 47.**
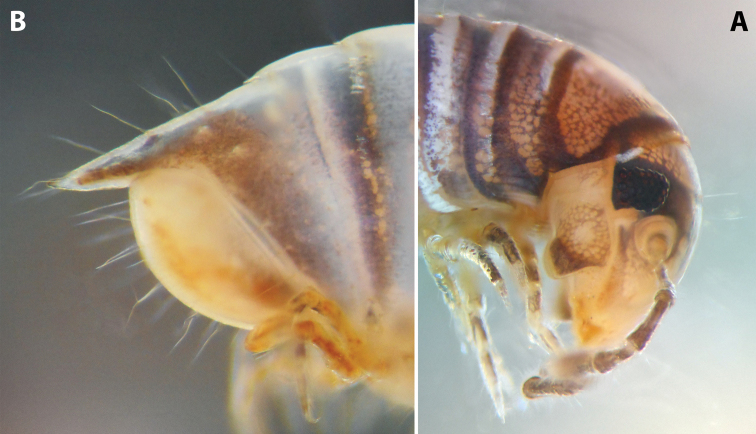
*Svaniulusryvkini* gen. nov., sp. nov., ♂ holotype **A** head and body rings 1–4 **B** telson, lateral views. Not to scale.

***Colouration*** (> 30 years in alcohol) (Fig. 47): Head and collum with the usual colour pattern, antennae dark brown; trunk dorsally mostly brown-grey, with a blackish axial line and with dark grey to blackish transverse stripes running along pro-metazonal suture on both pro- and metazonae, reaching down to ozopore level; contrasting lighter brown-beige below ozopore level; frontal sections of prozonae brown-beige, posteriorly with an irregular dark brown spot around ozopore; metazonae mostly lighter than prozonae, their posterior sections with a dark brown band encircling entire ring, these narrowing and lightening ventrally; hind margins ochre-brown; epiproct dorsally dark grey, laterally dark brown; hypoproct and proximal part of paraprocts mostly light brown; legs yellowish.

***External structures*:** Eye patches consisting of 27 or 28 and 37 or 38 markedly convex ommatidia, in the male and female, respectively; developmental rows easily countable. Vertigial, supralabral, and labral setae: two, four, and 20, respectively. Antennae ca. 1.35 × as long as head in the male, and 1.25 × in the female; antennomere 2 > 3 ≥ 5 ≥ 4 > 6. Gnathochilarium with a moderately sized promentum separating lamellae linguales by nearly half their length, each latter with four setae in a longitudinal row; male stipites basolaterally each with a faint oblong hump bearing several setae. Collum mostly smooth, with only several sparsely set, short and shallow grooves near posterolateral corners; frontolateral margin gently concave.

Body rings considerably vaulted. Prozonae with densely set, very short, shallow, parallel, longitudinal striae in their hind sections. Metazonae moderately deeply striated, n*_Schub_* = 9 or 10 in the male, and 10 or 11 in the female; setae erect to somewhat slanting, from ca. 2/5 (in mid-body and caudal rings) to equal to metazonal length (in anteriormost rings). Ozopores set tightly behind pro-metazonal suture in more anterior rings, and ca. half their diameter behind in more posterior ones; sutures gently to considerably sinuous in front of ozopores in most rings. Tarsus of mid-body legs equal to tibia, and slightly > 3 × as long as apical claw.

**Figure 48.**
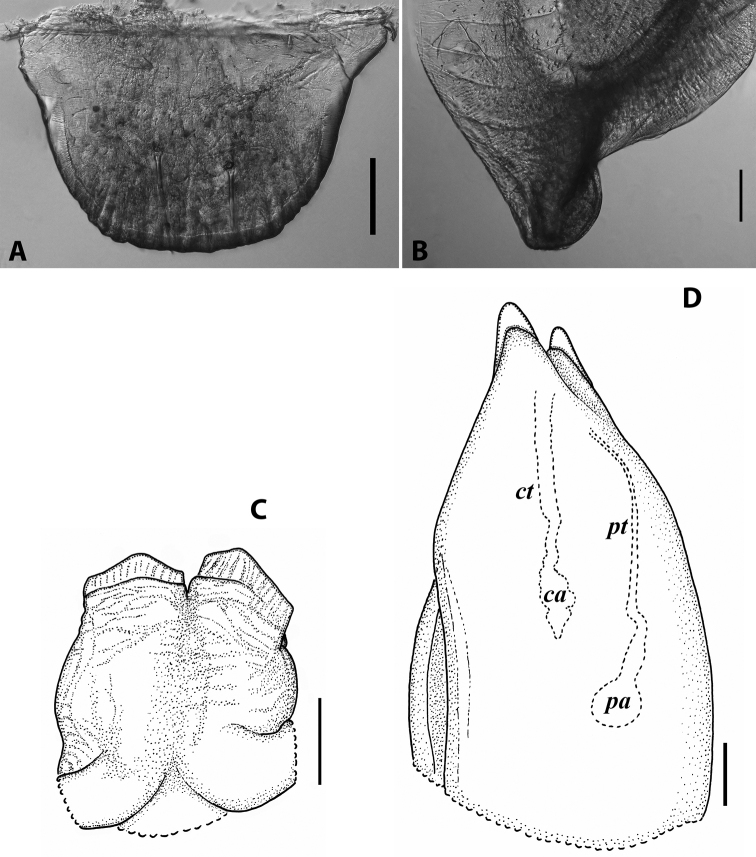
*Svaniulusryvkini* gen. nov., sp. nov., ♂ holotype (**A–C**) and ♀ paratype (**D**) **A** hypoproct, ventral view **B** right flange of pleurotergum 7, ventro-lateral view **C** penis, caudal view **D** left vulva, mesal view (setae omitted). Scale bars: 0.1 mm. Abbreviations: ***ca.*** central ampulla, ***ct*** central tube, ***pa*** posterior ampulla, ***pt*** posterior tube.

***Telson*** (Fig. 47B): Epiproct rather long (slightly exceeding the level of longest paraproctal setae in the male, just reaching it in the female) and straight, ending up with a well-developed, blunt, unciform, hyaline tip bent dorsad; with several long setae on dorsal side. Hypoproct in the male (Fig. 48A) rounded trapezoidal, with a thickened, faintly undulate, caudal margin slightly protruding behind caudal contour of paraprocts, this being broadly rounded, thinner, and tightly adhering under paraprocts in the female; with a pair of long distal paramedian setae. Paraprocts rather sparsely covered with long setae, without rows of shorter setae at caudal margins.

***Male sexual characters*:** Mandibular stipites (Fig. 47A) considerably expanded, protruding ventro-anteriad, forming a subrectangular, rounded, ventro-anterior corner. Leg pair 1 compact parallel hooks. Leg pair 2 slightly shorter and thicker than following legs, both tibial and postfemoral adhesive pads well-developed, crested in anterior part of body, gradually reduced in the last third, completely absent from caudalmost legs; femora without modifications. Pleurotergum 7 ventrally forming blunt and spade-shaped lobes (Fig. 48B) originating entirely from metazona, oriented with their broad sides parallel to the body axis, protruding mostly mesad, touching one another behind gonopods. Penis (Fig. 48C) very small, deeply hidden above coxae 2, barrel-shaped, slightly longer than broad, strongly flattened dorso-ventrally, with very short apical lobes ending in short and blunt terminal lamellae directed distad.

**Figure 49.**
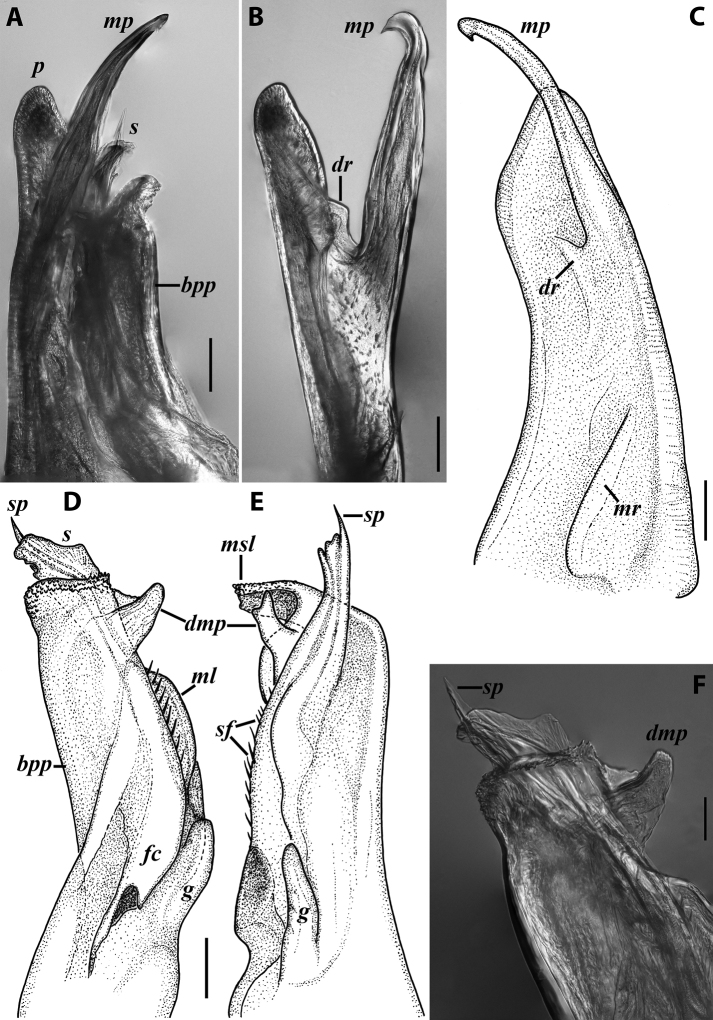
*Svaniulusryvkini* gen. nov., sp. nov., gonopods of ♂ holotype **A** left gonopods, mesal view **B** distal part of right promere, lateral view **C** right promere, caudal view **D** right opisthomere, meso-caudal view **E** same, oral view **F** distal part of the same, meso-caudal view. Scale bars: 0.1 mm (**A–E**), 0.05 mm (**F**). Abbreviations: ***bpp*** basoposterior process, ***dmp*** distomesal process, ***dr*** distal ridge, ***f*** flagellum, ***fc*** flagellum channel, ***g*** (supposed) gonocoxal gland, ***ml*** mesomeroidal lobe, ***mp*** mesal process, ***mr*** median ridge, ***msl*** mesal lobe of basoposterior process, ***p*** promere, ***pt*** posterior tube, ***s*** solenomere, ***sf*** spiniform filaments, ***sp*** apical process of solenomere.

***Gonopods*** (Fig. 49): Promere (Fig. 49B, C and p in Fig. 49A) relatively slender, somewhat higher than opisthomere, leaf-shaped, with a gently convex mesal margin, and a markedly sigmoid lateral one, both joining in a narrowly rounded apex; caudal surface with a massive and long mesal process originating at mid-height, ending with a short unciform apex turned baso-anteriad, considerably exceeding the apex of main promeral body; a short, well-pronounced, median ridge, a broad and weakly defined median groove, and a short and well-pronounced distal ridge just lateral to base of mesal process; flagellum just slightly shorter than height of promere. Opisthomere (Fig. 49A, D–F) slender; basoposterior process present as a broad, anterocaudally flattened lobe ending with a flat apical margin bent caudad and bearing short and micro-papillary branches, mesally expanding into a short lobe with a coarse margin; mesomeroidal lobe a weakly pronounced ridge; a thumb-like distomesal process originating from border between basoposterior process and solenomere, directed distomesad; anteromesally at base a well-developed, elongate lobe (presumably gonocoxal gland); solenomere unipartite, long, flattened on sides, gradually narrowing distad, gently sigmoid, caudo-apically with a very fine and sharply pointed process; a dense row of small, erect, spiniform filaments at flagellum channel’s mid-height.

***Female sexual characters*:** Leg pairs 1 and 2 somewhat shorter, 1^st^ also slightly thicker, than following legs. Vulva (Fig. 48D) slightly asymmetrical (lateral valve of bursa more strongly slanting towards apex compared to mesal one), rather elongate, subconical, strongly compressed on sides; bursa with a distinct, obtuse, postero-apical margin; an opening absent, with the two valves completely fused all the way to bursal apex; operculum slightly shorter than bursa, both structures ending up with ear-like hyaline protrusions, bursal ones considerably larger; setation dense throughout. Receptaculum seminis composed of a short and very narrow central tube abruptly widening into an oblong central ampulla, forming several constrictions followed by a digitiform reservoir at bottom; and a long and thin, slightly folded, posterior tube, this somewhat widening before ending in a subspherical posterior ampulla.

#### General distribution.

SWGC.

#### Remarks.

In the absence of a bursal opening and considering the position of the central tube of the vulval receptaculum, which is displaced strongly anteriad, at the border with the operculum, the only way for the male opisthomere to reach the receptaculum would be through widening the gap between the bursa and operculum, with the slender, gradually attenuating solenomere ending with a short and sharply pointed process that seems to serve as a perfect tool for that purpose. This is an obvious and rare example of co-evolution of gonopods and vulvae within Julidae, the family being generally characterised by highly specialised posterior gonopods with species-specific apical parts, in contrast to the more simply and uniformly built vulvae which are often barely distinguishable even between different genera of the same tribe.

### Svaniulus waltheri

Taxon classificationAnimaliaJulidaJulidae

Vagalinski, gen. nov., sp. nov .

C891E4EE-1E0E-5AD4-BDDA-2959F415A530

http://zoobank.org/15B28D68-1A07-49AA-9F81-717705C8F0AD

Figs 50 , 51 , 52

#### Material examined

**(SMNG): *Holotype***: ♂ (in head to pleurotergum 7 and rest of body, gonopods intact), Georgia: Samegrelo-Zemo Svaneti, Lebarde, Tekhuri Valley, 30 rkm from Taleri towards Lebarde [at the influx of Lebarde and Tekhuri rivers], 42.7258°N, 42.4703°E, 1260 m a.s.l., 10.X.2011, F. Walther leg. ***Paratypes***: 1 ♂ (in 3 pieces, leg 7, mid-body and end-body leg, hypoproct, left flange of pleurotergum 7, and penis dissected, gonopods prepared for SEM), 1 ♀ (in head to body ring 3 and rest of body, vulvae dissected, right one prepared for SEM), same collecting data as of the holotype.

#### Diagnosis.

Differs from its only known congener, *S.ryvkini* gen. nov., sp. nov., mostly by being on average slightly larger, by the relatively more slender male antennae, by the position and shape of the ventral protrusions of male pleurotergum 7, and by the following details of gonopod structure: promere more slender, distally significantly narrowing, with the mesal process barely exceeding the apex, vs. the same being somewhat stouter, only apically abruptly narrowing, and with the mesal process significantly exceeding the apex in *S.ryvkini* gen. nov., sp. nov.; opisthomere lacking a distomesal process, vs. this being present in *S.ryvkini* gen. nov., sp. nov., having the basoposterior process with a distinct apical corner, vs. the same ending in a broad and flat margin in *S.ryvkini* gen. nov. sp. nov., and with the solenomere being somewhat enlarged apically, vs. the same narrowing all the way to the top in *S.ryvkini* gen. nov., sp. nov.

#### Name.

Honours Frank Walther, a malacologist and an active collector of various invertebrate groups, including the material used for the present description.

#### Description.

***Measurements*:** holotype in S X, 43+1+T, L = 26 mm, H = 1.8 mm; paratype ♂ in S IX, 43+1+T, L = 27.5 mm, H = 1.75 mm; paratype ♀ in S IX, 44+1+T, L = 37.5 mm, H = 2.65 mm.

**Figure 50.**
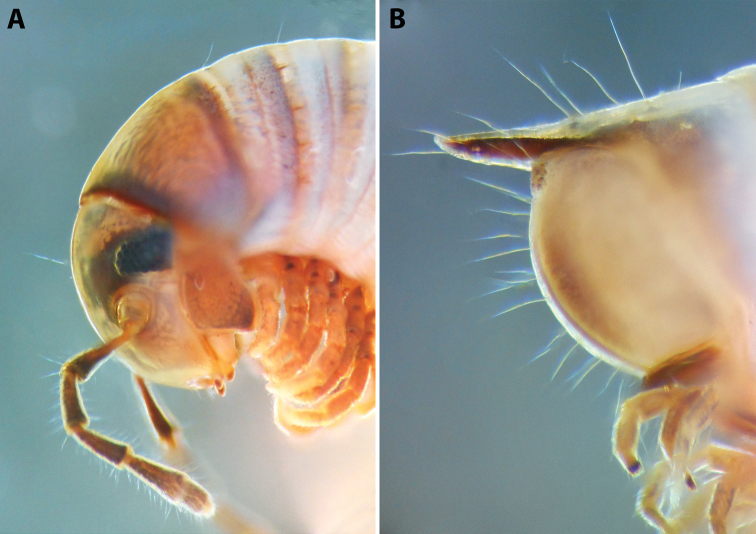
*Svaniuluswaltheri* gen. nov., sp. nov., ♂ paratype **A** head and body rings 1–3 **B** telson, lateral views. Not to scale.

***Colouration*** (apparently somewhat faded) (Fig. 50): Similar to *S.ryvkini* gen. nov., sp. nov., but generally lighter, predominantly grey rather than brown, without significant difference between dorsal and ventral sides, and without transverse dark stripes along pro-metazonal sutures; prozonae dark grey, with numerous, densely set, light brown-beige spots arranged transversely above and below ozopore level; metazonae mostly grey, with numerous, minute, dark brown/blackish dots throughout, dorsally with a very narrow, transverse, blurred, dark brown band in posterior section; hind margins ochre-brown.

***External structures*:** Eye patches in adults vertically rather elongate, due to partial reduction of the higher developmental (vertical) rows, consisting of 21–35 markedly convex ommatidia. Vertigial, supralabral, and labral setae: two, four, and 16–18, respectively. Antennae (Fig. 50A) 1.6–1.8 × as long as head in the males, and 1.25 × in the female; antennomere 2 > 5 ≥ 3 > 4 > 6. Gnathochilarium as in *S.ryvkini* gen. nov., sp. nov., but lamellae linguales with three rather than four setae in a longitudinal row, and male stipites without setose hump.

**Figure 51.**
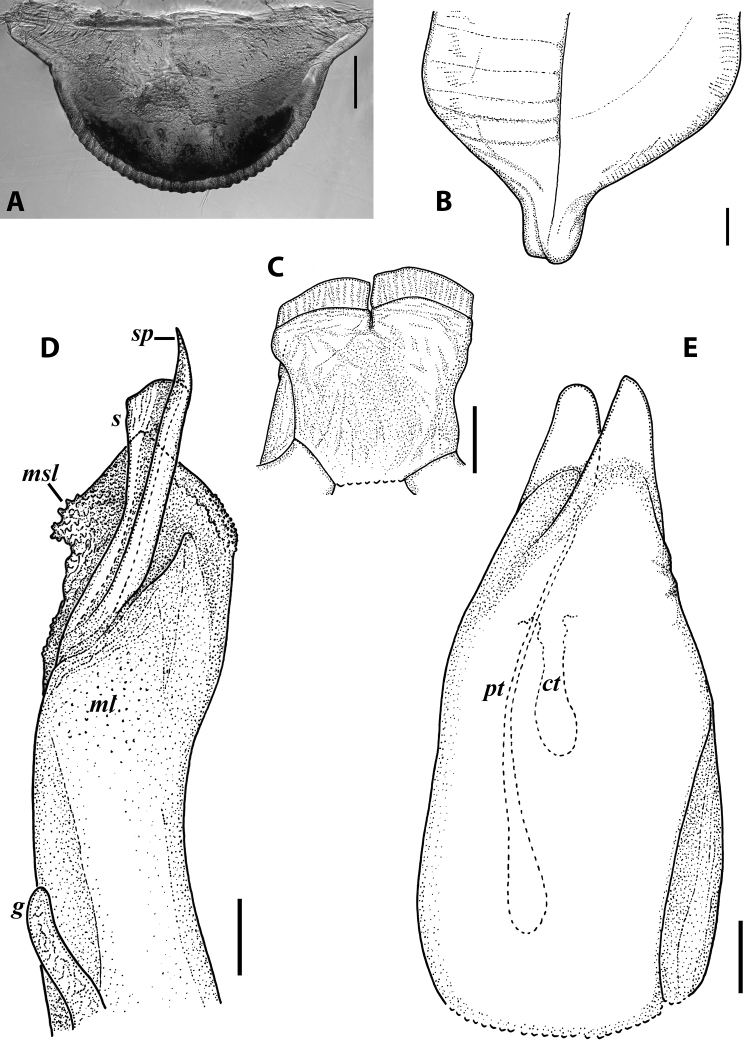
*Svaniuluswaltheri* gen. nov., sp. nov., ♂ (**A–D**) and ♀ (**E**) paratypes **A** hypoproct, ventral view **B** right flange of pleurotergum 7, ventro-lateral view **C** penis, caudal view **D** right opisthomere, oral view **E** left vulva, latero-caudal view. Scale bars: 0.1 mm. Abbreviations: ***ct*** central tube, ***g*** (supposed) gonocoxal gland, ***ml*** mesomeroidal lobe, ***msl*** mesal lobe of basoposterior process, ***pt*** posterior tube, ***s*** solenomere, ***sp*** apical process of solenomere.

Body rings slightly to moderately vaulted. Male hypoproct (Fig. 51A) very similar to that in *S.ryvkini* gen. nov., sp. nov., but almost perfectly semi-circular and with a densely and bluntly serrate margin, rather than apically somewhat flattened and with only several faint undulations. Tarsus of mid-body legs subequal to, to slightly longer than, tibia, and ca. 3 × as long as apical claw.

***Male sexual characters*:** Walking legs with adhesive pads still present in caudalmost pairs. Pleurotergum 7 ventrally forming rather narrow and oar-like lobes (Fig. 51B) with a slightly concave apical margin, originating from border zone between pro- and metazona, protruding mostly ventrad behind gonopods. Penis (Fig. 51C) similar to that in *S.ryvkini* gen. nov., sp. nov., but trapezoidal (widening distally) rather than barrel-shaped.

All other external somatic characters as in *S.ryvkini* gen. nov., sp. nov.

**Figure 52.**
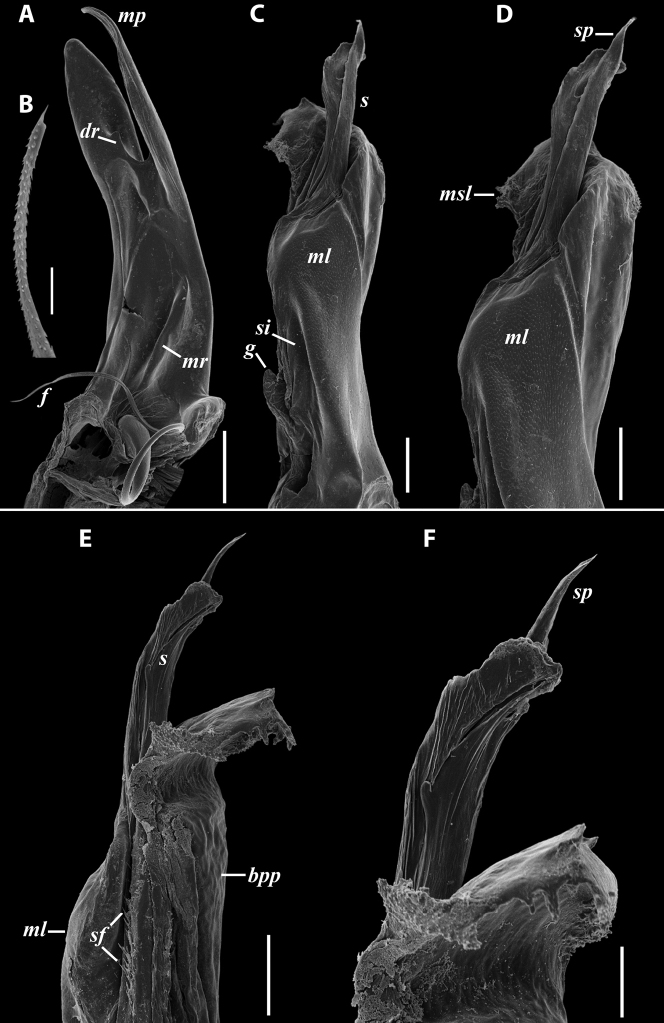
*Svaniuluswaltheri* gen. nov., sp. nov., gonopods of ♂ paratype **A** right promere, caudal view **B** apical part of flagellum **C** right opisthomere, oral view **D** distal part of the same, latero-oral view **E** distal part of left opisthomere, caudo-mesal view **F** apical part of the same, meso-caudal view. Scale bars: 0.1 mm (**A, C–E**), 0.05 mm (**F**), 0.01 mm (**B**). Abbreviations: ***bpp*** basoposterior process, ***dr*** distal ridge, ***f*** flagellum, ***g*** (supposed) gonocoxal gland, ***ml*** mesomeroidal lobe, ***mp*** mesal process, ***mr*** median ridge, ***msl*** mesal lobe of basoposterior process, ***s*** solenomere, ***sf*** spiniform filaments, ***sp*** apical process of solenomere.

***Gonopods*** (Figs 51D, 52): Promere (Fig. 52A) similar to that in *S.ryvkini* gen. nov., sp. nov., but more slender, distally mostly symmetrical rather than with a markedly convex lateral margin; mesal process barely, rather than considerably, surpassing the apex of promere, and ending with a finer, less strongly bent tip; median and distal ridges less strongly pronounced compared to the type species. Opisthomere (Figs 51D, 52C–F): lacking a distomesal process; basoposterior process narrower in comparison to *S.ryvkini* gen. nov., sp. nov., with a distinct apical corner rather than with a flat margin, and with a much smaller mesal lobe; mesal side with a slender lobe (presumably gonocoxal gland) and a rather deep and narrow anteromesal sinus; solenomere somewhat enlarged distally rather than narrowing; otherwise of a very similar shape to that of the type species.

***Female sexual characters*:** First two leg pairs of same condition as in *S.ryvkini* gen. nov., sp. nov. Vulva (Fig. 51E) externally also very similar to the type species, mainly differing in being more elongated and with the operculum being equal to, rather than slightly shorter than, bursa. Receptaculum seminis with a somewhat wider and shorter central tube, not forming a distinct ampulla at bottom; posterior tube gradually widening towards bottom, rather than constricting to form an ampulla.

#### Remark.

Despite the distance between the type localities of *S.waltheri* gen. nov., sp. nov. and *S.ryvkini* gen. nov., sp. nov. being only ca. 35 air-km, the distribution areas of the two species are probably divided by the Egrisi Mountain Range. With summit parts above 3000 m a.s.l., it could have split the population of the common ancestor of these two closely related species during colder periods in the geological past.

#### General distribution.

SWGC.

### Key to the species of Brachyiulini in the Caucasus region based on male gonopodal and external somatic characters

**Table:** 

1	Promere nearly half as high as opisthomere	**2**
–	Promere subequal to, or somewhat higher than, opisthomere	**4**
2	Opisthomere with a prominent anterior process and a broad and flattened lateral process; without apicoposterior process	(**genus *Brachyiulus*) 3**
–	Opisthomere with neither an anterior nor a lateral process, but with a well-developed apicoposterior process protruding perpendicular to CBO (Fig. 6B)	**Cyphobrachyiulus (Grusiniulus) redikorzevi (Lohmander, 1936)**
3	Lateral process of opisthomere finely and densely striated, anterior process strongly bent caudad	***Brachyiuluslusitanus* Verhoeff, 1898**
–	Lateral process of opisthomere smooth, anterior process only slightly bent caudad	***Brachyiulusjawlowskii* Lohmander, 1928**
4	Hypoproct triangular	**5**
–	Hypoproct trapezoidal, semi-elliptic or semi-circular	**6**
5	Opisthomere with a large lateral process nearly level to promere, and a rounded lobe-like basoposterior process; lacking an apicoposterior process (Fig. 1A, B, D). N Caucasus	**Byzantorhopalum(s. str.)rossicum (Timotheew, 1897)**
–	Opisthomere lacking both lateral and basoposterior processes, but possessing a thumb-like, micro-dentate, apicoposterior process	**Cyphobrachyiulus (Diaxylus) litoreus (Lignau, 1903)**
6	Promere with a distinct, smaller or larger, (apico/disto)mesal process	**7**
–	Promere without prominent processes, only with small to moderate lobes in some species	**11**
7	Opisthomere with a weakly pronounced basoposterior process ending with a visor-like apical outgrowth protruding nearly perpendicularly to CBO; solenomere complex, multibranched (Fig. 7B–E); mesal process of promere originating from apex (Fig. 7A). Svanetia	***Iraniulustricornis* sp. nov.**
–	Opisthomere with a more or less well-developed basoposterior process in the shape of a broad lobe with a freely protruding apical part; solenomere simple, slender, apically sharply pointed; mesal process of promere originating subapically or medially	**8**
8	Mesal process stout and long, originating at promere mid-height; promeres positioned mostly anteriorly and only slightly laterally in relation to promeres. Svanetia and Megrelia	**(genus *Svaniulus*) 9**
–	Mesal process fine and rather short, originating from a distal or apical part of promere; promeres positioned completely laterally in relation to opisthomeres. W Greater Caucasus and N Colchis	**(genus *Colchiobrachyiulus*) 10**
9	Male antennae 1.3–1.4 × as long as head; male pleurotergum 7 ventrally with blunt, spade-like lobes originating entirely from metazona (Fig. 48B); opisthomere with a thumb-like distomesal process (Fig. 49D–F)	***Svaniulusryvkini* gen. nov., sp. nov.**
–	Male antennae 1.6–1.8 × as long as head; male pleurotergum 7 ventrally with narrow oar-like lobes with slightly concave apical margins, originating from border zone between pro- and metazona (Fig. 51B); opisthomere lacking a distomesal process (Figs 51D, 52C–F)	***Svaniuluswaltheri* gen. nov., sp. nov.**
10	Body length usually > 30 mm, vertical diameter > 2 mm; promere with its distomesal process significantly outreaching the apex (Fig. 3A); solenomere bipartite distally (Fig. 3B, C). AR Abkhazia	***Colchiobrachyiulusdioscoriadis* (Lignau, 1915)**
–	Body usually < 20 mm in length, vertical diameter < 1.3 mm; promere with its distomesal process being subequal to apex (Fig. 5C, D); solenomere unipartite all along. Karachay-Cherkess Republic	***Colchiobrachyiulusmontanus* sp. nov.**
11	Male hypoproct trapezoidal, with apical margin < half the width at base, tridentate; opisthomere with a distinct basoposterior process, clearly separated from CBO in its distal half or so, a mesomeroidal lobe absent	**(genus *Megaphyllum* , subgenus Megaphyllum) 12**
–	Male hypoproct either rounded or trapezoidal, with apical margin just slightly narrower than base, often forming three or more denticles. Basoposterior process mostly fused to CBO, with only a freely protruding apical outgrowth; anterior surface of opisthomere with a more or less well-developed mesomeroidal lobe	**(genus *Omobrachyiulus*) 13**
12	Body uniformly dark with an orange to dark red mid-dorsal line; promere with mostly parallel side margins, ending with a flat apex	**Megaphyllum(s. str.)hercules (Verhoeff, 1901)**
–	Body with a light yellow to ochre dorsum divided by a black axial line; promere significantly tapering all the way to a narrowly rounded apex	**Megaphyllum(s. str.)spathulatum (Lohmander, 1936)**
13	Promere considerably higher than opisthomere (cf. Figs 20A, 23A)	**14**
–	Promere subequal in height to opisthomere (cf. Figs 16A, 43A)	**16**
14	Male hypoproct very broadly trapezoidal, margin with three large denticles (Fig. 19A); apical outgrowth of opisthomeral basoposterior process broad, collar-shaped, its margin dentate (Figs 19E, F, 20C–E)	***Omobrachyiulusarmatus* sp. nov.**
–	Hypoproct rounded, edentate; apical outgrowth different in shape	**15**
15	Basoposterior process a well-pronounced lobe ending in a tripartite apical outgrowth; an anterior process absent or vestigial (Fig. 17A, C). Colchis, Svanetia?	***Omobrachyiulushortensis* (Golovatch, 1981)**
–	Basoposterior process a rather weakly pronounced lobe ending in a leaf-like (from mesal and caudal views) apical outgrowth serrate at margin (Figs 22C, 23C); anterior process large, distally tapering (Fig. 23E). W Lesser Caucasus	***Omobrachyiuluspristis* sp. nov.**
16	Apical outgrowth of basoposterior process simple, mostly smooth, and more or less tapering distad	**17**
–	Apical outgrowth more elaborate, multipartite or bearing various small lobes or denticles	**20**
17	Opisthomere with a very prominent mesomeroidal lobe and with solenomere bent markedly caudad	***Omobrachyiulusgeniculatus* (Lohmander, 1928)**
–	Mesomeroidal lobe either well-developed but moderately pronounced, or positioned at the very base of opisthomere; solenomere directed mostly distad	**18**
18	Apical outgrowth of basoposterior process more or less slender, strongly tapering, bent partly or completely frontad (Figs 39G, 40C, D, 43E, 46C, E); anterior process at least partly developed as a vertical and furrowed lamella (Figs 39G, 43E, 46C, E). Epiproct apically slightly to considerably turned ventrad. Small species (length usually < 17 mm, height < 1.3 mm)	**the *Omobrachyiulussevangensis* group (Table 2)**
–	Apical outgrowth more robust, directed mostly distad; anterior process a short spine or a slender rod. Epiproct with the tip slightly to considerably turned dorsad. Medium-sized species (length usually > 20 mm, height > 1.5 mm)	**19**
19	Apical outgrowth narrow, ending bluntly (Fig. 12C, D); anterior process vestigial, in the shape of a small spine at solenomere mid-height (Fig. 12D). Epiproct short	***Omobrachyiuluscaucasicus* (Karsch, 1881)**
–	Apical outgrowth broad, roughly diamond-shaped; anterior process long, distally drawn out into a fine rod. Epiproct long	***Omobrachyiuluscurvocaudatus* (Lignau, 1903)**
20	Hypoproct rounded, edentate, rarely with three weakly pronounced and rounded teeth or undulations, with two distal paramedian setae (cf. Figs 26A, 29A, 33A)	**21**
–	Hypoproct trapezoidal, margin clearly dentate, usually with a row of more than two submarginal setae (cf. Fig. 15A)	**24**
21	Opisthomere with a moderately to strongly pronounced mesomeroidal lobe; anterior process smaller or larger, but always clearly detached from solenomere; solenomere rather thick. Republic of Adygea, Stavropol Territory	**22**
–	Mesomeroidal lobe rather weakly pronounced; an anterior process either absent or present as an indistinct ridge, mostly fused to solenomere; solenomere slender. AR Abkhazia, Krasnodar Province, Republic of Adygea	**the *Omobrachyiulusimplicitus* group (Table 1)**
22	Apical outgrowth of basoposterior process massive, bent strongly basofrontad, covering the basomesal side of solenomere like a hood (Figs 33E, 34E); mesomeroidal lobe forming a pointed pyramidal structure distally (Figs 33E, 34C, E). Metazonal setae present	***Omobrachyiulusfaxifer* sp. nov.**
–	Apical outgrowth flattened, shield-like, oriented distad; mesomeroidal lobe forming an apicolateral and an apicomesal part distally. Metazonal setae absent	**23**
23	Anterior process well-developed, linguiform, visible from most angles; both apicomesal and apicolateral parts of mesomeroidal lobe rather weakly pronounced; solenomere apically only moderately enlarged, bearing a soft sigmoid process directed basad (Fig. 31). Republic of Adygea	***Omobrachyiulusroseni* (Verhoeff, 1921)**
–	Anterior process vestigial, mostly hidden between base of solenomere and distal part of mesomeroidal lobe (Fig. 37D); apicolateral part of mesomeroidal lobe strongly pronounced (Fig. 37B–D); solenomere with a strongly enlarged, flower-like apex (Fig. 37E). The vicinity of Stavropol City	***Omobrachyiuluszuevi* sp. nov.**
24	An anterior process absent. AR Ajara	***Omobrachyiulusadsharicus* (Lohmander, 1936)**
–	Anterior process well-developed, slender, directed distad	**25**
25	Apical outgrowth of basoposterior process pillow-like, its margin with several small humps and undulations. Epiproct very long and broad	***Omobrachyiulusmacrourus* (Lohmander, 1928)**
–	Apical outgrowth broad, leaf-shaped, deeply divided into two or three lobes. Epiproct of moderate size	**26**
26	Apical outgrowth mostly symmetrical, with a central part and two side alate parts; anterior process equal to, or slightly higher than, solenomere, the latter bent abruptly caudad (Fig. 13)	***Omobrachyiulusdivaricatus* (Lohmander, 1936)**
–	Apical outgrowth strongly asymmetrical, forming a dentate distal part and a thumb-like lateral part; anterior process significantly outreached by solenomere, the latter nearly straight, directed almost completely distad (Fig. 16A, C–E)	***Omobrachyiulusunugulis* sp. nov.**

## Discussion

### Distribution patterns of the Caucasian Brachyiulini

The present list of 32 species of Brachyiulini (more than a quarter of all described species of the tribe (see Vagalinski and Lazányi 2018)) is another confirmation of the exceptionally rich Caucasian diplopod fauna, as already mentioned in Introduction. The majority of these are narrow local endemics (22, or 69%), while five species, or 16%, are regional endemics, and only another 5% occur also outside the Caucasus. Of the 14 currently recognised brachyiulinine genera, eight are present in the Caucasus, which is unequivocal evidence for the key role of the region for the diversification of this particular group of Julidae. Two genera, *Colchiobrachyiulus* Lohmander and *Svaniulus* gen. nov., with two species each, are Caucasian endemics, while *Iraniulus* Attems, with one species known from Svanetia and another from Hyrcania, can be considered as a Caucasian subendemic.

**Map 1.**
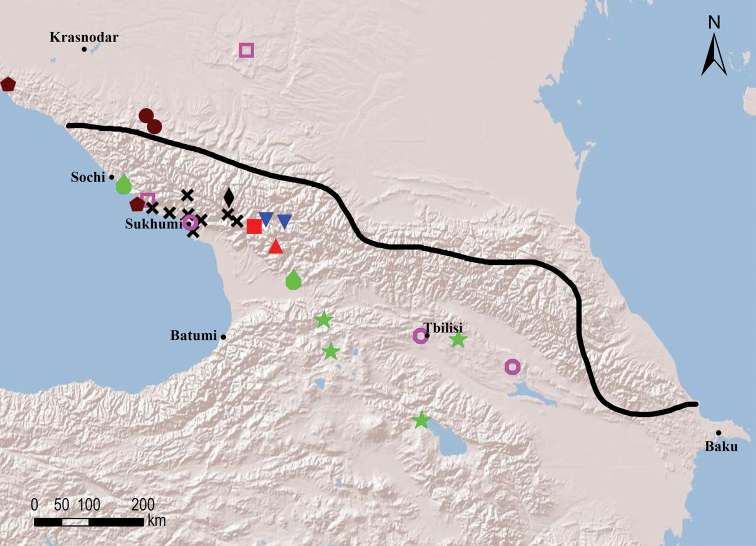
Distribution of the genera *Brachyiulus* (purple), *Cyphobrachyiulus* (green), *Colchiobrachyiulus* (black), *Iraniulus* (blue) *Megaphyllum* (brown), and *Svaniulus* (red) in the Caucasus: *Brachyiulusjawlowskii* (open square), *B.lusitanus* (ring), Cyphobrachyiulus (Diaxylus) litoreus (drop), C. (Grusiniulus) redikorzevi comb. nov. (star), *Colchiobrachyiulusdioscoriadis* comb. nov. (cross), *C.montanus* sp. nov. (diamond), *I.tricornis* sp. nov. (triangle pointing down), *Megaphyllumhercules* (pentagon), *M.spathulatum* (filled circle), *S.ryvkini* gen. nov., sp. nov. (filled square), and *S.waltheri* gen. nov., sp. nov. (triangle pointing up); solid line representing the approximate south border of the distribution range of *Byzantorhopalumrossicumrossicum*.

**Map 2.**
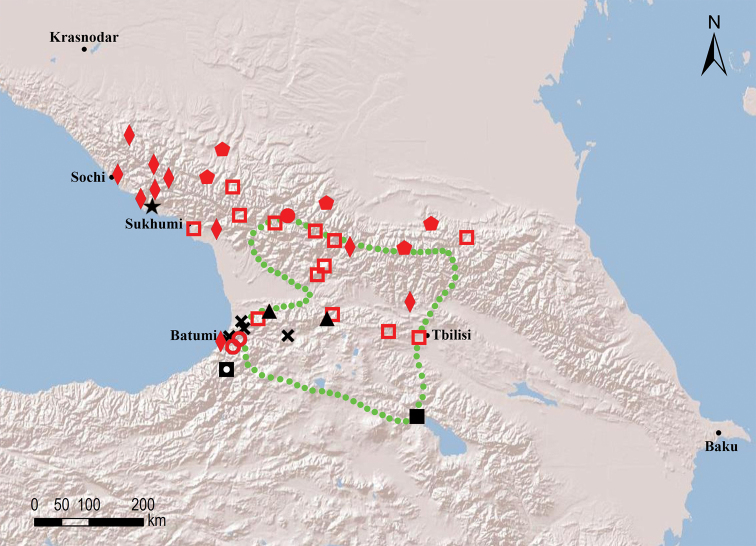
Distribution of the *Omobrachyiuluscaucasicus* (red and green) and *O.sevangensis* (black) species groups in the Caucasus (excluding *O.caucasicus* comb. nov. known from numerous records in the region, and with a distribution range exceeding the map coverage): *O.adsharicus* (ring), *O.curvocaudatus* (diamond), *O.geniculatus* (pentagon), *O.divaricatus* (dotted line indicating the approximate distribution range), *O.macrourus* (open square), *O.unugulis* sp. nov. (filled circle), *O.sevangensis* comb. nov. (filled square), O.cf.sevangensis comb. nov. (filled square with white dot), *O.kvavadzei* sp. nov. (cross), *O.ponticus* sp. nov. (star), and *O.trochiloides* sp. nov. (triangle).

**Map 3.**
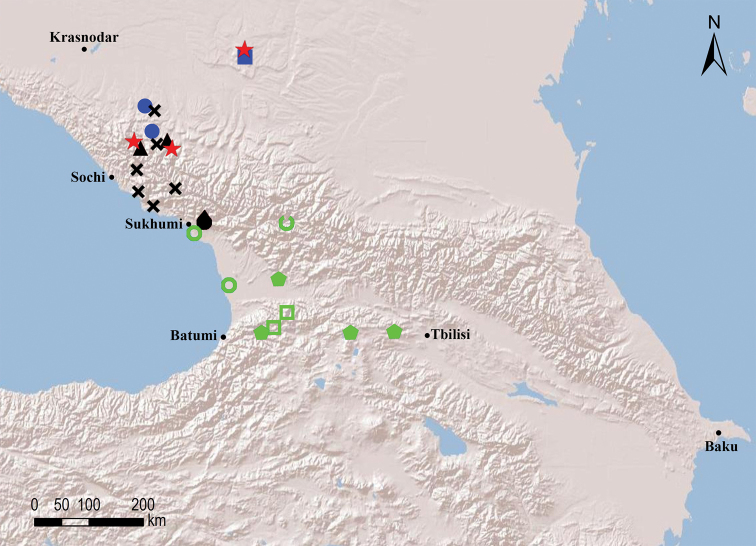
Distribution of the *Omobrachyiulusroseni* (red and blue), *implicitus* (black) and *hortensis* (green) groups: *O.roseni* (filled circle), *O.faxifer* sp. nov. (star), *O.zuevi* sp. nov. (filled square), *O.implicitus* (cross), *O.fasciatus* sp. nov. (triangle), *O.lazanyiae* sp. nov. (drop), *O.hortensis* (ring), O.cf.hortensis (broken ring), *O.armatus* sp. nov. (pentagon), *O.pristis* sp. nov. (open square).

The diversity of the Brachyiulini within the study region is markedly concentrated in the Colchidan biogeographical province (as understood by, e.g., Wulff 1944; Iablokov-Khnzorian 1961; Abdurakhmanov 2017) (Maps 1–3), including the western parts of both Greater and Lesser Caucasus. This is a pattern observed in most other diplopod groups, perhaps except for the glomerid genus *Trachysphaera* Heller, 1858 (Glomerida: Glomeridae) and the julid genera *Leptoiulus* Verhoeff, 1894 (Julida: Julidae) and *Amblyiulus* plus *Syrioiulus* whose Caucasian species are more equally spread from west to east (Golovatch 1990a: maps 1, 2; Evsyukov et al. 2020: map 2; Evsyukov et al. 2021: fig. 16). In contrast, the only Brachyiulini that are present in the eastern parts of the Caucasus are *Byzantorhopalumrossicum* and *Omobrachyiuluscaucasicus*. This likely reflects more the climatic conditions in relatively recent geological times, being mild and moist near the Black Sea and becoming increasingly arid to the east (Kokhia and Golovatch 2018, 2020), rather than ancient palaeogeographical events.

Interestingly, the Caucasus Major’s main ridge does not seem to act as a significant distribution barrier as one might expect. Seven species (excluding the likely anthropochoric *Brachyiulusjawlowskii* and *Megaphyllumhercules*, with the latter’s presence in the Caucasus remaining dubious), viz. *Byzantorhopalumrossicum*, *Cyphobrachyiuluslitoreus*, *Omobrachyiuluscaucasicus*, *O.curvcaudatus*, *O.geniculatus*, *O.implicitus*, and *O.macrourus* occur on both sides of the mountain range, while most of the remaining species are narrow local endemics of various parts of the Caucasus, and are thus restricted by other geographical or ecological factors. The same holds true for the Caucasian millipedes in general. However, the two highly similar species of *Colchiobrachyiulus*, *C.dioscoriadis* and *C.montanus* sp. nov., seem to lead their existence from splitting the population of their ancestral species into two by the summit parts west of Elbrus, these probably becoming inaccessible to this particular brachyiulinine lineage at some point during the global cooling in the late Pliocene.

### Zoogeographical considerations

When we talk about the faunogenesis of the Caucasus region, we have to consider the different origin and development of the Greater (GC) and the Lesser Caucasus (LC). GC existed as an archipelago and later as an isolated landmass surrounded by the Eastern Paratethys at least throughout most of the Miocene, becoming connected to LC only by the end of the epoch, and with the East European Platform not earlier than the late Pliocene. In contrast, LC is an older landmass that has remained connected with Anatolia and Hyrcania through much of the Cenozoic (Popov et al. 2002; Meulenkamp and Sissingh 2003; Golonka 2004). Thus, faunal exchanges must have taken place predominantly from LC to GC, with the former serving as a bridge between Anatolia, Hyrcania and GC. The present disjunct distribution of the genus *Iraniulus* appears to be evidence of that ancient migratory route. Its two known species, *I.fagorum* and *I.tricornis* sp. nov., are conspicuously similar to each other, despite the considerable distance (> 500 km) between Hyrcania and Svanetia. Such a pattern also implies that the genus was once more widespread and speciose, now existing with only a few representatives that have survived in suitable (mild and humid) conditions.

In the light of the aforecited palaeogeographical reconstructions, the genera *Colchiobrachyiulus* and *Svaniulus* gen. nov., each known from two species from GC, likely owe their present endemic status to relatively recent migrations followed by declines and extinctions in their ancestral distribution areas. Such extinction events also might have led to the present condition of the highly distinct subgenus Grusiniulus of the mostly Aegean-Anatolian genus *Cyphobrachyiulus*, which is represented nowadays by a single species, apparently endemic to LC and Transcaucasia. Another Anatolian element in the Caucasian fauna is the Western Caucasian endemic Cyphobrachyiulus (Diaxylus) litoreus, the only representative of the subgenus Diaxylus in the study region.

The presence of several other species in the Caucasus points to relatively recent zoogeographical connections with the Balkan Peninsula. *Byzantorhopalumrossicum* is represented by two subspecies, the nominate one being widespread in the northern Caucasus, eastern Ukraine and Crimea, and *B.r.strandschanum* occurring in the East Balkans (Kime and Enghoff 2017). The validity of the two subspecies put aside, the present distribution of *B.rossicum* obviously results from the species migrating along the northern Black Sea coast; and taking into account the exclusively Balkan-western Anatolian distribution of *Byzantorhopalum* (with the exception of *B.r.rossicum*), we can conclude that the migration seems to have taken place in the clockwise direction. Some Balkan members of *Megaphyllum* s. str. seem to have passed through the same route: The Crimean endemic *Megaphyllumtauricum* is a close sibling of the circum-Rhodopean *M.rhodopinum*, and if the presence of *M.hercules* in the Caucasus is confirmed in the future this would be another case of a disjunct Balkan-Caucasian distribution. Furthermore, the northwest Caucasian endemic *M.spathulatum*, despite its more distinct gonopod morphology, is an obvious member of the *M.unilineatum* group, to which *M.hercule*s, *M.rhodopinum*, and *M.tauricum* also belong (Lazányi and Vagalinski 2013). This northern Balkan-Caucasian migratory pathway was operable only after the regression of the Paratethys and the connection of the Caucasus to the European mainland in the late Pliocene.

The genus *Omobrachyiulus* is one of the large genera within the Brachyiulini. With its 20 species known from the Caucasus alone, it is by far the most speciose brachyiulinine genus in the region. Outside the study region, *Omobrachyiulus* is represented only by four species and one subspecies scattered from the South Carpathians to the Middle East (Vagalinski and Lazányi 2018; Vagalinski and Golovatch 2019). Apart from the morphologically and geographically isolated *Omobrachyiulusmesorientalis* Vagalinski & Golovatch, 2019, endemic to the Levant region, and the Rhodopean *O.beroni* (Strasser, 1973), both representing separate species groups of their own, the remaining non-Caucasian congeners, viz. *O.platyurus* (Latzel, 1884) from western Romania and northeastern Serbia, *O.strasseri* Vagalinski & Lazányi, 2018, and *O.caucasicusthassensis* (Mauriès, 1985) from Aegean islands, can all be ascribed to the *caucasicus* group which includes a further seven species occurring in the Caucasus (see Taxonomic part). And while these are mostly regional endemics (except for *O.adsharicus* which is known only from Ajara), with partly overlapping distributions spanning across much of the Western Caucasus (see Map 2), the three circum-Balkan group members are all narrow local endemics, two of them, *O.C.thassensis* and *O.strasseri*, are known from a single island record each. This picture suggests another case of Balkan-to-Caucasus migrations, again followed by declines and extinctions in the ancestral territory, as well as intense speciation in the newly colonised land. In the case of *Omobrachyiulus*, however, the migratory route likely followed the southern rather than the northern Black Sea coast. The strongest argument for this assumption is the distribution of *O.caucasicus*, which, apart from its Aegean subspeciesthassensis, occurs west of the Caucasus also with its nominate subspecies, which is recorded from northeastern Anatolia (Enghoff 2006). Furthermore, the Rhodopean *O.beroni*, despite being morphologically highly distinct, shares certain characters in common with both the *caucasicus* group and the mostly Lesser Caucasian *sevangensis* group, viz. a small, spiniform, freely protruding part of the anterior process of the opisthomere; and the promere lacking a distal groove, the vulva with the operculum conspicuously exceeding in height the bursa, and a long, pointed epiproct turned ventrad, respectively. The Pontic Mountains in Turkey, which are very marginally explored concerning their millipede fauna, and are characterised by a mild and humid climate, are likely to be inhabited by some yet undescribed species of *Omobrachyiulus*.

### Prospects for future studies

The current study covers much of the Caucasus, giving a near-to-complete view over the diversity of the Brachyiulini in the region. However, single records like those of *Svaniuluswaltheri* gen. nov., sp. nov., *S.ryvkini* gen. nov., sp. nov., *Omobrachyiulusunugulis* sp. nov., and *O.zuevi* sp. nov. suggest that certain narrow local endemics could still await discovery even in areas that have already been the target of collecting efforts. The Armenian and Azerbaijani parts of the Lesser Caucasus remain somewhat less thoroughly explored compared to the Georgian part of the massive and to the Greater Caucasus as a whole. Despite the generally drier climate in the east, one or several new, relict brachyiulinine species may exist there in favourable places like high mountain forests or deep river gorges. And considering that three of the four brachyiulinine (sub)species known only from caves belong to the mostly Caucasian genus *Omobrachyiulus* (the fourth one being *Titanophyllumspiliarum* Akkari, Stoev & Enghoff, 2011), it would not be surprising if *O.lazanyiae* sp. nov. was not the only local member of the genus to occur in the subterranean realm (either as trogloxene or as a specifically adapted form).

## Supplementary Material

XML Treatment for Brachyiulus

XML Treatment for Brachyiulus jawlowskii

XML Treatment for Brachyiulus lusitanus

XML Treatment for Byzantorhopalum

XML Treatment for Byzantorhopalum (Byzantorhopalum) rossicum

XML Treatment for Colchiobrachyiulus

XML Treatment for Colchiobrachyiulus dioscoriadis

XML Treatment for Colchiobrachyiulus montanus

XML Treatment for Cyphobrachyiulus

XML Treatment for Diaxylus

XML Treatment for Cyphobrachyiulus (Diaxylus) litoreus

XML Treatment for Grusiniulus

XML Treatment for Cyphobrachyiulus (Grusiniulus) redikorzevi

XML Treatment for Iraniulus

XML Treatment for Iraniulus fagorum

XML Treatment for Iraniulus tricornis

XML Treatment for Megaphyllum

XML Treatment for Megaphyllum

XML Treatment for Megaphyllum (Megaphyllum) hercul

XML Treatment for Megaphyllum (Megaphyllum) spathulatum

XML Treatment for Omobrachyiulus

XML Treatment for Omobrachyiulus adsharicus

XML Treatment for Omobrachyiulus caucasicus

XML Treatment for Omobrachyiulus curvocaudatus

XML Treatment for Omobrachyiulus divaricatus

XML Treatment for Omobrachyiulus geniculatus

XML Treatment for Omobrachyiulus macrourus

XML Treatment for Omobrachyiulus unugulis

XML Treatment for Omobrachyiulus hortensis

XML Treatment for Omobrachyiulus cf. hortensis

XML Treatment for Omobrachyiulus armatus

XML Treatment for Omobrachyiulus pristi

XML Treatment for Omobrachyiulus implicitus

XML Treatment for Omobrachyiulus fasciatus

XML Treatment for Omobrachyiulus lazanyiae

XML Treatment for Omobrachyiulus roseni

XML Treatment for Omobrachyiulus faxifer

XML Treatment for Omobrachyiulus zuevi

XML Treatment for Omobrachyiulus sevangensis

XML Treatment for Omobrachyiulus kvavadzei

XML Treatment for Omobrachyiulus ponticus

XML Treatment for Omobrachyiulus trochiloides

XML Treatment for Svaniulus

XML Treatment for Svaniulus ryvkini

XML Treatment for Svaniulus waltheri
